# *Tachysphex austriacus* Kohl, 1892 and *T. pompiliformis* (Panzer, 1804) (Hymenoptera, Crabronidae) are a complex of fourteen species in Europe and Turkey

**DOI:** 10.3897/zookeys.577.7301

**Published:** 2016-04-05

**Authors:** Jakub Straka

**Affiliations:** 1 Department of Zoology, Charles University in Prague, Viničná 7, CZ-128 44, Praha 2, Czech Republic

**Keywords:** Hymenoptera, Apoidea, Crabronidae, Tachysphex, taxonomy, new species, description, Europe, Turkey

## Abstract

*Tachysphex pompiliformis* (Panzer, 1804) and *Tachysphex austriacus* Kohl, 1892 species subgroups belong to the *Tachysphex pompiliformis* species group, and both subgroups consist of morphologically similar species. The *Tachysphex austriacus* Kohl species subgroup comprises four species in Europe and Turkey. For this subgroup, differential diagnoses of *Tachysphex austriacus* and *Tachysphex prismaticus* Straka, 2005 are presented, and *Tachysphex hungaricus* **sp. n.** from Hungary and *Tachysphex smissenae* **sp. n.** from Spain, France and Turkey are described. The *Tachysphex pompiliformis* species subgroup consists of ten species from Europe and Turkey. For this subgroup, differential diagnoses of *Tachysphex ferrugineus* Pulawski, 1967 and *Tachysphex opacus* F. Morawitz, 1893, as well as the differential diagnosis and redescription of *Tachysphex pompiliformis*, are presented. *Tachysphex dimidiatus* (Panzer, 1809), *Tachysphex jokischianus* (Panzer, 1809) and *Tachysphex nigripennis* (Spinola, 1808) are resurrected from synonymy with *Tachysphex pompiliformis* and redescribed. Neotypes of *Tachysphex dimidiatus* (Panzer) and *Tachysphex nigripennis* (Spinola) are designed. *Tachysphex bohemicus* **sp. n.** from the Czech Republic; *Tachysphex cretensis* **sp. n.** from Crete, Greece; *Tachysphex nobilis* **sp. n.** from Bulgaria, the Czech Republic, Hungary, Poland, Slovakia and Turkey; and *Tachysphex punctipleuris* **sp. n.** from Bulgaria, Germany, Hungary, Italy, Slovenia and Turkey are described. Identification keys to all species from *Tachysphex pompiliformis* and *Tachysphex austriacus* species subgroups known from Europe and Turkey are presented.

## Introduction

*Tachysphex pompiliformis* (Panzer, 1804) and *Tachysphex austriacus* Kohl, 1892 species subgroups belong to the *Tachysphex pompiliformis* species group. *Tachysphex austriacus* Kohl has been considered to be a sister species to *Tachysphex pompiliformis* (Panzer) (Straka 2004). Later, *Tachysphex austriacus* Kohl, 1892 was recognized to be a cluster of closely related species different from *Tachysphex pompiliformis* (Panzer). Both species are now considered to belong to separate lineages and form two different species subgroups (Straka 2008). Only three species that were placed in the *Tachysphex pompiliformis* subgroup were recognized in the previous revision of *Tachysphex* in the Palearctic region (Pulawski 1971). Species from the *Tachysphex austriacus* subgroup were thought to be conspecific with *Tachysphex pompiliformis*. Most of the species from the *Tachysphex austriacus* subgroup were recently described or restored from synonymy. First, *Tachysphex austriacus* Kohl was resurrected and redescribed by Straka (2004). Subsequently, two other species were described and were considered to be related to *Tachysphex austriacus* Kohl: *Tachysphex prismaticus* Straka, 2005 and *Tachysphex stysi* Straka, 2008 (Straka 2005, 2008). Two new species that belong to the *Tachysphex austriacus* subgroup are described in the present study. In a revision of the Palearctic species (Pulawski 1971), only *Tachysphex pompiliformis* (Panzer), *Tachysphex ferrugineus* Pulawski, 1967, and *Tachysphex opacus* F. Morawitz, 1893 were recognized as valid; all other names from respective groups were considered to be junior synonyms. Subsequently, one additional species, *Tachysphex kaszabi* Tsuneki, 1972 from Mongolia, was described (Tsuneki 1972). Pulawski (1971) presented information on the extensive variability within *Tachysphex pompiliformis* (Panzer) species; however, no species recognition or change in taxonomy was made within this variable species until this time, except for the separation of species of the *Tachysphex austriacus* subgroup. In this study, ten species that belong to this subgroup are recognized in Europe and Turkey. Three species names are restored from synonymy, and four additional new species are described.

## Methods

Material from the following institutions and private collections was examined:

BMNH
 British Museum, (Natural History), London, Great Britain (David Notton) 


CSE
 Christian Schmid-Egger, Berlin, Germany 


IBER
 Institute of Biodiversity and Ecosystem Research, Sofia, Bulgaria (Toshko Ljubomirov) 


JSPC
 Jakub Straka, Praha, Czech Republic 


HNHM
Hungarian Natural History Museum, Budapest, Hungary (Sandor Csősz) 


MRBC
 Martin Říha, Brno, Czech Republic 


NHMW
Naturhistorisches Museum Wien, Wien, Austria (Manuela Vizek) 


NJHC
 Niklas Johansson, Habo, Sweden 


NMPC
National Museum, (Natural History), Praha, Czech Republic (Jan Macek) 


OLML
Oberösterreichisches Landesmuseum, Linz, Austria (Fritz Gusenleitner) 


POTC
 Piotr Olszewski, Toruń, Poland 


PTLC
 Pavel Tyrner, Litvínov, Czech Republic 


USMB
Upper Silesian Museum, Bytom, Poland (Waldemar Żyła) 


WAPC
 Werner Arens, Bad Hersfeld, Germany 


WSKC
 Wolfgang Schlaefle, Kaiseraugst, Switzerland 


ZIN
Zoological Institute, Russian Academy of Sciences, St. Petersburg, Russia (Yulia Astafurova) 


ZKZC
 Zdeněk Karas, Zliv, Czech Republic 


ZSM
 Staatliche Naturwissenschaftliche Sammlungen Bayerns, Zoologische Staatssammlung, Munich, Germany (Stefan Schmidt) 


Morphological terms are used according to Bohart and Menke (1976) and Krombein and Pulawski (1994), except morphological description of volsella. There is volsellar corpus – the main part with setae on outer margin and a depression on inner side, which connecting volsella with gonocoxites; apical volsellar process – a thin process which bears setae on its outer margin, a row of setae continues from the corpus; dorsal volsellar process – the process, which can have various shapes and extending dorsally from the corpus. The following abbreviations are used in the morphological descriptions:

WML
 clypeus median lobe width 


LCL
 clypeus maximum length 


WCL
 clypeus width 


LA3
 length of antennal article III, dorsally 


WA3
 width of antennal article III, dorsoapically 


LA5
 length of antennal article V, dorsally 


WA5
 width of antennal article V, dorsoapically 


WV
 vertex width 


LV
 vertex length 


MOD
 diameter of median ocellus 


LF1
 forefemur length 


WF1
 forefemur width, laterally 


All of the newly described species were labeled in the following manner: “HOLOTYPUS ♂ or ♀, name of taxon sp. nov., J. Straka det. 2015” on a red card; neotypes were designated on red cards, and paratypes were designated on yellow cards. Exact label data are cited only for the holotypes. Separate lines on the labels are indicated with a slash “/”, and separate labels are indicated with double slashes “//”.

## Results

### *Tachysphex austriacus* species subgroup

**Diagnosis of subgroup.** The *Tachysphex austriacus* species subgroup belongs to the *Tachysphex pompiliformis* group, which is characterized by the following character combination: Labrum flat; frons uniformly convex; scutum and mesopleuron finely reticulate or punctate, microsculptured or unsculptured among punctures; episternal ridge developed; tarsomeres V of mid and hind legs unmodified, straight ventrally and mildly convex dorsally; tarsomeres IV of the mid and hind legs distinctly emarginated and longer than wide; anal lobe of the hindwing small; sternum I without a longitudinal carina; sterna without distinct setal patches; pygidium triangular, narrowly truncate at the apex, with indistinct transverse carina, integument sparsely punctate, or without punctures in some species, interspaces between punctures finely sculptured to unsculptured. In males, the volsellar corpus is not separated from the dorsal process by constriction, and thus the corpus is extended to a process; aedeagus with distinct well-developed teeth, usually not more than 10 in number.

From the other species of the *Tachysphex pompiliformis* group, the *Tachysphex austriacus* species subgroup (species complex *sensu* Straka 2005) differs in having the following characters: glossa and galea short, galea as wide as long, apex densely setose; glossa shorter than galea; mesopleuron microsculptured, dull or slightly shiny, with or without punctures, punctures ill-defined when present, and with distinct interspaces; fore and midfemora uniformly sculptured throughout, with only small punctures and uniform interspaces; lateral parts of tergum II with variably sparse punctuation and shiny interspaces; and terga I-III with silvery apical fasciae (rarely absent). In the female, clypeus with lateral incisions and with shallow or absent median emargination. In the male, clypeus arcuate with conspicuous lip corner and forebasitarsus without rake. The *Tachysphex austriacus* species subgroup differs from the *Tachysphex pompiliformis* species subgroup by two consistent male characters. In the *Tachysphex austriacus* species subgroup, the forefemoral notch, slightly shiny to shiny, has no elevated plate and carinae on the margins and has distinct small setae on the surface; the setae on the volsellar apical process are uniformly directed ventrally (or nearly so). In the *Tachysphex pompiliformis* species subgroup, an elevated plate is present in the forefemoral notch with more or less distinct carinae on the margins; its surface appears nearly glabrous, finely or coarsely microsculptured; the ventral setae on the volsellar apical process are randomly directed. Characters for distinguishing females of *Tachysphex pompiliformis* and *Tachysphex austriacus* subgroups have not been identified so far.

**Species included.** *Tachysphex austriacus* Kohl, 1892; *Tachysphex hungaricus* sp. n.; *Tachysphex pekingensis* Tsuneki, 1971 (China); *Tachysphex prismaticus* Straka, 2005; *Tachysphex smissenae* sp. n.; and *Tachysphex stysi* Straka, 2008 (Central Asia).

#### Tachysphex austriacus

Taxon classificationAnimaliaHymenopteraCrabronidae

Kohl, 1892

Figs 28 , 38 , 41 , 50 , 53 , 62 , 77 , 86 , 91

##### Type material.

Holotype: ♀, Austria: Wien: Türkenschanze, 12.viii.1895, A. Handlirsch lgt. “12.8.95. / Handl. // Austr. inf. / Türkenschanz // austriacus / K. / det. Kohl Type”, [handwriting, name of collector and word “det.” printed]. Holotype in NHMW, examined.

##### Additional material examined.

**Czech Republic**: Boh. centr., Lysá nad Labem env., 1 ♂, 1 ♀, 8. vi. 1950, A. Hoffer lgt., OLML; Mor. mer., Dolní Bojanovice, 1 ♂, 1 ♀, viii. 1942, A. Hoffer lgt., JSPC; Hodonín, 1 ♀, vii. 1942, A. Hoffer lgt., OLML; **Germany**: Birkenheide env., 1 ♂, 2. vi. 1993, O. Niehuis lgt., JSPC; Ingelheim-nord, Mainz, 1 ♀, 24. vii. 1993, Ch. Schmid-Egger lgt., JSPC; Mallnow, Frankfurt an der Oder 13 km NNW, Brandenburg, 1 ♂, 24. vii. 2008, Ch. Schmid-Egger lgt., CSE; **Hungary**: Ör Sz. Miklós, Őrtilos Szentmihályhegy, Somogy, 1 ♀, 21. vi. 1920, Sajó lgt., HNHM; Kecskemét env., 1 ♀, 16. vii. 2006, J. Halada lgt., JSPC; Kecskemét, Nyomás, 1 ♀, 18. vii. 1962, Sólymosné lgt., HNHM; Kecskemét, puszta, 1 ♂, 23. 6. 1987, J. Halada, OLML; Oktatasi Központ, Fülophaza env., sand dunes, 1 ♀, 16. vii. 2013, J. Habermannová & J. Straka lgt., JSPC; Örkeny, puszta, 1 ♀, 16. viii. 2000, J. Straka lgt., 1 ♂, 7. vi. 2013, D.Benda, P.Bogusch & J.Straka lgt., JSPC; Ásotthalom Mcs., Szeged, 1 ♀, 3. vii. 1973, L. Móczár lgt., HNHM; **Kazakhstan**: Matay desert, Sarkand distr., 3 ♀♀, 25. vi. 1995, J. Halada lgt., OLML; **Mongolia**: Arvaykheer 137 km NE, Överkhangay prov., dunes, 4 ♂♂, 8 ♀♀, 2. vii. 2004, 6 ♀♀, 25. vii. 2004, J. Straka lgt., JSPC; Bayankhongor 2 km S, Bayankhongor prov., along Tüy Gol, 1 ♀, 10. vii. 2004, J. Straka lgt., JSPC; Ulaanbaatar 170km W, dunes, 1 ♀, 16. viii. 2007, J. Halada, JSPC; **Poland**: Kików, Malopolska Upland, 1 ♂, 2 ♀♀, 14. vii. 1996, R. Dobosz lgt., 9 ♂♂, 2 ♀♀, 19. vi. 1995, W.Żyła lgt., USMB, JSPC; Małkinia, 1 ♀, 14. vii. 1995, R. Dobosz lgt., USMB; Sierakowo, 1 ♂, 21. vi. 2011, P. Olszewski lgt., POTC; Toruń-Glinki, 1 ♀, 14. vi. 2011, P. Olszewski lgt., POTC; Toruń-poligon artyl., 1 ♀, 14. vi. 2011, P. Olszewski lgt., POTC; Wigry, Wigierski NP, 1 ♀, 5. viii. 2002, W. Żyła lgt., USMB; **Slovakia**: Chľaba 1.5km SE, 1 ♀, 5. viii. 2013, M. Říha lgt., MRBC; Chotín, 1 ♂, 12. vii. 1981, P. Tyrner lgt., PTLC, 1 ♀, 1. viii. 1960, 1 ♀, 30. vii. 1970, 1 ♀, 27. vii. 1974, 1♂, 13. vii. 1962, Z. Pádr lgt., 1♂, vi. 1977, M. Kocourek lgt., OLML, JSPC; Somotor, 1 ♂, vii. 1960, M. Kocourek lgt., OLML.

##### Diagnosis.

*Tachysphex austriacus* is the type species of the *Tachysphex austriacus* species subgroup. *Tachysphex austriacus* Kohl differs from all other species from the subgroup in the following combination of characters: ♂, ♀: gena narrow, conspicuously converging behind compound eyes; scutum and scutellum sparsely punctate, punctures well developed and usually up to 1.5 diameter apart (more than that in some specimens); ventral part of mesopleuron densely punctate, punctures relatively large and well developed in comparison to other species; trochanters densely punctate; ♂: ventral volsellar setae in one line; ♀: clypeal lip with distinct irregular median emargination; clypeal bevel forming slightly convex to flat plate, but the transition between the clypeal basomedian area and the bevel is not angulated; forebasitarsal rake pale, with four apical spines.

##### Geographic Distribution.

Austria, Czech Republic, Germany, Hungary, Kazakhstan, Mongolia, Poland and Slovakia.

##### Note.

The holotype was redescribed by Straka (2004).

#### Tachysphex hungaricus sp. n.

Taxon classificationAnimaliaHymenopteraCrabronidae

http://zoobank.org/A304520F-F80F-41AE-936D-FBDCF1436EEE

**Figures 13–21.**
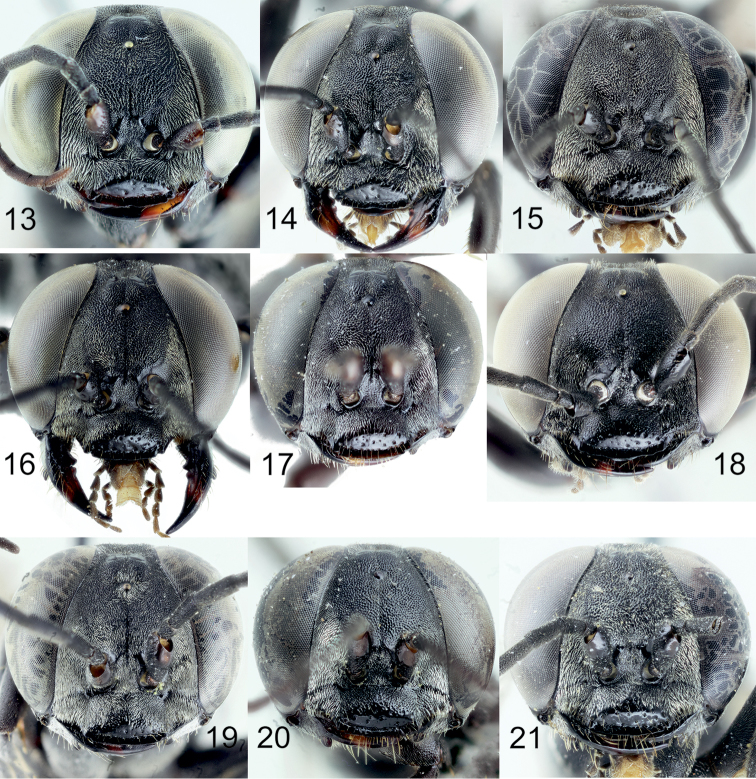
Head of female, front view. **13** *Tachysphex hungaricus* sp. n. **14** *Tachysphex smissenae* sp. n. **15** *Tachysphex bohemicus* sp. n., **16** *Tachysphex dimidiatus* **17** *Tachysphex jokischianus* **18** *Tachysphex nigripennis* **19** *Tachysphex nobilis* sp. n. **20** *Tachysphex pompiliformis* **21** *Tachysphex punctipleuris* sp. n.

**Figures 36–37.**
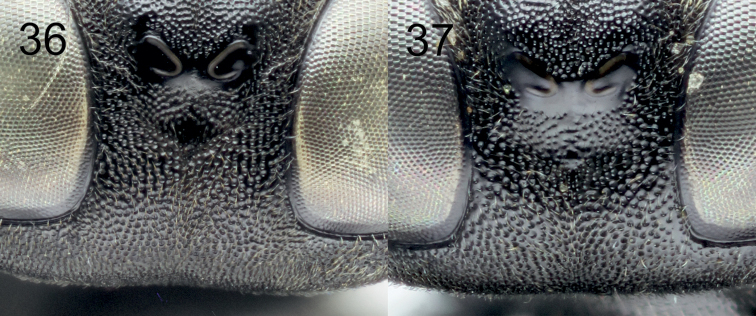
Vertex of female, detail. **36** *Tachysphex hungaricus* sp. n. **37** *Tachysphex smissenae* sp. n.

Figs 13 , 36 , 42 , 54 , 78 , 79

##### Type material.

Holotype: ♀, Hungary: “HU; Hungary, 8.vii.2006 / Örkeny, / 50km SE Budapest / J. Straka lgt., EtOH 96% // ZSM / HYM 23722”. Holotype in NMPC. Paratype: Hungary: Pákozd env., pasture stepe, 1 ♀, 10. vi. 2015, M. Halada lgt., JSPC.

##### Diagnosis.

The female of *Tachysphex hungaricus* sp. n. resembles *Tachysphex austriacus* Kohl, *Tachysphex prismaticus* Straka and *Tachysphex smissenae* sp. n. and also *Tachysphex jokischianus* Panzer and *Tachysphex punctipleuris* sp. n. from the *Tachysphex pompiliformis* species subgroup in the sculptures and shape of the clypeus. This new species is the most similar to *Tachysphex prismaticus*. They share a characteristic form of the clypeus with nearly angulated transition between the basomedian area and a nearly flat bevel; vertex punctures without interspaces medially and at least one diameter apart laterally; very short setae of frons, vertex and scutum, about 0.3 × MOD; and uniform microareolate sculpture of propodeal dorsum with longitudinal ridges all over. *Tachysphex hungaricus* sp. n. differs from *Tachysphex prismaticus* in its much denser punctation all over the body; its scutum, trochanters and terga are not sparsely punctate. Females of the *Tachysphex hungaricus* thus resemble species of the *Tachysphex pompiliformis* species subgroup, but differ from them in having the characters, which were specified as synapomorphies with *Tachysphex prismaticus* in their comparison. It is the form of clypeus, sculpture of vertex and propodeum and length of vertex setae.

##### Description of female (holotype).

Body length: 8.2 mm.

Head. Clypeus conspicuously convex, highly elevated, its nearly angular top located slightly dorsally from clypeal midlength; basomedian area well developed, as densely and finely punctate as lateral section, with intermixed large punctures; bevel nearly flat, conspicuously declining, with few large punctures, much longer medially than laterally, but not reaching base of clypeus, bright shiny; lip very slightly arcuate to nearly straight, with median emargination and well developed lateral incisions, separated from bevel by distinct punctate groove, WML:LCL = 1.8, WCL:WML = 1.7. Supraclypeal area flat, finely, densely punctate, punctures ill-defined, interspaces between punctures finely microsculptured, dull. Supraantennal tubercle small, slightly elevated on inner side. Antenna relatively short, LA3:WA3 = 2.2, LA5:WA5 = 2.7. Frons irregularly, very densely punctate, punctures well defined, about half diameter apart, interspaces variable in size, slightly shiny; frontal median line ill defined, not impressed; frontal setae very short, about 0.3 × MOD. Vertex punctate, most punctures well defined, punctures without interspaces medially, but about one diameter apart laterally, punctures laterally slightly larger than medially, interspaces unsculptured, bright shiny. Vertex setae very short, semierect to apressed, about 0.3 × MOD; postocellar impression well developed, widely Y-shaped; vertex slightly wider than long; WV:LV = 1.3. Gena dorsally well developed.

Mesosoma. Scutum without distinct anterior impression; scutum and scutellum with well defined punctures variable in size, less than one diameter apart, interspaces variable in size, shiny, setae gold-brown, about 0.5 × MOD or less. Mesopleuron coarsely microsculptured, with indistinct punctures evanescent in microsculpture; hypoepimeral area finely rugose to coarsely microsculptured, impunctate; ventral part of mesopleuron, with ill-defined punctures, one to less than one diameter apart, interspaces well developed, slightly shiny. Propodeal dorsum uniformly sculptured, very finely areolate with fine irregular longitudinal ridges on entire surface, dull; propodeal side regularly longitudinally ridged, ridges fine, but evenly developed all along, microsculptured, slightly shiny; posteromedial margin of dorsum elevated, produced above marginal ridges, marginal ridges positioned nearly horizontally above groove on posterior side. Legs, including trochanters, densely punctate, punctures small; forebasitarsal rake pale yellowish, with three apical spines, one preapical spin and two other additional spines. Wings slightly infumate, yellowish, with brown veins.

Metasoma. Terga I-III with slightly developed, but distinct silvery apical fasciae, densely, very finely micropunctate, punctures ill-defined, evanescent in microsculpture, interspaces aciculate, slightly shiny; apical depressions shallow with indistinct micropunctures; sculpture of terga VI-V distinctly coarser than on previous terga, punctures denser, ill-defined, but large, one to less than half diameter apart, interspaces microsculptured, apical depression of tergum V coarsely microsculptured, with few scattered punctures. Pygidium of usual size, sparsely punctate, large and minute punctures intermixed, ill defined, but large punctures deep, interspaces nearly unsculptured, shiny. Central part of sternum II with several larger punctures, interspaces microsculptured, shiny; lateral part slightly shiny, densely micropunctate; remaining sterna with uniform sculpture similar to that on sternum II, but more or less reduced laterally.

Coloration. Large area in center of mandibles, terga and sterna I-III and base of tergum and sternum IV red. Distal tarsomeres, apex of pronotal lobe, ventral part of distal antennal segments, apex of clypeal bevel and tip of pygidium dark red. Tegulae reddish translucent. Apical parts of terga I-III slightly translucent. Remaining body parts all black.

##### Geographic distribution.

Known only from Hungary.

##### Note.

Male unknown.

##### Name derivation.

The species is named after the country of origin.

#### Tachysphex prismaticus

Taxon classificationAnimaliaHymenopteraCrabronidae

Straka, 2005

##### Type material.

Holotype: ♂, Kazakhstan: “Kazakhstan 20km / SE Aksay env / 16. – 19. 6. 1992 / leg.K.Denes”, printed label. Holotype in OLML, examined.

##### Diagnosis.

*Tachysphex prismaticus* Straka resembles *Tachysphex austriacus* Kohl, *Tachysphex hungaricus* sp. n., and *Tachysphex smissenae* sp. n. in the shape of the clypeus and well developed punctures on the ventral part of the mesopleuron. It differs from the other species of the subgroup in the following character combination: ♂, ♀: mesopleuron rugose to densely punctate; venter of all trochanters with punctures several diameters apart, their interspaces unsculptured; all femora and tibiae black; ♂: volsella with large dorsal process; ventral volsellar setae in one line; ♀: clypeal lip with distinct irregular median emargination; clypeal bevel forming flat plate, separated from basomedian clypeal area by relatively sharp, more or less angulated transition; scutum and scutellum sparsely punctate, punctures up to two diameters apart at scutum center; forebasitarsal rake with four apical spines.

##### Geographic distribution.

Kazakhstan, Kyrgyzstan and Turkey.

##### Note.

Full description and all material is given in Straka (2005).

#### Tachysphex smissenae sp. n.

Taxon classificationAnimaliaHymenopteraCrabronidae

http://zoobank.org/32C67CA4-285B-4AD8-88FF-8870428E866C

Figs 1 , 14 , 37 , 43 , 55 , 81 , 82 , 92

##### Type material.

Holotype: ♂, France: “F-(84) Bédoin, Sanddünen / 13 km NE Carpentras / v.d.Smissen leg.31.5.2000 // Tachysphex / austriacus / ♂ Kohl / Jakub Straka det.2006”. Holotype in NMPC. Paratypes: **France**: Bédoin, 13 km NE Carpentras, sanddünen, 1 ♂, 31. v. 2000, 2 ♂♂, 3 ♀♀, 14. vi. 2001, 1 ♀, 21. vi. 2004, J. v. d. Smissen lgt., USMB, JSPC; **Spain**: Aranda de Duero, Burgos, 1 ♂, 11. vii. 1974, Z. Bouček lgt., BMNH; Segovia, 2 ♂♂, 28. vi. 1989, W. Schlaefle lgt., WSKC, JSPC; **Turkey**: Karakus Dagi centr., Isparta prov., 1 ♂, 11. vii. 2006, J. Halada lgt., JSPC.

##### Diagnosis.

*Tachysphex smissenae* sp. n. resembles *Tachysphex hungaricus* sp. n. and *Tachysphex prismaticus* Straka in the shape of the clypeus: the limit between the basomedian area and the bevel appears angulated, although less so than in the other two species. This new species also has short setae (0.5 × MOD) on the frons, vertex and scutum, similar to the other two species. The sculpture of the propodeum, however, is coarser than in the other two species (it is areolate and not microareolate between longitudinal ridges), the sculptures of the whole body is coarser and denser and the vertex is normally sculptured, unlike *Tachysphex hungaricus*. The female differs from all other species of the *Tachysphex austriacus* and the *Tachysphex pompiliformis* subgroups by angulated shape of clypeus, sculpture of propodeum, short setae, gena robustly built, mesopleuron coarsely punctate, with relatively large and ventrally well developed punctures (similar to *Tachysphex austriacus*), terga densely punctate and posterior segments also coarsely punctate with apical depression of tergum V nearly impunctate and the pygidium relatively wide and shiny. The male has the forefemoral notch and the volsella typical for the subgroup (similar to *Tachysphex prismaticus* in shape), a conspicuously convex clypeus, which resembles that of the female (except the lip), densely and coarsely punctate terga and coarsely punctate mesopleuron, as in the female.

##### Description of male (holotype).

Body length: 6.7 mm.

Head. Clypeus markedly convex, medially uniformly curved, top at clypeal midlength; basomedian area large, densely punctate; bevel well developed, nearly flat and declining, bright shiny, with several larger punctures; lip conspicuously arcuate, well developed medially, with well-developed, nearly rectangular, lateral corner, lip separated from bevel by distinct groove with large punctures; WML:LCL = 1.3, WCL:WML = 2.4. Supraclypeal area slightly concave with ill defined punctures in microsculpture, dull. Supraantennal tubercle small, slightly elevated on inner side. Antenna relatively short, LA3:WA3 = 1.6, LA5:WA5 = 2.1. Frons uniformly punctate, punctures well defined, one to less than half diameter apart, interspaces variable in size, shiny; frontal median line ill defined, punctate and slightly impressed. Vertex punctate, punctures well defined, with distinct, but small interspaces medially and about one diameter apart close to inner eye margin, punctures laterally slightly larger than medially, interspaces unsculptured, bright shiny. Vertex setae semierect, less than 0.5 × MOD long; postocellar impression well developed, relatively deep, but not sharply delimited, obtusely V-shaped; vertex slightly wider than long; WV:LV = 1.4. Gena dorsally well developed.

Mesosoma. Scutum without distinct anterior impression; scutum and scutellum densely punctate, punctures well defined, most punctures less than half diameter apart, interspaces distinct, unsculptured, slightly shiny, setae about 1.0 × MOD or less. Mesopleuron coarsely punctate to rugose, punctures well defined in lower part and ventrally, but indistinct dorsally, interspaces small to absent, slightly shiny; hypoepimeral area densely punctate to uniformly rugose, dull. Propodeal dorsum coarsely sculptured with irregular longitudinal ridges, uniformly rugose to areolate between ridges; propodeal side longitudinally ridged, ridges well developed, interspaces slightly shiny; posteromedial margin of propodeal dorsum slightly elevated, marginal ridges nearly parallel above groove on posterior side. Legs densely punctate, punctures small; forefemoral notch small, but deep, nearly semicircular, shorter than distance that separates it from forefemoral base, central part of notch without microsculpture, with distinct small setae, shiny. Wings not infumated, with brown veins.

Metasoma. Terga I-III with silvery apical fasciae. Apical depressions of all terga shallow, with micropunctures. Terga I-III densely and distinctly micropunctate, punctures ill defined, interspaces microsculptured, slightly shiny to dull; sculpture of tergum IV-VII coarser than on other terga, also punctures denser. Sterna uniformly punctate, nearly like terga.

Coloration. Apical part of mandibles and terga I and II red. Distal tarsomeres, forefemoral notch, sternum I and base of sternum II dark red. Tegulae reddish translucent. Apical parts of all terga slightly translucent. Remaining body parts black.

Variation of males. Body length: 6.5–7.0 mm. Clypeus slightly to conspicuously convex medially. Forefemoral notch and all metasoma dark in some specimens.

##### Description of female.

Body length: 7.6–8.2 mm.

Head. Clypeus conspicuously convex, highly elevated, its top slightly dorsally from clypeal midlength; basomedian area large, densely and finely punctate as in lateral section, with intermixed large punctures; bevel nearly flat, markedly declining, with few large, sparse punctures, much longer medially than laterally, not reaching base of clypeus, bright shiny; lip very slightly arcuate to nearly straight, with small median emargination and well developed lateral incisions, separate from bevel by distinctly punctate groove, WML:LCL = 1.7, WCL:WML = 1.8. Supraclypeal area slightly concave, very finely punctate, punctures ill defined, interspaces between punctures nearly unsculptured, shiny. Supraantennal tubercle small, slightly but distinctly elevated on inner side. Antenna relatively short, LA3:WA3 = 2.2, LA5:WA5 = 2.7. Frons densely, uniformly punctate, punctures well defined, about half diameter apart or less, interspaces variable in size, slightly shiny; frontal median line ill defined, punctate in dorsal half, indistinctly impressed; frontal setae very short, about 0.3 × MOD. Vertex punctate, punctures well defined, with distinct, but small interspaces medially and about one diameter apart close to inner eye margin, punctures laterally slightly larger than medially, interspaces unsculptured, bright shiny. Vertex setae very short, laterally semierect, about 0.5 × MOD, medially apressed, about 0.3 × MOD; postocellar impression well developed, widely Y-shaped; vertex slightly wider than long; WV:LV = 1.3. Gena dorsally well developed.

Mesosoma. Scutum without distinct anterior impression; scutum and scutellum densely punctate, punctures well defined, uniform in size, about half diameter apart (up to one diameter apart in some specimens), interspaces uniform in size, slightly shiny, setae about 0.5 × MOD or less. Mesopleuron coarsely punctate to rugose, punctures well defined in lower parts and ventrally, but indistinct dorsally, interspaces small to absent, slightly shiny; hypoepimeral area densely punctate, with punctures ill-defined, appearing uniformly rugose, dull; ventral part of mesopleuron with punctures well defined, interspaces shiny. Propodeal dorsum coarsely sculptured, uniformly rugose to areolate, with irregular longitudinal ridges; side longitudinally ridged, ridges well developed, interspaces slightly shiny; posteromedial margin of dorsum slightly elevated, marginal ridges nearly parallel, but turning ventrally and directed toward groove on posterior surface. Legs, including trochanters, densely punctate, punctures small; forebasitarsal rake pale yellowish, with reddish iridescence, with three apical spines, one preapical spin and two or three additional spines. Wings slightly yellowish, veins brown.

Metasoma. Terga I-III with silvery apical fasciae; densely and very finely micropunctate, punctures ill defined, evanescent in microsculpture, interspaces very finelly microsculptured, slightly shiny; apical depressions shallow, with indistinct micropunctures; sculpture of terga IV-V distinctly coarser than on previous terga, also punctures denser; terga IV-V coarsely punctate, punctures ill-defined, but large, one to less than half diameter apart, interspaces microsculptured, apical depression of terga IV and V coarsely microsculptured, apical depression of tergum IV very finely punctate, apical depression of tergum V with a few scattered punctures, slightly shiny, but coarsely microsculptured and dull in some specimens. Pygidium relatively wide, sparsely punctate, with large and minute punctures intermixed, large punctures well defined, deep, interspaces nearly unsculptured, shiny. Central part of sternum II with several larger punctures, interspaces microsculptured, shiny; lateral part slightly shiny, densely micropunctate; remaining sterna with uniform sculpture similar to that on sternum II, but more or less reduced laterally.

Coloration. Central part of mandibles and terga and sterna I-II red. Distal tarsomeres dark reddish. Tegulae brown translucent. Apical parts of terga I-III slightly translucent. Remaining body parts black.

##### Geographic distribution.

France, Spain, Turkey.

##### Name derivation.

The species is named in honor of the outstanding entomologist and collector of most of the type specimens, Jane van der Smissen.

### *Tachysphex pompiliformis* species subgroup

**Diagnosis of subgroup.** The *Tachysphex pompiliformis* species subgroup differs from the *Tachysphex austriacus* species subgroup by two male characters. In the *Tachysphex pompiliformis* species subgroup, the surface of the forefemoral notch is microsculptured (very finely in some species), slightly elevated, with a distinct carina on the inner margin, sometimes on the outer margin as well; and the setae on the lower surface of the forefemoral notch are hardly visible (magnification 50 ×). The ventral setae on the volsellar apical process are randomly directed. In the *Tachysphex austriacus* species subgroup, the forefemoral notch is slightly shiny to shiny, without an elevated plate and carinae on the margins and with distinct small setae on the surface; the setae on the volsellar apical process are uniformly directed ventrally (or nearly so). Characters for distinguishing females of *Tachysphex pompiliformis* and *Tachysphex austriacus* subgroups have not been identified so far. See the characterization under the diagnosis of the *Tachysphex austriacus* species subgroup.

**Species included.** *Tachysphex bohemicus* sp. n.; *Tachysphex cretensis* sp. n.; *Tachysphex dimidiatus* (Panzer, 1809); *Tachysphex ferrugineus* Pulawski, 1967; *Tachysphex jokischianus* (Panzer, 1809); *Tachysphex kaszabi* Tsuneki, 1972 (Mongolia); *Tachysphex nigripennis* (Spinola, 1808); *Tachysphex nobilis* sp. n.; *Tachysphex opacus* F. Morawitz, 1893; *Tachysphex pompiliformis* (Panzer, 1804); and *Tachysphex punctipleuris* sp. n.

#### Tachysphex bohemicus sp. n.

Taxon classificationAnimaliaHymenopteraCrabronidae

http://zoobank.org/00DC3836-D9BD-485D-A2E4-C8591D94CD0C

Figs 2 , 15 , 29 , 59 , 63 , 70 , 80 , 87 , 93 , 106

##### Type material.

Holotype : ♂, Czech Republic: “CZ; Boh. bor., / 7.-19.vii.2013 / Okna, Doksy env. / J. Straka lgt., EtOH 96% // BC ZSM / HYM 24253”. Holotype in NMPC. Paratype: 1 ♀, same locality as holotype, with label BC ZSM, HYM 24597.

##### Diagnosis.

*Tachysphex bohemicus* sp. n. is most similar to *Tachysphex jokischianus* in having nearly impunctate apex of tergum V and wide pigydium with shiny surface. It also resamble *Tachysphex dimidiatus* in the overall sparsely micropunctate metasoma, which makes the appearance of metasomal sculptures more shiny than in other compared species from the *Tachysphex pompiliformis* species subgroup. The apex of the male volsella of the new species is sparsely setose and may resemble volsella of males from the *Tachysphex austriacus* species subgroup. Both sexes of *Tachysphex bohemicus* sp. n. are easily recognizable by a slightly prominent supraantennal tubercles, which are separated by punctate area; the tubercle is distinctly punctate on the top and nearly at all sides. This character is unique in the *Tachysphex pompiliformis* species subgroup. Like *Tachysphex jokischianus*, the apical depression of tergum V is nearly impunctate medially, but the terga are finely and sparsely punctate and the pygidium is basally microsculptured; the metasoma thus resembles that of *Tachysphex dimidiatus*, except for the apical depressions of all terga, which are very sparsely punctate and well impressed. *Tachysphex bohemicus* sp. n. possess an unusual combination of characters: the propodeum lack longitudinal ridges dorsally and laterally, the female clypeus is distinctly arcuate at apex and uniformly convex dorsally, and the male forefemoral notch is black, positioned more anteriorly than in the other species of the subgroup and its proximal margin is rounded and elevated over the distal margin.

##### Description of male (holotype).

Body length: 7.3 mm.

Head. Clypeus uniformly convex, distinctly elevated in midlength; basomedian area large, densely, distinctly punctate; bevel small, decreasing toward clypeal lip, shiny, sparsely punctate; lip uniformly, distinctly arcuate, not longer medially than laterally, with distinct lateral corner, lip separated from bevel by a groove; WML:LCL = 1.5, WCL:WML = 2.2. Supraclypeal area flat, distinctly punctate, punctures well defined, interspaces between punctures slightly shiny. Supraantennal tubercle small, rounded, distinctly punctate on top, nearly at all sides; area between the tubercles is also distinctly punctate, interspaces dull. Antenna relatively short, LA3:WA3 = 1.6, LA5:WA5 = 2.0. Frons uniformly punctate, punctures well defined, less than one diameter apart, interspaces variable in size, shiny; frontal median line inconspicuous, not impressed. Vertex sparsely punctate, punctures well defined, less than half to two diameters apart, interspaces slightly microsculptured, slightly shiny to dull. Vertex setae semierect medially, nearly erect close to inner eye margin, almost 1 × MOD long, postocellar impression large, and deep, but with ill defined margins, open widely U-shaped, sparsely punctate, punctures relatively large; vertex, WV:LV = 1.7. Gena dorsally well developed.

Mesosoma. Scutum without distinct anterior impression; scutum and scutellum densely punctate, punctures well defined, most punctures less than half diameter apart, interspaces distinct, anteriorly microsculptured, slightly shiny to dull, setae less than 1.0 × MOD long. Mesopleuron sparsely punctate anteriorly, posteriorly impunctate, interspaces microsculptured, dull; hypoepimeral area coarsely microsculptured, without distinct punctures, dull; ventral part of mesopleuron with small ill-defined punctures, interspaces finely microsculptured, slightly shiny. Propodeal dorsum coarsely, irregularly sculptured, longitudinally ridged only basally, irregularly ridged medially; propodeal side microsculptured, without longitudinal ridges, slightly shiny; posteromedial margin of dorsum inconspicuously elevated, marginal ridges inconspicuous, slightly ventromedially directed toward groove. Legs densely punctate, punctures small; forefemoral notch small, well developed, but not deep, positioned slightly anteriorly, shorter than distance that separates it from forefemoral base; proximal margin elevated over distal margin, but widely rounded; anterior and posterior side lined by well developed ledge, notch surface without distinct setae, slightly microsculptured, dull. Wings distinctly infumate, but not dark, with brown veins.

Metasoma. Silvery apical fasciae developed faintly on terga I-IV. Apical depressions of terga distinct, sparsely micropunctate, nearly like more anterior parts. Terga I-IV sparsely micropunctate, punctures ill defined, interspaces coarsely microsculptured, slightly shiny to dull; sculpture of terga IV-VII slightly more conspicuous than that of other terga, also densely punctate. Sterna uniformly punctate, nearly like terga. Volsella light brown, ventral setae pointing in various directions in medial part, but almost in one direction at apical process; apical process relatively sparsely setose; dorsal process large, wider than corpus; corpus lined with distinct ledge along base of setae on inner side of corpus.

Coloration. Apical part of mandibles, distal tarsomeres and terga I-III red. Forefemoral notch black. Tegulae reddish translucent. Apical parts of all terga slightly translucent. Remaining body parts black.

##### Description of female.

Body length: 10.3 mm.

Head. Clypeus distinctly convex, top at clypeal midlength; basomedian area relatively large, more sparsely punctate and punctures larger than on lateral section, interspaces between punctures well developed, shiny, larger medially than laterally, punctures mostly less than one diameter apart; bevel large, distinctly convex, medially longer than basomedian area, laterally much shorter, obtusely triangular, with numerous large, sparse punctures, not reaching base of clypeus, shiny; lip distinctly arcuate, with small lateral incisions, without medial emargination, separate from bevel by punctate groove, WML:LCL = 1.6, WCL:WML = 1.9. Supraclypeal area flat, distinctly punctate, punctures well-defined, interspaces shiny. Supraantennal tubercle small, rounded, distinctly punctate on top, nearly at all sides; area between the tubercles is also distinctly punctate, interspaces dull. Antenna relatively short, LA3:WA3 = 2.3, LA5:WA5 = 2.6. Frons uniformly punctate, punctures well defined, about half diameter apart or less; frontal median line distinct, well impressed in lower frons. Vertex punctate, punctures well defined, less than half to one diameter apart, laterally finer and denser than medially, interspaces microsculptured, shiny. Vertex setae semierect mesally, nearly erect close to inner eye margin, shorter than 1 × MOD; postocellar impression deep, widely opened, V-shaped; vertex moderately wider than long; WV:LV = 1.4. Gena dorsally well developed.

Mesosoma. Scutum without distinct anterior impression; scutum and scutellum densely punctate, punctures well defined, most punctures less than half diameter apart, interspaces distinct, slightly shiny to shiny, setae less than 1.0 × MOD long. Mesopleuron distinctly punctate anteriorly, posteriorly impunctate, interspaces coarsely microsculptured, dull; hypoepimeral area coarsely microsculptured, without distinct punctures, dull; ventral part of mesopleuron with ill-defined punctures, interspaces finely microsculptured, slightly shiny. Propodeal dorsum coarsely irregularly sculptured medially, with few short longitudinal ridges basally, uniformly microsculptured, slightly shiny area developed posteromedially; propodeal side uniformly coarsely microsculptured, without longitudinal ridges, slightly shiny; posteromedial margin of dorsum inconspicuously elevated, marginal ridges inconspicuous, slightly ventromedially directed toward groove. Legs densely punctate, punctures small; forebasitarsal rake reddish, with two apical spines, one preapical spine, and two additional spines. Wings slightly infumate with brown veins.

Metasoma. Terga I-III with slightly developed but distinct silvery apical fasciae. Apical depressions of all terga well developed, very sparsely micropunctate. Terga I-III sparsely micropunctate, punctures ill-defined but distinct, interspaces microsculptured, slightly shiny; sculpture of tergum IV-V distinctly coarser than on previous terga, also slightly more densely punctate. Pygidium sparsely punctate, punctures variable, some ill defined, some well defined, but shallow, interspaces microsculptured medially, unsculptured laterally, slightly shiny to shiny. Central part of sternum II with several larger punctures, interspaces microsculptured, shiny; lateral part slightly shiny, densely micropunctate; remaining sterna with uniform sculpture similar to that on sternum II, but more or less reduced laterally.

Coloration. Central part of mandibles, distal tarsomeres, terga and sterna I-III red. Tegulae brown translucent. Apical parts of terga I-III slightly translucent. Remaining body parts black.

##### Geographic distribution.

Known only from Czech Republic.

##### Name derivation.

The species is named after the Bohemian part of the Czech Republic, where the type specimens were collected.

#### Tachysphex cretensis sp. n.

Taxon classificationAnimaliaHymenopteraCrabronidae

http://zoobank.org/873850DC-225A-4948-B691-B76066553CA6

Figs 3 , 44 , 94 , 104

##### Type material.

Holotype : ♂, Greece, Crete: “GR,CRETE – SE / Kato Simi, ~ 1000m / 35°02'N,27°29'E / Sauša lg.,6.-12.V.03”. Holotype in NMPC. Paratype: 1 ♂, same data as holotype.

##### Diagnosis.

*Tachysphex cretensis* sp. n. is most similar to *Tachysphex pompiliformis* and *Tachysphex opacus*. They share a coarsely sculptured mesopleuron and the shape of the mandible is as in the latter species. The species is well recognizable by a coarsely sculptured dark body, four metasomal segments with silvery apical fasciae, the clypeal lip produced medially, the mandibular inner margin shallowly emarginated distally from the inner tooth, lacking any furrow, and a densely microsculptured forefemoral notch. The new species is probably endemic to the island of Crete, Greece.

##### Description of male (holotype).

Body length: 8.1 mm.

Head. Mandibular inner margin shallowly emarginated distally from inner tooth, with no furrow. Clypeus uniformly convex, slightly elevated, top at clypeal midlength; basomedian area large, sparsely punctate, with nearly impunctate line medially; bevel convex, decreasing to clypeal lip, shiny, with several larger punctures; lip distinctly sinuate, conspicuously produced medially, with distinct lateral corner, lip separated from bevel by punctate groove; WML:LCL = 1.2, WCL:WML = 2.4. Supraclypeal area concave, sparsely punctate, punctures ill-defined, interspaces microsculptured, dull. Supraantennal tubercle distinctly elevated on inner side, shiny. Antenna relatively short, LA3:WA3 = 1.5, LA5:WA5 = 2.1. Frons uniformly finely punctate, punctures well defined, less than one diameter apart, interspaces variable in size, shiny; frontal median line distinct, narrow and finely impressed. Vertex finely and uniformly punctate, punctures well defined, one to less than one diameter apart, interspaces slightly microsculptured, slightly shiny. Vertex setae short, semierect medially, but nearly erect close to inner eye margin, less than 1 × MOD, postocellar impression distinct, shallow, punctate like rest of vertex, Y-shaped; vertex moderately wider than long; WV:LV = 1.4. Gena dorsally well developed.

Mesosoma. Scutum without anterior impression; scutum and scutellum densely punctate, punctures well defined, most punctures less than half diameter apart, interspaces distinct, microsculptured, slightly shiny, setae about 1.0 × MOD or less. Mesopleuron rugose to densely punctate, most punctures in ventral half well defined, interspaces coarsely microsculptured to rugose, slightly shiny to dull; hypoepimeral area coarsely rugose, without distinct punctures, with irregular longitudinal ridges, dull; ventral part of mesopleuron with punctures large, relatively well defined, interspaces finely microsculptured, slightly shiny. Propodeal dorsum coarsely sculptured, with longitudinal ridges; side longitudinally ridged, ridges well developed, interspaces microsculptured, slightly shiny; posteromedial margin of dorsum insignificantly elevated, marginal ridges evanescent, directed ventromedially. Legs densely punctate, punctures small; forefemoral notch small but relatively deep, semicircular, shorter than distance that separates it from forefemoral base, proximal margin relatively sharp, but not distinctly elevated over distal margin; central part of notch slightly elevated, anterior and posterior margin lined by faint ledge, notch surface without distinct setae, coarsely microsculptured, dull. Wings moderately infumate, with brown veins.

Metasoma. Silvery apical faciae of terga I-IV distinctly developed. Apical depressions of all terga shallow, nearly as densely micropunctate as more anterior parts. Terga I-III densely and distinctly micropunctate, punctures ill defined, interspaces microsculptured, slightly shiny; sculpture of terga IV-VII coarser than on previous terga, also puncture denser. Sterna uniformly punctate, nearly like terga. Volsella brown, ventral setae pointing in various directions; apex with numerous setae; dorsal process about as wide as corpus.

Coloration. All body black, except central part of mandibles and terminal tarsomeres dark red. Tegulae brown translucent. Apical parts of all terga slightly translucent.

##### Geographic distribution.

Known only from the island of Crete, Greece.

##### Note.

Female unknown. Only two males have been collected so far.

##### Name derivation.

The species is named after the island of Crete, Greece, where the specimens were collected.

#### Tachysphex dimidiatus

Taxon classificationAnimaliaHymenopteraCrabronidae

(Panzer, 1809), restored from synonymy

Figs 4 , 16 , 22 , 24 , 30 , 56 , 60 , 64 , 71 , 83 , 95 , 107

Larra dimidiata Panzer, 1809 (Heft 106: pl. 13), ♀. Type material lost. Type was probably from Germany.

##### Type material.

Neotype: ♀, Germany: “Germany, BY, N of / Nürnberg, Tennenloher / Forst, 49,57'N 11.04 / leg. Schmid-Egger / 22.6.2008 by-te // Tachysphex / pompiliformis Panzer ♀ / det Schmid-Egger 2010 // ZSM / HYM 04855”. Neotype in ZSM, present designation.

##### Additional material examined.

**Austria**: Kapfenstein env., Steirmark, 1 ♂, 12. vi. 1989, J. Tiefenthaler lgt., OLML; Lechaue, Forchbach 1 km W, Tirol, A-lech3, 1 ♂, 23. vi. 2006, Ch. Schmid-Egger lgt., ZSM HYM 04856; Lechufer, Unter-Pinswang, Reutte 6km NW, Tirol, A-lech1, 1 ♂, 23. vi. 2006, Ch. Schmid-Egger lgt., ZSM HYM 04857; Mönchgraben, Ebelsberg, Oberösterreich, 1 ♀, 22. viii. 1948, F. Koller lgt., ZSM; **Czech Republic**: Boh. centr., Cihelna v Bažantnici, Praha-Hloubětín, 1 ♂, 2 ♀♀, 7. vii. 1998, 1 ♂, 5 ♀♀, 17. viii. 1998, 5♂♂, 2 ♀♀, 22. vi. 1998, 7 ♂♂, 3 ♀♀, 2. viii. 1998, J. Straka lgt., JSPC; Divoká Šárka, Praha-Liboc, 1 ♀, 16. viii. 1998, 1 ♂, 10. vii. 1998, 1 ♂, 4. viii. 1998, J. Straka lgt., JSPC; Chvalské skály, Praha-H. Počernice, 1 ♀, 18. viii. 1998, J. Straka lgt., JSPC; Chvalský lom, Praha-H. Počernice, 1 ♀, 3. vi. 1998, 1 ♂, 1 ♀, 28. vi. 1998, 5 ♂♂, 1 ♀, 3. viii. 1998, J. Straka lgt., JSPC; Podbabské skály, Praha-Dejvice, 1 ♀, 5. vii. 2003, J. Straka lgt., JSPC; Radotínské údolí, Praha-Radotín, 1 ♀, 15. viii. 1998, J. Straka lgt., JSPC; Boh.mer., Chlum u Třeboně, lom Františkov, 1 ♂, 1 ♀, 29. vii. 2002, J. Halada lgt., JSPC; Boh.or., Bartoušov, sand quarry, 1 ♂, 28.-31. vii. 2015, J. Straka lgt., JSPC; Veská, Pardubice env., sand dune, 1 ♂, 1. vi. 2012, R. Tropek lgt., JSPC; Mor. mer., Mutěnice, 1 ♀, 12. viii. 1963, J. Strejček lgt., CSE; Velká Klajdovka NR, Brno-Líšeň, 3 ♂♂, 11. vi. 2014, M. Říha lgt., MRBC, JSPC; Mor. or., Krhová, clay quarry, natural reclamation, 1 ♀, 3.-5. viii. 2015, J. Straka lgt., CSE; Kurovice, lom, vápenec, přírodní rekultivace, 1 ♂, 3.-5. viii. 2015, J. Straka lgt., CSE; Rasová, sandstone quarry, 1 ♀, 4.-6. viii. 2015, J. Straka lgt., CSE; Žlutava, sandstone quarry, 1 ♀, 4.-6. viii. 2015, J. Straka lgt., JSPC; **Bulgaria**: Banitsa, 1 ♀, 31. vii. 2013, L. Toshkov lgt., IBER; Gorna Ribnitsa S, Maleshevska Planina, 1 ♂, 22. vi. 2007, T. Ljubomirov lgt., IBER; Gorno Kadiysko, Etropolska Planina, 4 ♂♂, 19. viii. 2008, T. Ljubomirov lgt. IBER; Gotsev Vruh W, Slavyanka Mts., 1 ♀, 2. ix. 2006, N. Simonov & M. L. Langourov lgt., IBER; Kameshtitsa reserve, 1 ♂, 30. vi. 2014, T. Ljubomirov lgt., IBER; Macedonia cotage W, Rila Mts., 1 ♂, IBER, CCDB-05716-H01; 1 ♂, CCDB-05716-H02, 1 ♂, CCDB-05716-H04, 28. viii. 2010, 1♂, 1 ♀, 31. vii. 2009, T. Ljubomirov lgt., IBER; Parangalitsa NW, Rila Mts., 2 ♀♀, 6. vii. 2007, 1 ♀, 27. viii. 2010, 1 ♂, 29. viii. 2010, 1 ♂, 12. vii. 2011, 1 ♂, 27. viii. 2011, 1 ♂, CCDB-05716-H10, 29. viii. 2011, 2 ♀♀, 18. vii. 2012, T. Ljubomirov lgt., IBER; Razdol, Maleshevska Planina, 6 ♂♂, 3 ♀♀, 27. vi. 2008, T. Ljubomirov lgt., IBER;Vodni Pad SE, 1 ♂, 18. viii. 2013, O. Todorov lgt., IBER; Vrah pek, Ilyov SE, Maleshevska Planina, 2 ♂♂, 1 ♀, 28. vi. 2008, T. Ljubomirov lgt., IBER; Chiša Kamenica, Pirin Mts., 4 ♂♂, 3 ♀♀, 11. viii. 2005, J. Straka lgt., JSPC; **France**: Valvignères, “Les Reynauds” Weinberge, 15 km SW Montélimar, 1 ♀, 18. vi. 2004, J. v. d. Smissen lgt., USMB; **Germany**: Breitbrunn/Ammersee, 1 ♂, 14. iv. 2005, Ch. Schmid-Egger lgt., CSE; Bretten, Diefenbach, Stromberg, Baden-Württemberg, S15, 1 ♂, 8. viii. 1990, Ch. Schmid-Egger lgt., CSE; Grafenau, 6km N, Bayern, Rie-1, 1 ♀, 23. vii. 2008, Ch. Schmid-Egger lgt., ZSM HYM 04854; Garmisch-Partenkirchen, Wiese E Vogelwarte, 1 ♀, 10. v. 2012, D. Doczkal lgt., ZSM, Lagerlechfeld 4 km NE, Bayern, 1 ♂, 5. viii. 2007, Ch. Schmid-Egger lgt., CSE; Mühltal, Starnberg, 1 ♂, 1♀, 6. vi. 1948, F. Stöcklein lgt., ZSM; Ochsenbach, Stromberg, Vaihingen/Enz, Baden-Württemberg, Ob5, 1 ♂, 8. viii. 1990, Ch. Schmid-Egger lgt., CSE; Starnberg, 1 ♂, 1 ♀, 16. vii. 1939, F. Stöcklein lgt., ZSM; Waldtruderinger Forst, Neuperlach, München, 1 ♂, 9. vi. 1998, J. Schuberth lgt., ZSM; **Italy**: Eita, Bormio 14 km SW, Valtellina, Lombardia prov.; 1 ♂, 9. vii. 2006, Ch. Schmid-Egger lgt., CSE; Lasa, Vinschgau 2 km N, Trentino-Alto Adige prov., 3 ♂♂, 8. vi. 2007, Ch. Schmid-Egger lgt., CSE; Lillaz, Valle d’Aosta, 14 km S Aosta, I-aoF, 1 ♂, 7. vii. 1995, Ch. Schmid-Egger lgt., CSE; Passo Gavia 3 km N, Bormio 13 km SE, Lombardia prov., 5 ♀♀, 27. vii. 2007, Ch. Schmid-Egger lgt., CSE; Pondel, Aosta 8 km SW, Valle d’Aosta prov., 1 ♀, 4. viii. 1996, Ch. Schmid-Egger lgt., CSE; Tubre/Taufers, Vinschgau 1 km NE, Trentino-Alto Adige prov., 2 ♂♂, 1 ♀, 27. vii. 2007, Ch. Schmid-Egger lgt., CSE; Vetan, Aosta 9 km W, Valle d’Aosta prov., 1 ♂, 15. vi. 1996, P. Rosa lgt., CSE; Lanzada, Sondrio 11 km N, Lombardia, I-valD, 1 ♀, 9. vii. 2006, Ch. Schmid-Egger, ZSM HYM 11876; Partschins, Province of Bolzano - South Tyrol, 1 ♂, 20. vii. 1966, collector not indicated, ZSM; **Sweden**: Harghult, Ökna, 1 ♀, 25. v. 2009, Torrbacke lgt., NJHC; Persö, Eksjö, Småland env., 1 ♀, 7. vi. 2006, N. E. Johansson lgt., NJHC; **Switzerland**: Dräi, Centovalli, Tessin prov., 1 ♀, 22. viii. 2004, W. Schlaefle lgt., WSKC; Guarda, Engadin, Grisons prov., 1 ♂, 26. vii. 2007, W. Schlaefle lgt., WSKC; Chandolin/Siders, Wallis prov., 1 ♀, 18. vii. 1989, Perraudin lgt., CSE; La Punt, Engadin, Grisons prov., 1 ♂, 3 ♀♀, 25. vii. 2007, W. Schlaefle lgt., WSKC; La Punt, Engadin, Grisons prov., 3 ♂♂, 2 ♀♀, 21. vii. 2004, W. Schlaefle lgt., WSKC; Maggiadelta, 1 ♀, 15. vii. 2005, W. Schlaefle lgt., WSKC; Onsernonetal, 1 ♂, 1 ♀, 1. viii. 2003, W. Schlaefle lgt., WSKC. Specimens determined as *Tachysphex dimidiatus* aff.: **Bulgaria**: Kazanite E, Pirin Mts., 1 ♂, 31. viii. 2006, N. Simonov & M. L. Langourov lgt., IBER; Rozovo N, 1 ♂, 5. vii. 2012, T. Ljubomirov lgt., IBER; **France**: Col Chioula, Ax les Thermes, Pyrénées Mts., 1 ♀, 15. ix. 1987, Ch. Schmid-Egger lgt., CSE; Porte Puymorens, Pyrinees Mts., 1 ♀, 15. ix. 1987, Ch. Schmid-Egger lgt., CSE; Preste, Pic du Canigou, Pyrinees Mts., 3 ♀♀, 13. ix. 1987, Ch. Schmid-Egger lgt., CSE; **Greece**: Ano Trikala, Mt. Killini, Hellas 3 ♂♂, 30. v. 1995, 3 ♂♂, 23. vi. 1996, W. Arens lgt., WAPC; Meg. Tourla, Oros Parnon, Hellas, 1 ♂, 7. vii. 2007, W. Arens lgt., WAPC; Michas, Erymanthos, Achaea, 1 ♂, 23. vi. 1995, 3 ♂♂, 3 ♀♀, 10. vii. 1996, 3 ♂♂, 12. vi. 1997, 3 ♀♀, 27. vii. 1997, W. Arens lgt., WAPC; Oros Chelmos, Xerokambos, Hellas, 5 ♂♂, 3 ♀♀, 10. vi. 1997, W. Arens lgt., WAPC; Panahaikon Mts., Patras env., Achaea, 1 ♀, 4. vii. 2001, W. Arens lgt., WAPC; Panahaikon Mts., Patras env., Achaea, 1 ♂, 24. vi. 1995, 3 ♂♂, 1 ♀♀, 11. vii. 1996, W. Arens lgt., WAPC; Toriza-Prof. Ilias, Oros Taygetos, Hellas, 1 ♂, 8. vii. 1997, W. Arens lgt., WAPC; **Morocco**: Algou NE, 1 ♀, 22. v. 2013, T. Ljubomirov lgt., IBER; **Spain**: Ripoll 27km NE, Pirineos Orient, 2 ♂♂, 1. viii. 2011, J. Halada lgt., JSPC, ZSM HYM24312.

##### Diagnosis.

*Tachysphex dimidiatus* (Panzer) is difficult to distinguish from *Tachysphex jokischianus* (Panzer), *Tachysphex pompiliformis* (Panzer) and *Tachysphex punctipleuris* sp. n. It is variable in most characters, and not a single character consistently differentiates it from the other species. It has the following combination of characters: ♀: The clypeus is markedly convex, the most elevated point is located between the clypeal midlength and the basal third. The clypeal bevel is distinctly triangular, it reaches the base of the clypeus in the middle, and separates the basomedian area into two parts; when the base of the clypeus is punctate, the punctures are larger and the distances between them are distinctly larger than on the lateral side of the basomedian area. The clypeal lip is arcuate or straight, without the medial anterior emargination even in fresh specimens. The vertex setae are semierect medially and nearly erect and slightly shorter than the MOD close to inner eye margin. The head is transversally oval in front view, with irregularly punctate frons. The gena is robust. The dorsolateral cuticular projection of the propodeal spiracle is slightly arcuate to nearly straight, with the apex reddish transparent. The posteromedial margin of the propodeal dorsum is elevated and produced between the marginal ridges, the marginal ridges directed ventromedially toward the groove on the posterior surface. The propodeal side is longitudinally ridged; ridges anteriorly inconspicuous or absent. The propodeal dorsum often has inconspicuous, irregular longitudinal ridges. The punctures of the mesopleuron are exceptionally variable but usually small and inconspicuous. The terga are sparsely punctate, the punctures are ill defined. Apical depressions of tergum V densely microsculptured with micropunctures, densely punctate latereally. The pygidium is of usual size and distinctly microsculptured in most specimens. ♂: The clypeus is convex, conspicuously elevated in the basal half, continuously declining apically, the top at the clypeal midlength or in the basal half of the clypeus. The clypeal bevel is well developed, shiny. The gena is robust. The supraclypeal area is flat, the supraantennal tubercles are connected, slightly elevated ventromedially. The mesopleuron is distinctly punctate laterally and ventrally, the punctures are ill defined. When the interspaces between the punctures are distinct, then they are microsculptured, slightly shiny to dull. The forefemoral notch is relatively shallow, as long as the distance that separates it from the forefemoral base, central part slightly elevated and lined by a ledge on the anterior as well as the posterior margin. The propodeal side is variable but without basal ridges in most specimens. The posteromedial margin of the propodeal dorsum is elevated and produced between marginal ridges, which are directed ventromedially toward the groove on the posterior surface. The terga are sparsely micropunctate with variable interspaces between punctures.

##### Description of male.

Body length: 5.0–8.0 mm.

Head. Mandibular inner margin with one rectangular tooth and distinct furrow next to tooth distally. Labrum flat. Clypeus convex, conspicuously elevated in basal half and continuously declining apically, top at clypeal midlength or in basal half of clypeus; basomedian area large, densely punctate, sparsely punctate medially in some specimens; bevel slightly convex to nearly flat, variable in size, shiny with several larger punctures; lip slightly arcuate, short, with small lateral incisions, lip separated from bevel by variable fine groove; WML:LCL = 1.3–1.5, WCL:WML = 2.2–2.3. Supraclypeal area flat, distinctly punctate, interspaces between punctures shiny to dull. Supraantennal tubercle small, slightly elevated on inner side. Antenna relatively short, LA3:WA3 = 1.5–1.6, LA5:WA5 = 1.9–2.1. Frons uniformly punctate, punctures well defined, less than one diameter apart, interspaces variable in size; frontal median line distinct, narrow, finely impressed. Vertex punctate, punctures well defined, less than half to one diameter apart, interspaces slightly microsculptured, shiny to slightly shiny. Vertex setae short, semierect medially, but nearly erect close to inner eye margin, less than 1 × MOD, postocellar impression distinct, shallow, open widely, V to Y-shaped; vertex moderately wider than long; WV:LV = 1.5-1.6. Gena dorsally well developed.

Mesosoma. Scutum without distinct anterior impression; scutum and scutellum densely punctate, punctures well defined, most punctures less than one diameter apart, interspaces distinct, unsculptured, shiny, setae about 1.0 × MOD. Mesopleuron coarsely microsculptured, with or without distinct punctures; hypoepimeral area irregularly microsculptured, impunctate; ventral part of mesopleuron with punctures ill defined, but deep in some specimens, less than one diameter apart, interspaces shiny. Propodeal dorsum relatively finely, irregularly rugose, with irregular longitudinal ridges basally in some specimens; propodeal side irregularly longitudinally ridged, ridges variable, usually faint, or absent basally, microsculptured, dull; posteromedial margin of propodeal dorsum elevated and produced between marginal ridges, marginal ridges directed ventromedially toward groove on posterior surface. Legs densely punctate, punctures small; forefemoral notch relatively shallow, as long as distance that separates it from forefemoral base to proximal margin of notch, central part slightly elevated and lined by ledge on anterior as well as posterior margin, surface without distinct setae, microsculptured, dull. Wings slightly infumate with brown veins.

Metasoma. Terga I-III with silvery apical faciae. Apical depressions of all terga shallow, micropunctate. Terga I-III sparsely, finely micropunctate, punctures evanescent in microsculpture, slightly shiny; sculpture of tergum IV-VII coarser, than on previous terga, also punctation slightly denser. Sterna uniformly punctate nearly like terga. Volsella light brown, ventral setae pointing in various directions; dorsal process slightly wider than corpus in most specimens.

Coloration. Apical part of mandibles, tarsi, forefemoral notch, tegulae, terga and sterna I, II, and partly or all tergum III red. Apical parts of all terga and tegula translucent. Remaining body parts all black. Forefemoral notch very rarely black in specimens from high altitude mountains.

##### Description of female

(neotype). Body length: 7.4 mm.

Head. Clypeus distinctly convex, top at clypeal midlength; basomedian area relatively large, densely punctate as lateral section; bevel large, distinctly convex, triangular, with sparse, large punctures, reaching base of clypeus, shiny; lip slightly arcuate, with lateral incisions, separate from bevel by punctate groove, WML:LCL = 1.7, WCL:WML = 1.8. Supraclypeal area flat, distinctly punctate, but shape of punctures ill defined, interspaces between punctures shiny to slightly shiny. Supraantennal tubercle small, slightly elevated on inner side. Frons uniformly punctate, punctures well defined, less than one diameter apart, interspaces variable in size; frontal median line distinct, narrow, well impressed. Vertex punctate, punctures well defined, less than half to one diameter apart, interspaces unsculptured, shiny. Vertex setae short, semierect medially, nearly erect close to inner eye margin, distinctly shorter than 1 × MOD; postocellar impression distinct, shallow, open, widely Y-shaped; vertex moderately wider than long; WV:LV = 1.5. Gena dorsally well developed.

Mesosoma. Scutum without distinct anterior impression; scutum and scutellum densely punctate, punctures well defined, half to one diameter apart, interspaces slightly microsculptured, shiny, setae about 1.0 × MOD. Mesopleuron coarsely microsculptured, without distinct punctures; hypoepimeral area irregularly microsculptured, impunctate; ventral part of mesopleuron with punctures ill defined, less than one diameter apart, interspaces shiny. Propodeal dorsum relatively finely, irregularly rugose, with few incomplete irregular, longitudinal ridges; propodeal side irregularly and incompletely longitudinally ridged, ridges absent basally, microsculptured, dull; posteromedial margin of propodeal dorsum elevated and slightly produced between marginal ridges which are directed ventromedially toward groove on posterior surface. Legs densely punctate, punctures small; forebasitarsal rake reddish, with three apical spines, one preapical spine, and two additional spines. Wings slightly infumate with brown veins.

Metasoma. Terga I-III with silvery apical fasciae. Apical depressions of all terga shallow, with micropunctures evanescent in microsculpture. Terga I-III sparsely and finely micropunctate, punctures ill defined, interspaces microsculptured, shiny to slightly shiny; sculpture of tergum IV-V slightly coarser than on previous terga, also slightly more densely punctate. Pygidium sparsely punctate, punctures ill defined, interspaces microsculptured, slightly shiny. Central part of sternum II with several larger punctures, interspaces microsculptured, shiny; lateral part slightly shiny, densely micropunctate; remaining sterna with uniform sculpture similar to that on sternum II, but sculpture more or less reduced laterally.

Coloration. Central part of mandibles, three distal tarsomeres, terga and sterna I-III red. Tegulae brown translucent. Apical parts of terga I-III slightly translucent. Remaining body parts all black.

Variation of females. Extremely variable in most of characters. The clypeus is typically conspicuously elevated basally, with the top in the basal third; the bevel can be well separated from the clypeal base in some specimens, althouth infrequently. Vertex wider than long in large specimen and slightly wider than long in small specimens, WV:LV = 1.2–1.5. Antenna short, LA3:WA3 = 2.2–2.4, LA5:WA5 = 2.6–2.7.

##### Geographic distribution.

Austria, Czech Republic, Bulgaria, France, Germany, Italy, Sweden and Switzerland. Specimens from mountains of Greece, Morocco and Spain probably also belong to this species.

#### Tachysphex ferrugineus

Taxon classificationAnimaliaHymenopteraCrabronidae

Pulawski, 1967

Figs 96 , 105

##### Type material.

Holotypus: ♂, Turkey: Trabzon: Boztepe, 18.v.1962, K. M. Guichard and D. H. Harvey leg. Holotype in BMNH, examined in 2003.

##### Additional material examined.

**Georgia**: Sairme, Caucasus, 1 ♀, 20. vii. 1983, Kadlec & Voříšek lgt., PTLC; **Russia**: Teberda, Karachay-Cherkessia, 1 ♀, 9. vi. 1978, W. J. Pulawski lgt., ZIN; **Turkey**: Trabzon: same data as holotype, 3 ♂♂, 1 ♀; Damar env., Artvin prov., 1 ♂, 1 ♀, 2. vii. 1997, P. Průdek & M. Říha lgt., JSPC; Samsun University campus, Samsun prov., 2 ♂♂, 4. vii. 2014, J. Barták & Kubík lgt., JSPC.

##### Diagnosis.

*Tachysphex ferrugineus* Pulawski is easily recognizable by the body coloration, but in morphological characters and body sculpture it resembles *Tachysphex dimidiatus* (Panzer), *Tachysphex jokischianus* (Panzer), *Tachysphex pompiliformis* (Panzer) and *Tachysphex punctipleuris* sp. n. The species is similar to *Tachysphex stysi* Straka from central Asia in coloration, but *Tachysphex stysi* belongs to the *Tachysphex austriacus* species subgroup. *Tachysphex ferrugineus* Pulawski is largely light red colored. All tibiae, tarsi and nearly all metasoma, except basal part of terga IV-VI in males and IV-V in females, are light red. The species is similar to *Tachysphex dimidiatus* in body morphology and sculpture but differs in the following characters: ♀: Supraantennal tubercle distinctly elevated, but supraclypeal area nearly flat. Gena inconspicuously developed but not short. Pygidium densely punctate, all coarsely microsculptured, dull. ♂: Gena dorsally inconspicuous. Vertex finely and densely punctate, and postocellar impression shallow. Forefemoral notch large, usually longer than the space that separates it from forefemoral base.

##### Geographic distribution.

Georgia, Russia (Caucasus) and Turkey.

##### Note.

Full description of the species is presented by Pulawski (1971).

#### Tachysphex jokischianus

Taxon classificationAnimaliaHymenopteraCrabronidae

(Panzer, 1809), restored from synonymy

Figs 5 , 17 , 23 , 31 , 39 , 45 , 51 , 57 , 61 , 65 , 72 , 84 , 88 , 97

Larra Jokischiana Panzer, 1809 (Heft 106: pl. 15), ♀.

##### Type material.

Holotype: ♀, no specific locality at the label but probably from Germany. Holotype by monotypy. “Coll. Sturm [printed] // Ca. // Larra / Jokischi / ana / Panz. [handwriting, card with black frame] // 54 [printed, red card] // Larra / Jokischiana / Panz. Typus / J. de Beaumont / det. 1955 [handwriting on pre-printed card with name of determinator and year] // Tachysphex ♀ / pompiliformis Pz. / J. de Beaumont / det. 1955 [handwriting on pre-printed card with name of determinator and year] // Typus Nr. / Zoologische / Staatsamlung / München. [printed, red card]”. Holotype in ZSM, examined.

##### Additional material examined.

**Austria**: Seefeld NW, Niederösterreich, 1 ♂, 19. v. 2015, M. Halada lgt., OLML; **Czech Republic**: Boh. bor., Počerady, ash coal deposit from power station, 1 ♂, 23. v. 2010, 1 ♂, 7. vi. 2010, 1 ♂, 1 ♀, 27. vi. 2010, 1 ♂, 1 ♀, 1. viii. 2010, R. Tropek lgt., JSPC; Boh. centr., Cihelna v Bažantnici, Praha-Hloubětín, 14 ♂♂, 1 ♀, 28. v. 1998, 6♂♂, 1 ♀, 22. vi. 1998, J. Straka lgt., JSPC; Černošice, 1 ♂, 30. v. 1968, Z. Pádr lgt., OLML; Divoká Šárka, Praha-Liboc, 1 ♂, 29. v. 1998, J. Straka lgt., JSPC; Heřmanův Městec, sandpit, 2 ♂♂, 1 ♀, 1. vii. 2012, 1 ♀, 1. viii. 2012, R. Tropek lgt., JSPC; Hrachovka, Praha-Troja, 1 ♀, 28. vi. 1998, J. Straka lgt., JSPC; Chvaletice, ash coal deposit, 1 ♂, 1. viii. 2012, R. Tropek lgt., JSPC; Chvalská navážka, Praha-H. Počernice, 1 ♂, 9. vii. 1997, 1 ♀, 22. viii. 1997, 2 ♂♂, 1 ♀, 6. vii. 1998, 1 ♀, 20. viii. 1998, J. Straka lgt., JSPC; Chvalský lom, Praha-H. Počernice, 1 ♀, 6. vii. 1998, J. Straka lgt., JSPC; Jenštejn, 4 ♂♂, 22. vi. 2001, Jos. Straka lgt., JSPC; Klánovický les, Praha-Klánovice, 1 ♂, 20. vi. 1998, J. Straka lgt., JSPC; Opatovice, ash coal deposit, 1 ♀, 1. vi. 2014, R. Tropek lgt., JSPC; Písečný vršek, Praha-Běchovice, 1 ♀, 2. vii. 1997, 5 ♂♂, 1 ♀, 6. vii. 1998, M. Kolísko & J. Straka lgt., JSPC; Písty, Nymburk env., sand dune, 1 ♀, 1. vii. 2012, 1 ♀, 1. viii. 2012, R. Tropek lgt., JSPC; Travčický les, Terezín env., 1 ♂, 6. viii. 1981, Z. Pádr lgt., OLML; Valy, 1 ♂, 7. viii. 1988, Odehnal lgt., OLML; Boh. mer., Blatná, sand quarry, 1 ♂, 1998, P. Bogusch lgt., JSPC; Cep 1, sand quarry, 1 ♂, 4.-9. vi. 2013, 1 ♀, 1.-5. vii. 2013, R. Tropek, I. Černá, O. Čížek lgt., JSPC; Františkov, Chlum u Třeboně, 1 ♂, 29. vii. 2002, J. Halada lgt., JSPC; Hodějovice, 5 ♂♂, 1 ♀, 3. vi. 2015, 1 ♀, 2. viii. 2013, R. Tropek, I. Černá, O. Čížek lgt., JSPC; Plavsko, 1 ♂, 5. vii. 2013, R. Tropek, I. Černá, O. Čížek lgt., JSPC; Těšínov, 1 ♀, 2. viii. 2013, R. Tropek, I. Černá, O. Čížek lgt., JSPC; Slepičí vršek, Třeboňsko PLA, 1 ♂, 2 ♀♀, 6. viii. 1997, J. Straka lgt., JSPC; Vlkov, Třeboňsko PLA, 1 ♂, 6. viii. 1997, J. Straka lgt., 1 ♂, 1 ♀, 9. vi. 2013, R. Tropek, I. Černá, O. Čížek lgt., JSPC; Boh.occ., Starý lom, ash coal deposit from Prunéřov power station, 1 ♀, 26. vi. 2010, 1 ♀, 31. vii. 2010, R. Tropek lgt., JSPC; Tušimice, ash coal deposit from power station, 1 ♂, 6. vi. 2010, 2 ♂♂, 2 ♀♀, 26. vi. 2010, 1 ♀, 22. viii. 2010, R. Tropek lgt., JSPC; Boh. or., Plachta NR, Hradec Králové, 2 ♂♂, 1. vii. 1998, J. Straka lgt., JSPC; Veská, Pardubice env., sand dune, 1 ♂, 20. v. 2007, 1 ♀, 27. v. 2007, P. Bogusch lgt., 1 ♀, 1. viii. 2012, 3 ♂♂, 1 ♀, 1. vii. 2012, R. Tropek lgt., JSPC; Mor.mer., Bzenec env., 1 ♂, 18. vi. 1942, A. Hoffer lgt., 1 ♀, viii. 1963, M. Kocourek lgt., OLML; Bzenec env., Vojenské cvičiště NR, 1 ♀, 8. vi. 1997, M. Kolísko lgt., 1 ♀, 4. viii. 2015, J. Straka lgt., JSPC; Bzenec-přívoz, Bzenec env., 4 ♂♂, 31. v. 2002; J. Straka lgt., JSPC; Čučice 1 km S, 2 ♂♂, 1.-15.vii.2001, M. Říha lgt., MRBC; Dolní Bojanovice, 1 ♂, viii. 1941, A. Hoffer, OLML; Mistřín, 1 ♂, viii. 1942, A. Hoffer lgt., OLML; Pouzdřany, 1 ♂, 19. vi. 1970, J. Strejček lgt., OLML; Rohatec lgt., 1 ♂, vii. 1941, 2 ♀♀, vii. 1942; A. Hoffer, OLML; **France**: Falaise, 1 ♂,date and collector unknown, ZSM; Plateau du Coiron, 16 km NW Montélimar, 2 ♂♂, 24. vi. 2001, 2 ♂♂, 1 ♀, 11.-14 vii. 2002, 2 ♂♂, 1 ♀, 20. vi. 2004, J. v. d. Smissen lgt., USMB; Lachamp - Raphael W, D 122, 23 km NW Aubenas, 1 ♂, 2 ♀♀, 18. vi. 2004, 1 ♀, 29. vii. 2003, J. v. d. Smissen lgt., USMB; Cereste, Apt., Provence prov., 1 ♀, 9. ix. 1987, Ch. Schmid-Egger lgt., CSE; **Germany**: Allersberg, 1 ♂, 17. viii. 1956, Ettinger lgt., ZSM; Blankenförde, Mecklenburg-Vorpommern, mn-hex, 2 ♀, 7. vii. 2013, Ch. Schmid-Egger lgt., ZSM HYM 19879, ZSM HYM 19880; Blankensee, Lübeck, Schleswig-Holstein, 1 ♀, 18. vi. 1989, J. v. d. Smissen lgt., USMB; Brammerau, Nortorf, Schleswig-Holstein, 1 ♂, 1 ♀, 22. vii. 1995, J. v. d. Smissen lgt., USMB; Brünkendorf/Dannenberg, Niedersachsen, 3 ♂♂, 2. viii. 1995, J. v. d. Smissen lgt., USMB; Büchen, Schleswig-Holstein, 10 ♂♂, 10. vii. 1994, J. v. d. Smissen lgt., USMB; Damsdorf, Bornhöved 6km E, Schleswig-Holstein, 1 ♂, 18. vii. 1999, J. v. d. Smissen lgt., USMB; Eichholz, Lübeck, Schleswig-Holstein, 1 ♀, 25. vii. 1989, J. v. d. Smissen lgt., USMB; Friedrichsfeld, Mannheim, Baden-Württenberg, 1 ♂, 22. v. 1989, Ch. Schmid-Egger lgt., CSE; Friedrichsort, Kiel, Lehmgrube, 1 ♀, 1. vii. 1961, collector unknown, ZSM; Gabow, Bad Freienwalde 4km NE, gab, 1 ♀, 25. vi. 2001, Ch. Schmid-Egger lgt., CSE; Gemünda, Sesslach, Bayern, 2 ♂♂, 9. vii. 1994, J. v. d. Smissen lgt., USMB; Grissheim, Baden-Württemberg, gr, 1 ♂, 18. vii. 2012, Ch. Schmid-Egger, ZSM HYM 17628; Grissheim, Rhein, Freiburg 24 km SW, Baden-Württemberg, 1 ♀, 1. vii. 1999, J. v. d. Smissen lgt., USMB; Gudow, Schleswig-Holstein, 1 ♀, 1. ix. 1991, J. v. d. Smissen lgt., USMB; Ingolstadt, 1 ♀, 21. vii. 1948, H. Freude lgt., ZSM; Istein, Baden-Württenberg, 1 ♂, 26. vii. 1989, Ch. Schmid-Egger lgt., CSE; Klein Kühren/Dannenberg, Niedersachsen, 1 ♂, 15. vi. 1996, 1 ♀, 10. viii. 1996, J. v. d. Smissen lgt., USMB; Laasche/Dannenberg, Niedersachsen, 3 ♂♂, 5 ♀♀, 19. vii. 1994, 12 ♂♂, 23. viii. 1994, J. v. d. Smissen lgt., USMB; Lenggries, Isarauen, 1 ♀, 27. vii. 2003, Ch. Schmid-Egger lgt., ZSM; Malk Göhren, Mecklenburg-Vorpommern, 2 ♂♂, 22. vii. 1997, J. v. d. Smissen lgt., USMB; Mallnow, Framkfurt an der Oder 13km NNW, Brandenburg, br-ma, 1 ♀, 22. vii. 2009, ZSM HYM 14826, 1 ♀, 26. vi. 2010, Ch. Schmid-Egger lgt., ZSM HYM 14825; Malsch, 1 ♀, 25. vi. 1967, R. Degen lgt., ZSM; Oldenburg 5km NW, Schleswig-Holstein, 1 ♀, 26. vii. 1997, 2 ♀♀, 4. viii. 1997, J. v. d. Smissen lgt., USMB; Putlos, Oldenburg, Schleswig-Holstein, 1 ♀, 29. vi. 1997, 1 ♀, 4. viii. 1997, J. v. d. Smissen lgt., USMB; Sandhausen, Heidelberg, Baden-Württenberg, 1 ♂, 20. v. 1989, Ch. Schmid-Egger lgt., CSE; Spreenhagen 4km W, Brandenburg, brsp, 1 ♂, 27. vi. 2011, Ch. Schmid-Egger lgt., ZSM HYM 14827; Steinenstadt, Baden-Württemberg, 1 ♂, 31. vii. 2005, W. Schlaefle lgt., WSKC; Zossen, Brandenburg, zos, 1 ♀, 14. vi. 2011, Ch. Schmid-Egger lgt., ZSM HYM 09867; **Hungary**: Dunatetétlen, saline lake, Kecskemét 45km SEE, 1 ♂, 17. vii. 2013, J. Habermannová, J. Straka lgt., JSPC; Hegyshalom N, 1 ♂, 1 ♀, 6. vi. 2015, M. Halada lgt., OLML; Kecskemét env., 1 ♂, 16. vii. 2006, J. Halada lgt., JSPC; Kesthely env., lime stone, 1 ♂, 1 ♀, 23. v. 2014, A. Astapenková, J. Habermannová, J. Straka lgt., JSPC; Kunpeszér env., sandy hill, 1 ♀, 7. vi. 2013, D. Benda, P. Bogusch & J. Straka lgt., JSPC; Kunszentmiklós env., salt marsh, 3 ♀♀, 16. vii. 2013, J. Habermannová, J. Straka lgt., JSPC; Örkeny, puszta, 5 ♂♂, 7. vi. 2015, 1 ♀, 27. vi. 2015, M. Halada lgt., OLML; Pákozd env., pasture stepe, 4 ♂♂, 18. vii. 2013, J. Habermannová, J. Straka lgt., JSPC, 6 ♂♂, 5 ♀♀, 10. vi. 2015, 7 ♂♂, 11 ♀♀, 25. vi. 2015M. Halada lgt., OLML, 1 ♂, 9. vi. 2013, D. Benda, P. Bogusch & J. Straka lgt., JSPC; Pusztavacs, sand dune, 1 ♂, 8. vi. 2015, M. Halada lgt., OLML; Súr 1km S, loes steppe, 1 ♀, 18. vii. 2013, J. Habermannová, J. Straka lgt., JSPC; Szenna, Kaposvár env., 1 ♀, 3. ix. 2007, W. Żyła, USMB; Velencei-tó env., steppe, 1 ♀, 9. vi. 2013, D. Benda, P. Bogusch & J. Straka lgt., JSPC; **Poland**: Białowieża forest, 1 ♂, 19. vii. 1995, R. Dobosz lgt., USMB; Borne Sulinowo, Baltic see coast, 1 ♂, 8. vii. 1997, Kaka lgt., USMB; Grabowiec, Małopolska Upland, 1 ♀, 18. vi. 1995, 2 ♂♂, 4 ♀♀, 12. vii. 1996, W. Żyła, USMB; Jastrzębia Góra, Władysławowo, Baltic see coast, 1 ♂, 16. vi. 1996, 1 ♀, 24. vi. 1996, R. Dobosz lgt., USMB; Kików, Malopolska Upland, 4 ♂♂, 2 ♀♀, 19. vi. 1995, 1 ♀, 14. vii. 1996, W. Żyła lgt., USMB; Kołczewo, Baltic see coast, 1 ♀, 10. vii. 2009, R. Dobosz lgt., USMB; Krzyżanowice, Malopolska Upland, 4 ♂♂, 16. vi. 1995, 1 ♂, 21. vi. 1995, W. Żyła lgt., 1 ♀, 30. vii. 1996, R. Dobosz lgt., USMB; Kuźnica, Baltic see coast, 1 ♂, 1 ♀, 25. vi. 1996, R. Dobosz lgt., USMB; Lubliniec, Upper Silesia, 1 ♂, 12. vi. 1994, R. Dobosz lgt., USMB; Osowo Stare, 1 ♂, 2 ♀♀, 12. viii. 1992, 1 ♂, 28. v. 1993, M. Bunalski lgt., USMB; Pasturka, Pińczów env., Małopolska Upland, 2 ♂♂, 19. vi. 1997, R. Dobosz, W. Żyła lgt., USMB; Ponice, Rabki, Beskid Zachodni, 1 ♀, 17. vii. 1997, W. Żyła lgt., USMB; Przedbórz, Małopolska Upland, 1 ♀, 2. viii. 2007, W. Żyła lgt., USMB; Smołdziński Las, Słowiński NP, 1 ♂, 29. vii. 2004, W. Żyła lgt., USMB; Sycyn Dolny, 2 ♂♂, 2 ♀♀, 22. vi. 1992, 1 ♂, 2 ♀♀, 28. vii. 1992, 1 ♀, 12. viii. 1992, M. Bunalski lgt., USMB; Szombierki, Bytom, 1 ♀, 29. v. 1993, W. Żyła lgt., USMB; Wisła, 1 ♀, 1. viii. 1994, 1 ♂, 8. viii. 1994, W. Żyła lgt., USMB; Władysławowo, 2 ♂♂, 2 ♀♀, 11. vi. 1993, 2 ♂♂, 1 ♀, 13. vi. 1993, 1 ♂,18. vi. 1993, R. Dobosz lgt., USMB; **Slovakia**: Abrod NR, Velké Leváre env., 1 ♀, 6. vii. 2008, J. Straka, P. Janšta, P. Šípek, D. Král lgt., JSPC; Chotín, 2 ♂♂, 3. viii. 1961, 1 ♂, 9. viii. 1961, 1 ♂, 12. vii. 1981, Z. Pádr lgt., 1 ♂, 15. vi. 1977, M. Kocourek lgt., 1 ♀, 26. vii. 1993, M. Halada lgt., OLML; Královský Chlmec, 3 ♂♂, 1 ♀, 25. vii. 1969, Z. Pádr lgt., OLML; Kúty 1 ♂, 9. vi. 1973, J. Strejček lgt., OLML; Lakšárska Nová Ves env., sandy area, 2 ♂♂, 13. vi. 2008, J. Straka lgt., JSPC; Somotor, 2 ♂♂, vii. 1959, M. Kocourek, OLML; Streda nad Bodrogom, 1 ♂, 26. vi. 1977, Z. Pádr lgt., OLML; Štúrovo, 1 ♀, vii. 1957, M. Kocourek lgt., 1 ♂, 24. vi. 1960, 1 ♂, 8. vi. 1964, Z. Pádr lgt., OLML; Velký Kamenec, 1 ♀, 14. vii. 1975, Z. Pádr lgt., OLML; **Sweden**: Dammersryet, Ravlunda, 1 ♂, 9. vii. 2011, N. E. Johansson lgt., NJHC; Gårdby, Öland isl., 1 ♀, 27. vii. 1998, collector unknown, USMB; Havängsdösen, Ravlunda, 1 ♂, 27. vi. 2011, N. E. Johansson lgt., NJHC; Lillesjö, Mörlunda env., 1 ♀, 27. vi. 2009, N. E. Johansson lgt., NJHC; Sandhajd, Fårö env., 2 ♂♂, 1 ♀, 10. vii. 2012, 1 ♀, 12. vii. 2012, N. E. Johansson lgt., NJHC; Sandviken, Gannelgarn env., 1 ♀, 9. vii. 2012, N. E. Johansson lgt., NJHC

##### Diagnosis.

*Tachysphex jokischianus* (Panzer) is difficult to distinguish from *Tachysphex dimidiatus* (Panzer), *Tachysphex pompiliformis* (Panzer) and *Tachysphex punctipleuris* sp. n. It is exceptionally variable in most characters, and no single character differentiates it from the other morphologically similar species. It can be recognized by the following combination of characters: supraclypeal area with distinctly microsculptured interspaces between punctures, usually appearing dull. Gena dorsally robustly built. Mesopleuron, including hypoepimeral area, finely sculptured, punctures in lower parts of mesopleuron distinct but small. Propodeal dorsum usually lacking longitudinal ridges, if present, then only basally. Dorsolateral cuticular projection of propodeal spiracle arcuate to semicircular, with dark apex. Posteromedial margin of propodeal dorsum elevated but not produced between the marginal ridges, marginal ridges positioned nearly horizontally above groove on posterior side. Wings only slightly infumated to pale. ♀: Apical depression of terga IV and V finely microsculptured and impunctate or nearly so, contrasting with coarsely and densely punctate central part of these terga, punctures of terga IV and V well developed. Pygidium often wide, with convex, shiny surface (but not always). Tergum III all red or red laterally and basally, with black area apicomedially. ♂: Surface of supraclypeal area concave, supraantennal tubercle distinctly elevated ventromedially. Forefemoral notch light red, large, its longitudinal diameter usually longer than the space separating it from forefemoral base. Propodeal side densely and finely ridged basally in most specimens. Terga densely micropunctate.

##### Description of female (holotype).

Body length: 7.2 mm.

Head. Clypeus distinctly convex, its top at clypeal midlength; basomedian area relatively large, as densely punctate as lateral section; bevel large, distinctly convex, as long as basomedian area laterally, slightly longer medially, obtusely triangular, with sparse large punctures, not reaching base of clypeus, shiny; lip slightly arcuate, with lateral incision, separated from bevel by punctate groove, WML:LCL = 1.5, WCL:WML = 1.8. Supraclypeal area flat, distinctly punctate, punctures ill-defined, interspaces microsculptured, slightly shiny. Supraantennal tubercle small, slightly elevated on inner side. Both antennae broken off (only three segments present). Frons uniformly punctate, punctures well defined, about half diameter apart or less, most interspaces uniform in size; frontal median line distinct, narrow, finely impressed. Vertex finely punctate, punctures well defined, less than half to one diameter apart, laterally more sparsely punctate than medially, interspaces finely microsculptured, slightly shiny. Vertex setae short, semierect, slightly longer laterally than medially, about 0.5 × MOD; postocellar impression well developed, widely Y-shaped; vertex moderately wider than long; WV:LV = 1.5. Gena dorsally well developed.

Mesosoma. Scutum without distinct anterior impression; scutum and scutellum densely punctate, punctures well defined, less than half to one diameter apart, interspaces well developed, variable in size, microsculptured, slightly shiny, setae about 1.0 × MOD or less. Mesopleuron coarsely microsculptured, without distinct punctures; hypoepimeral area irregularly microsculptured without punctures; ventral part of mesopleuron with punctures ill defined, less than one diameter apart, interspaces small, slightly shiny. Propodeal dorsum relatively finely, regularly rugose, with few indistinct irregular, longitudinal ridges basally, dull, but slightly shiny posteromedially; propodeal side regularly longitudinally ridged, ridges inconspicuous basally, interspaces microsculptured, slightly shiny; posteromedial margin of propodeal dorsum elevated, but not produced between posterior marginal ridges, marginal ridges positioned nearly horizontally above groove on posterior surface. Legs densely punctate, punctures small; forebasitarsal rake light yellow with reddish opalescence at distal half, with three apical spines and two to three additional spines. Wings slightly infumate with brown veins.

Metasoma. Terga I-III with ill defined silvery apical fasciae. Terga I-III densely, finely micropunctate, punctures ill defined, interspaces microsculptured, slightly shiny; apical depressions shallow, with distinct micropunctures; sculpture of terga IV-V distinctly coarser than on previous terga, also slightly more densely punctate; tergum V coarsely punctate, punctures ill-defined, but large, half to one diameter apart, interspaces microsculptured, apical depression of tergum V very sparsely and finely punctate. Pygidium large, sparsely punctate, punctures large, ill defined, interspaces nearly unsculptured, shiny. Central part of sternum II with several larger punctures, interspaces microsculptured, shiny; lateral part slightly shiny, densely micropunctate; remaining sterna with uniform sculpture similar to that on sternum II, but more or less reduced laterally.

Coloration. Central part of mandibles, terga and sterna I-III red. Distal tarsomeres dark red. Tegulae reddish translucent. Apical parts of terga I-III slightly translucent. Remaining body parts black.

Variation of females. Very variable in sculpture, but finely sculptured over all body in most specimens except apical terga (in comparison to other species). Clypeal bevel reaching base of clypeus in some specimens, but well separated from the base in most. Antenna short, LA3:WA3 = 2.2–2.4, LA5:WA5 = 2.6–2.7. Pygidium distinctly microsculptured, of usual width and dull in some specimens, but shiny and wide in most of specimens. Some specimens appear dark in coloration, with only terga I and II dark red and nearly all sternum II black. The most characteristic coloration of metasoma is as follows: segments I and II red, tergum III red basally and laterally, with larger or smaller black area apicomedially and sternum III basally red and apically dark.

##### Description of male.

Body length: 5.6–8.7 mm.

Head. Mandibular inner margin with one distinct rectangular tooth and distinct furrow next to tooth distally. Clypeus slightly convex, uniformly curved, or more steeply declining ventrally than dorsally, top about in clypeal midlength; basomedian area very large, about two thirds of bevel or more, densely punctate; bevel small, slightly convex, shiny with several larger punctures; lip conspicuously arcuate or slightly sinuate, with small or large lateral corner, lip separated from bevel by distinct groove with large punctures; WML:LCL = 1.2–1.5, WCL:WML = 2.2–2.4. Supraclypeal area groove-like, concave, supraantennal tubercle distinctly elevated ventromedially; surface sparsely punctate, punctures ill defined, interspaces densely microsculptured, slightly shiny to dull. Antenna relatively short, LA3:WA3 = 1.5–1.6, LA5:WA5 = 1.9–2.1. Frons finely punctate, punctures well defined, one to less than half diameter apart, interspaces variable in size; frontal median line distinct, narrow, finely impressed. Vertex punctate, punctures well defined, less than one diameter apart, interspaces microsculptured, slightly shiny. Vertex setae short, semierect, less than 1 × MOD; postocellar impression well developed, open, widely Y-shaped; vertex wider than long; WV:LV = 1.4–1.5. Gena dorsally well developed.

Mesosoma. Scutum without distinct anterior impression; scutum and scutellum densely punctate, punctures well defined, most punctures less than half diameter apart, interspaces distinct, microsculptured, slightly shiny to dull, setae about 1.0 × MOD or less. Mesopleuron coarsely microsculptured with distinct or indistinct punctures in lower areas, most punctures ill defined, interspaces dull; hypoepimeral area coarsely microsculptured to finely rugose, dull; ventral part of mesopleuron with small, ill defined punctures, interspaces slightly shiny. Propodeal dorsum variably sculptured, irregularly ridged, areolate, or coarsely irregularly rugose, without distinct longitudinal ridges, short longitudinal ridges developed basally; propodeal side distinctly ridged, more densely and finely ridged basally than in the center in most specimens; posteromedial margin of propodeal dorsum elevated, but not produced between marginal ridges, marginal ridges positioned nearly horizontally above groove on posterior side. Legs densely punctate, punctures small; forefemoral notch light red and large, its diameter usually longer than space that separates it from forefemoral base, central part of notch slightly elevated, anterior and posterior margins lined by small distinct ledge, notch surface without distinct setae, microsculptured, dull. Wings slightly infumate, veins brown.

Metasoma. Terga I-III with silvery, apical fasciae. Apical depressions of all terga distinct, with micropunctures similar as on central part of terga. Terga I-III densely and distinctly micropunctate, punctures ill defined, interspaces microsculptured, slightly shiny; sculpture of terga IV-VII slightly coarser and slightly more densely punctate than other terga. Sterna uniformly punctate nearly like terga.

Coloration. Apical part of mandibles, terga and sterna I and II and forefemoral notch red. Metasoma very variable in coloration; some specimens with only tergum I dark red, or terga I and II and sternum II basally red, or terga and sterna I-III equally red. Distal tarsomeres dark reddish. Tegulae brown to reddish. Apical parts of all terga and tegulae translucent. Remaining body parts black.

##### Geographic distribution.

Austria, France, Germany, Hungary, Poland, Slovakia and Sweden.

#### Tachysphex nigripennis

Taxon classificationAnimaliaHymenopteraCrabronidae

(Spinola, 1808), restored from synonymy

Figs 6 , 18 , 32 , 46 , 66 , 89 , 98 , 103

Tachytes nigripennis Spinola, 1808 (p. 260), ♀. Holotype or syntypes: Italy: “prope Genuam”. Type material lost (de Beaumont 1952, 44).

##### Type material.

Neotypus: ♀, Czech Republic: “CZ, Mor.mer. 30.vii.2008 / sandpit Bzenec-Přívoz I. / Bzenec env.; EtOH 96% [in bold] / Jakub Straka lgt. // ♀ *Tachysphex* / *nigripennis* / Spinola / Jakub Straka det. 2014 // ZSM / HYM 23693”. Neotype in ZSM, present designation.

##### Additional material examined.

**Czech Republic**: Boh. bor., Počerady, ash coal deposit from power station, 1 ♀, 27. vi. 2010, 1 ♀, 1. viii. 2010, R. Tropek lgt., JSPC; Střimická waste dump, Most env., 1 ♀, 30. vii. 2004, P. Tyrner lgt., JSPC; Boh. occ., Starý lom, ash coal deposit from Prunéřov power station, 1 ♂, 26. vi. 2010, R. Tropek lgt., JSPC; Mor. mer., Bratčice env., 1 ♂, 2 ♀♀, 21. vi. 2012, M. Halada lgt., JSPC; Bzenec-Přívoz, Bzenec env., sandpit, 1 ♀, 28. vi. 2012, M. Halada lgt., JSPC; **France**: Collias, W Pont du Gard, 16 km NE Nîmes, 1 ♀, 31. vii. 2003, J. v. d. Smissen lgt., USMB; Dieulefit (D547), 25 km E Montélimar, 1 ♂, 23. v. 2000, J. v. d. Smissen lgt., USMB; Prades 38km SW, Pyrénées Orient., 1 ♂, 22. vii. 2011, J. Halada lgt., JSPC; **Germany**: Speyer, sandy dune, Rhineland-Palatinate prov., 1 ♀, 26. vi. 2008, Reder lgt. et coll.; **Hungary**: Pusztavacs, sand dune, 3 ♂♂, 8. vi. 2015, 1 ♂, 1 ♀, 26. vi. 2015, M. Halada lgt., JSPC, OLML; **Italy**: St. Pierre, Valle d’Aosta prov., 1 ♂, 1. viii. 1997, Ch. Schmid-Egger lgt., CSE; **Kyrgyzstan**: Kashkasu env., Ala-Archa, 1 ♀, vii. 2000, 3 ♂♂, 7 ♀♀, vii. 2002, V. Gurko lgt., OLML; Malinovka, Ala-Archa riv., 1 ♂, 1 ♀, vii. 2000, V. Gurko lgt., JSPC; Ooru-say env., Ala-Archa, 1 ♀, vii. 2000, 1 ♀, vii. 2002, V. Gurko lgt., OLML; **Poland**: Toruń-Glinki, 1 ♀, 5. vii. 2011, P. Olszewski lgt., JSPC; **Slovakia**: Chotín, 1 ♂, 15. vii. 1967, A. Strejčková lgt., OLML; **Turkey**: Erzurum E, Erzurum prov., 1 ♂, 6. vii. 2000, M. Halada lgt., OLML; Hakkari env., 1 ♀, 17. ix. 1983, Ch. Schmid-Egger lgt., CSE; Mt. Gilo, Hakkari prov., 1 ♂, 15. viii. 1972, Kaniss lgt., ZSM.

##### Diagnosis.

*Tachysphex nigripennis* (Spinola) is easily recognizable from the other species of the *Tachysphex pompiliformis* and *Tachysphex austriacus* species subgroups. It is generally dark and coarsely punctate, and thus resembles *Tachysphex opacus* Morawitz and *Tachysphex pompiliformis* (Panzer). *Tachysphex nigripennis* (Spinola) is relatively well characterized species. It possesses an inconspicuous gena. The vertex is distinctly wider than long. The metasoma is coarsely punctate with densely micropunctate apical depressions. The mesopleuron is coarsely sculptured, rugose, or coarsely punctate, and the hypoepimeral area has more or less distinct longitudinal ridges. The metasoma has terga I-II dark red and a black sternum II. In females, the basomedian area of the clypeus is sparsely punctate; the pygidium is exceptionaly narrow, bright shiny and with inconspicuous lateral marginal carina. In males, the mandibular inner margin possesses one distinct rectangular tooth and a distinct furrow next to the tooth distally; the clypeal lip is distinctly arcuate, longest medially; the forefemoral notch is dark red but black in some specimens, with a shiny, nearly unsculptured surface; the dorsal process of volsella is larger than in species from the *Tachysphex pompiliformis* species subgroup.

##### Description of female

(neotype). Body length: 8.9 mm.

Head. Clypeus distinctly convex, top at clypeal midlength; basomedian area relatively large, as sparsely punctate as lateral section, punctures small, ill-defined, interspaces variable, one to less than half diameter apart, shiny; bevel large, distinctly convex, triangular, with sparse large punctures, not reaching base of clypeus, shiny; lip distinctly sinuate, long medially, with poorly developed lateral incision, lip separate from bevel by punctate groove, WML:LCL = 1.7, WCL:WML = 1.8. Supraclypeal area flat, finely, sparsely punctate, punctures ill-defined, interspaces microsculptured, slightly shiny. Supraantennal tubercle small, slightly elevated on inner side. Antenna relatively short, LA3:WA3 = 2.5, LA5:WA5 = 3.1. Frons uniformly punctate, punctures well defined, less than one diameter apart, interspaces variable in size, microsculptured, slightly shiny; frontal median line distinct, narrow, finely impressed. Vertex finely, densely punctate, punctures well defined, one to less than half diameter apart, interspaces variable in size, coarsely microsculptured, slightly shiny. Vertex setae short, semierect medially, but nearly erect close to inner eye margin, less than 1 × MOD, postocellar impression distinct, shallow, open widely Y-shaped; vertex distinctly wider than long; WV:LV = 1.6. Gena dorsally short, converging directly behind eyes.

Mesosoma. Scutum without distinct anterior impression; scutum and scutellum densely punctate, punctures well defined, less than one diameter apart, interspaces variable in size, microsculptured, slightly shiny, setae about 1.0 × MOD or less. Mesopleuron rugose, posteriorly coarsely microsculptured, without distinct punctures; hypoepimeral area iregularly rugose with indistinct irregular longitudinal ridges, impunctate; ventral part of mesopleuron sparsely punctate, punctures ill defined, one diameter apart, slightly shiny. Propodeal dorsum relatively regularly rugose, without longitudinal ridges, dull; propodeal side irregularly longitudinally ridged, microsculptured, dull; posteromedial margin of propodeal dorsum elevated, slightly produced between marginal ridges, marginal ridges positioned nearly horizontally above groove on posterior side. Legs densely punctate, punctures small; forebasitarsal rake dark reddish, with two apical spines, one preapical spin and one or two additional spines. Wings distinctly infumate, light brown, with brown veins.

Metasoma. Terga I-III with distinct but sparse silvery, apical faciae. Terga I-III densely micropunctate, with very large, but superficial, sparse punctures, micropunctures ill-defined, evanescent in micosculpture, faint, interspaces microsculptured, slightly shiny; apical depressions well developed, with distinct micropunctures; sculpture of terga III-V distinctly coarser than other terga, also more densely punctate; tergum V coarsely punctate, punctures ill defined, but large, half to one diameter apart, interspaces microsculptured, apical depression of tergum V distinctly, densely finely punctate. Pygidium distinctly narrower than in other related species, integument sparsely punctate, large and small punctures intermixed, punctures ill defined, interspaces nearly unsculptured, bright shiny. Central part of sternum II with several large punctures, interspaces microsculptured, shiny; lateral part slightly shiny, densely micropunctate; remaining sterna with uniform sculpture similar to that on sternum II, but more or less reduced laterally.

Coloration. Central part of mandibles, terga I-II and distal tarsomeres dark red. Tegulae brown translucent. Apical parts of terga I-III slightly translucent. Remaining body parts all black.

Variation of females: Body length: 7.0–10.1 mm. WML:LCL = 1.5–1.7, WCL:WML = 1.8–1.9. WV:LV = 1.5–1.6.

##### Description of male.

Body length: 6.4–7.9 mm.

Head. Mandibular inner margin with one distinct rectangular tooth and distinct furrow next to tooth distally. Clypeus convex, slightly elevated, distinctly declining apically, top variably located between apical third and midlength; basomedian area large, sparsely punctate, more sparsely punctate medially in some specimens; bevel convex, decreasing toward clypeal lip, variable in size, reaching base of clypeus in most specimens, shiny, with several large punctures; lip distinctly sinuate, conspicuously produced medially, with more or less distinct lateral corner, lip separated from bevel by groove; WML:LCL = 1.2–1.3, WCL:WML = 2.3–2.4. Supraclypeal area slightly concave, distinctly punctate, punctures ill-defined, interspaces between punctures coarsely microsculptured, slightly shiny to dull. Supraantennal tubercle small, slightly elevated on inner side, shiny. Antenna relatively short, LA3:WA3 = 1.5–1.6, LA5:WA5 = 1.9–2.1. Frons uniformly punctate, punctures well defined, less than one diameter apart, interspaces variable in size; frontal median line distinct, narrow, finely impressed, or indistinct. Vertex uniformly, finely and densely punctate, punctures well defined, one to less than half diameter apart, interspaces variable in size, coarsely microsculptured, slightly shiny to dull. Vertex setae short, erect medially, but semierect close to inner eye margin, less than 1 × MOD, postocellar impression large, but shallow, open widely V to Y-shaped, densely punctate, punctures relatively small; vertex distinctly wider than long; WV:LV = 1.6. Gena dorsally inconspicuous.

Mesosoma. Scutum with shallow anterior impression, densely punctate, punctures well defined, most punctures less than half diameter apart, interspaces distinct, microsculptured, slightly shiny to dull, setae about 1.0 × MOD or less; scutellum slightly more sparsely punctate than scutum, punctures well defined, most punctures half to less than one diameter apart, interspaces shiny to slightly shiny. Mesopleuron rugose to densely punctate, most punctures in ventral half well defined, interspaces coarsely microsculptured to rugose, dull; hypoepimeral area coarsely rugose, without distinct punctures, with distinct longitudinal ridges in most specimens, dull; ventral part of mesopleuron with small ill-defined punctures, interspaces finely microsculptured, slightly shiny. Propodeal dorsum coarsely sculptured, irregularly areolate to irregularly coarsely ridged, without longitudinal ridges except at very base; propodeal side longitudinally ridged, ridges well developed, ill defined in small specimens, interspaces microsculptured, dull; posteromedial margin of propodeal dorsum slightly elevated or not elevated, marginal ridges on posterior surface well developed, horizontal, or slightly directed toward propodeal groove medially. Legs micropunctate, punctures small; forefemoral notch large, deep, semicircular, about as long as distance that separates it from forefemoral base or slightly longer, proximal margin obtuse, not elevated over distal margin, central part of notch inconspicuously elevated, anterior and posterior margin lined by nearly indistinct ledge, notch surface without distinct setae, unsculptured, or very finely microsculptured, shiny. Wings infumate, with brown veins.

Metasoma. Silvery apical fasciae of terga I-III distinctly developed, but fasciae brown in specimens from eastern Turkey. Apical depressions of all terga shallow, densely micropunctate, nearly as densely as on tergal disk. Terga I-III densely and distinctly micropunctate, punctures ill defined, interspaces microsculptured, slightly shiny; terga IV-VII more coarsely and more densely punctate than previous terga. Sterna uniformly punctate nearly like terga.

Coloration. Apical part of mandibles red; terga I and II dark red; apex of sternum I and base of sternum II dark red in some specimens; distal tarsomeres dark red on ventral side; forefemoral notch dark red in most specimens, but black in some. Tegulae brown translucent. Apical parts of terga slightly translucent. Remaining body parts all black.

##### Geographic Distribution.

Czech Republic, France, Germany, Hungary, Italy, Kyrgyzstan, Poland, Slovakie and Turkey.

#### Tachysphex nobilis sp. n.

Taxon classificationAnimaliaHymenopteraCrabronidae

http://zoobank.org/B19428FC-A83E-4E18-86BF-494F6EA075AC

Figs 7 , 19 , 33 , 40 , 47 , 73 , 99 , 108

##### Type material.

Holotype: ♀, Hungary: “HUNGARY centr. / ÖRKENY env. / 15.VI.2007 / P. Bogusch lgt. // ♀ Tachysphex / nobilis / sp.n. / Jakub Straka det.2009 // ZSM / HYM 23689”. Holotype in ZSM. Paratypes: **Bulgaria**: Albena-Kraněvo, 1 ♀, 4. vii. 1978, Z. Pádr lgt., OLML; Asenovgrad env., 1 ♂, 15. vii. 1969, K. Deneš lgt., JSPC; Borika SW, Sredna Gora Mts., 1 ♂, 6. viii. 2010, T. Ljubomirov lgt., IBER; Konstantinovo N, 1 ♂, 26. vi. 2012, T. Ljubomirov lgt., IBER; Kurtovo Konare S, 1♂, 3. viii. 2012, T. Ljubomirov lgt., IBER; Pasarel NW, Isskar valey, 1 ♂, 2 ♀♀, 6. vi. 2009, T. Ljubomirov lgt., IBER; Slančev Brjag env., 3 ♀♀, 14. vii. 1971, 1 ♂, 2. vii. 1978, Z. Pádr lgt., OLML Z. Pádr lgt., OLML, 2 ♀♀, 10. vii. 1983, P. Tyrner lgt., PTLC, 1 ♀, 30. v. 1989, J. Halada lgt., OLML; Vlahi, 1 ♂, 3 ♀♀, 14. viii. 1993, M. Halada lgt., JSPC; Vlahinska Reka, Strouma valey, 1 ♂, 29. vi. 2008, T. Ljubomirov lgt., IBER; Sandanski env., 1 ♂, 13. vii. 1966, M. Kocourek lgt., OLML; **Czech Republic**: Mor.mer., Bzenec env., 1 ♂, 14. vi. 1942, 1 ♀, 21. vi. 1942, A. Hoffer lgt., OLML, JSPC; Vracov env., 1 ♀, 21. viii. 1942, A. Hoffer lgt., OLML; **Greece**: Petritsio SE, Strymon valley, Hellas prov., 1 ♀, 21. v. 2009, G. Ramel lgt., IBER; **Hungary**: Örkeny, puszta, 1 ♂, 18. v. 1985, J. Halada lgt., JSPC; Iszák, 1 ♀, 18. v. 1989, Z. Karas lgt., ZKZC; Sándorfalva env., 1 ♀, 19. v. 1988, Z. Karas lgt., ZKZC; **Poland**: Karwia, Władysławowo, Baltic see coast, 1 ♀, 24. vi. 1996, R. Dobosz lgt., USMB; Pasturka, Pińczów env., Malopolska Upland, 1 ♀, 10. vi. 1998, R. Dobosz lgt., USMB; Pustynia Błędowska, Gmina Klucze, 1 ♀, 30. vi. 1992, W. Żyła lgt., USMB; Sarbinowo, Baltic see coast, 1 ♀, 18. viii. 1995, J. Batleja lgt., USMB; Toruń-Glinki, 1 ♀, 14. vi. 2011, P. Olszewski lgt., JSPC; Wicie, Baltic see coast, 1 ♀, 18. vii. 2004, W. Żyła lgt., USMB; Wicie, Baltic see coast, 1 ♀, 23. vii. 2004, W. Żyła lgt., USMB; Wicie, Baltic see coast, 1 ♀, 28. vii. 2004, W. Żyła lgt., USMB; Wicie, Baltic see coast, 1 ♂, 1 ♀, 25. vii. 2004, W. Żyła lgt., USMB, JSPC; Wisełka env., Wolin, Baltic see coast, 1 ♂, 1 ♀, 25. vii. 1997, R. Dobosz lgt., USMB; Władysławowo, 1 ♂, 30. vi. 1993, R. Dobosz lgt., USMB; **Turkey**: Ahlat, near Van lake, 1 ♂, 14. vii. 1996, P. Tyrner, Voříšek lgt., PTLC; Çamardı, Niğde prov., 1 ♂, 13. vii. 1997, M. Halada lgt., OLML; Ilhara env., valley, Aksaray prov., 2 ♂♂, 13. vi. 2008, M. Obořil lgt., JSPC.

##### Additional material examined.

**Poland**: Wisełka env., Wolin, Baltic see coast, 1 ♂, 25. vii. 1997, R. Dobosz lgt., USMB; **Turkey**: Ilhara env., valley, Aksaray prov., 1 ♂, 13. vi. 2008, M. Obořil lgt., JSPC.

##### Diagnosis.

*Tachysphex nobilis* sp. n. is most similar to *Tachysphex bohemicus* sp. n. and *Tachysphex jokischianus* (Panzer) in having fine and dense body sculptures and sparsely punctate apical margin of tergum V in female. *Tachysphex nobilis* sp. n. is a characteristic and relatively easily recognizable species. It is generally slender in body, finely, densely punctate throughout, and possess an insignificantly convex clypeus, with the bevel small in the female and nearly absent in the male. Both sexes also have the gena dorsally inconspicuous, converging behind the compound eyes. The lateral emargination of the clypeal lip in the female is round, not angulated. The propodeal dorsum in the male has a finely sculptured longitudinal area posteromedially, which appears slightly shinier than the adjacent parts of the propodeum.

##### Description of female (holotype).

Body length: 8.9 mm.

Head. Clypeus slightly convex, top at clypeal midlength; basomedian area cover majority of space of clypeus, as densely punctate as lateral section; bevel very small, slightly convex to flat, only about 2–3 × as long as clypeal lip, slightly longer medially than laterally, with few large punctures, ending far from base of clypeus, shiny; lip insignificanly arcuate to nearly straight, with faint lateral incision, separate from bevel by punctate groove, WML:LCL = 1.6, WCL:WML = 1.8. Supraclypeal area flat, finely and densely punctate, punctures ill defined, interspaces microsculptured, dull. Supraantennal tubercle small, slightly elevated on inner side. Frons uniformly, very densely punctate, punctures well defined, less than half diameter apart, interspaces uniform in size, dull; frontal median line distinct, shiny, not impressed. Vertex finely, densely punctate, punctures well defined, half to less than half diameter apart, interspaces finely microsculptured, slightly shiny. Vertex setae short, semierect, about 0.5 × MOD; postocellar impression well developed, widely Y-shaped; vertex slightly wider than long; WV:LV = 1.2. Gena dorsally well developed.

Mesosoma. Scutum without distinct anterior impression; scutum and scutellum densely punctate, punctures well defined, less than half diameter apart, interspaces well developed and relatively uniform in size, microsculptured, slightly shiny, setae about 1.0 × MOD or less. Mesopleuron coarsely microsculptured, without distinct punctures; hypoepimeral area finely rugose with indistinct, longitudinal, irregular ridges, without punctures; ventral part of mesopleuron finely, densely punctate, with punctures ill defined, less than one diameter apart, interspaces small, slightly shiny. Propodeal dorsum relatively finely, regularly rugose, with short irregular longitudinal ridges basally, dull, posteromedially with transverse ridges on small longitudinal slightly shiny area; propodeal side regularly longitudinally ridged, ridges inconspicuous basally, microsculptured, dull; posteromedial margin of propodeal dorsum elevated, slightly produced between marginal ridges, marginal ridges positioned nearly horizontally above groove on posterior surface. Legs densely punctate, punctures small; forebasitarsal rake light reddish, with three apical spines, one preapical and one or two additional ones. Wings slightly infumate, yellowish, with brown veins.

Metasoma. Terga I-III with distinct silvery, apical faciae; terga I-II very densely and finely micropunctate, punctures ill-defined but distinct, interspaces microsculptured, slightly shiny; apical depressions well developed, with distinct micropunctures; sculpture of terga III-V distinctly coarser than on previous terga, also more densely punctate; tergum V coarsely punctate, punctures ill-defined, but large, half to one diameter apart, interspaces microsculptured, apical depression of tergum V very sparsely, finely punctate. Pygidium of usual size, sparsely punctate, punctures large, ill defined, interspaces microsculptured basally, but unsculptured in apical half, slightly shiny to shiny. Central part of sternum II with several large punctures, interspaces microsculptured, shiny; lateral part slightly shiny, densely micropunctate; remaining sterna with uniform sculpture similar to that on sternum II, but more or less reduced laterally.

Coloration. Central part of mandibles, terga and sterna I-III red. Distal tarsomeres are dark red. Tegulae brown translucent. Apical parts of terga I-III slightly translucent. Remaining body parts all black.

Variation of females: Body length: 7.2–9.0 mm. Antenna relatively short, LA3:WA3 = 1.9–2.1, LA5:WA5 = 2.9–3.0. In general, the species is not significantly variable in key characters. There is the usual variation in size dependent characters like the number of spins on foretarsal rake. The shape of the lateral emargination of the clypeal lip is one of a few variable characters; it can be well developed to nearly absent, but never angulated. The pygidium is slightly variable in width and sculpture; it is of usual shape in most specimens with distinct microsculpture, slightly shiny in basal half, but also wider and bright shiny in some specimens.

##### Description of male.

Body length: 5.4–6.8 mm.

Head. Mandibular inner margin with one distinct rectangular tooth and distinct furrow next to tooth distally. Clypeus very slightly convex (appearing nearly flat), uniformly curved, top at about clypeal midlength; basomedian area dominant on clypeus, densely, uniformly punctate; bevel nearly or completely absent, reduced to shiny area ventromedially; lip arcuate or slightly sinuate, with small rectangular lateral corner, lip separated from bevel by distinct, punctate groove; WML:LCL = 0.9–1.2, WCL:WML = 2.2–2.4. Supraclypeal area groove-like, slightly concave, supraantennal tubercles slightly elevated ventromedially, circular; surface coarsely microsculptured with indistinct punctures, dull. Antenna relatively short, LA3:WA3 = 1.5–1.6, LA5:WA5 = 1.9–2.1. Frons finely punctate, punctures well defined, half to less than half diameter apart, interspaces relatively uniform in size; frontal median line distinct, narrow, finely or not impressed, shiny. Vertex finely punctate, punctures well defined, one to less than one diameter apart, interspaces microsculptured, slightly shiny to dull. Vertex setae short, semierect, less than 1 × MOD; postocellar impression distinctly impressed, but shallow, open widely V to Y-shaped; vertex moderately wider than long, WV:LV = 1.5. Gena dorsally inconspicuous.

Mesosoma. Scutum without distinct anterior impression; scutum and scutellum densely punctate, punctures well defined, most punctures less than half diameter apart, interspaces distinct, microsculptured, slightly shiny to dull, setae about 1.0 × MOD or less. Mesopleuron coarsely microsculptured, with distinct or indistinct punctures in lower areas, most punctures ill defined, interspaces dull; hypoepimeral area coarsely microsculptured to finely rugose, dull; ventral part of mesopleuron densely punctate, punctures small ill defined, interspaces slightly shiny. Propodeal dorsum finely irregularly areolate to rugose, without longitudinal ridges, posteromedially with finely sculptured longitudinal area, which appears slightly shinier than adjacent areas; lateral surface of propodeum coarsely microsculptured, with or without ill defined longitudinal ridges, dull; posteromedial margin of propodeal dorsum elevated, but not produced between marginal ridges, marginal ridges positioned nearly horizontally above groove on posterior side. Legs densely punctate, punctures small; forefemoral notch light red and large, semicircular, its diameter is usually longer than distance that separates it from forefemoral base, central part of notch slightly elevated, anterior and posterior margins lined by small ledge, nearly indistinct in some specimens, notch surface without distinct setae, microsculptured, dull. Wings slightly infumate, veins brown.

Metasoma. Terga I-III with silvery apical faciae. Apical depressions of terga distinct, micropunctate similarly to that on tergal disk. Terga I-III densely, distinctly micropunctate, punctures ill defined, interspaces microsculptured, slightly shiny; sculpture of terga IV-VII slightly coarser than on previous terga, also punctures slightly denser. Sterna uniformly punctate, nearly like terga.

Coloration. Apical part of mandible, terga and sterna I-III and forefemoral notch red. Distal tarsomeres reddish. Tegulae brown to reddish. Apical parts of all terga and tegulae translucent. Remaining body parts all black.

##### Geographic distribution.

Bulgaria, Czech Republic, Greece, Hungary, Poland and Turkey.

##### Name derivation.

The species is named after its uniform sculptures. The species appears as a “noble” among other species of the *Tachysphex pompiliformis* species subgroup.

#### Tachysphex opacus

Taxon classificationAnimaliaHymenopteraCrabronidae

F. Morawitz, 1893

Figs 10 , 11 , 25 , 34 , 67 , 74 , 100

##### Material examined.

**Iran**: Kherameh, Fars prov., 1 ♀, 24. i. 2013, E. Izadi lgt., IBER; Neyriz-Jaafarabad, Fars prov., ira-fa01, 1 ♂, 2 ♀♀, 7. ix. 2012, M. Khosroabadi lgt., CSE; **Kazakhstan**: Mangismlak, Kamysta (90 km Etaoutchik), 1 ♂, 8. v. 1962, Malkovskii, ZIN; **Kyrgyzstan**: Dzahal-Abad area, 2 ♂♂, 1 ♀, vi. 2000, V. Gurko lgt., JSPC, OLML; Kaltabulak road gorge, Transalai Mt. r., Alay prov., 1 ♀, 15. vii. 1998, I. Makogonova lgt., OLML; Kirghiz-Ata grg., Kitchik-Alai Mt., Nookat distr., 1 ♂, 25. vi. 1996, S. L. Zonstein lgt., JSPC; **Tajikistan**: Dushanbe env., 1 ♂, 15. vi. 1966, K. Deneš lgt., OLML; Gušari (Chuš-er), Gissar., 1 ♂, 26. vii. 1937, Gussakovskii, ZIN; Rushan 30 km N, Pamir Mts., 1 ♀, vii. 2000, V. Gurko lgt., JSPC; **Turkey**: Ahlat, near Van lake, Van prov., 1 ♀, 14. vii. 1996, P. Tyrner, Voříšek lgt., PTLC; Muradye 40km N, Van prov., 1 ♂, 5. vii. 2000, M. Halada lgt., OLML; **Uzbekistan**: Aman-Kutan pass., Zerevshan Mt., w. part, Samarkand prov., 1 ♀, 8. vi. 1995, S. L. Zonstein lgt., OLML; Bashkyzylsai river canyon, Tchatkal Mts., Parkent distr., 1 ♀, 5. viii. 1999, S. L. Zonstein lgt., OLML; Czirczik, Yangiyul District, 1 ♀, 28. v. 1994, J. Halada lgt., JSPC; Dzhizak vil., Douba 5km S, Turkest. Khrebet, Zaamin distr., 1 ♂, 13. vi. 1997, H. & R. Rausch lgt., OLML; Kainarsai, Ugam Mt. r., Bostanlik distr., 1 ♀, 24. vii. 1996, S. L. Zonstein lgt., OLML. Specimens determined as *Tachysphex opacus* aff.: **Jordan**: Petra 10km N, 1 ♂, 3. v. 1996, M. Halada lgt., OLML; **Kazakhstan**: Fabritchny, 40 km E Almaty, Zhambyl distr., 1 ♂, 9. vii. 1992, Jirousek lgt., JSPC; Ushtagan, Sauskan sands, Aktau 120km E, 1 ♀, 15. v. 2000, J. Matleuski lgt., JSPC; **Syria**: Damascus 40km NE, 2 ♂♂, 13. v. 1996, Mi. Halada lgt., OLML; Dibbin, Suwayda 30km S, 2 ♂♂, 17. v. 1996, Mi. Halada lgt., OLML; Homs 50km S, 1 ♀, 24. v. 1996, Mi. Halada lgt., OLML; Khabab, Damascus 60km S, 1 ♂, 14. v. 1996, Mi. Halada lgt., OLML; Marbij env., 1 ♂, 9. v. 1996, Mi. Halada lgt., OLML; Turkey: Çaykavak, Ulukışla, Niğde prov., 1 ♂, 30. v. 2001, M. Snížek lgt., OLML; Gürün 20km E, Malatya prov., 1 ♂, 10. vii. 1997, M. Halada lgt., OLML; Kengerlidüz, Hatay, 1 ♂, 17. v. 2008, M. F. Gürbüz lgt., IBER; Ürgüp 10km W, Nevşehir prov., 3 ♂♂, 15. vi. 1998, M. Halada lgt., OLML.

##### Diagnosis.

*Tachysphex opacus* Morawitz is easily recognizable from other species of the *Tachysphex pompiliformis* and *Tachysphex austriacus* species subgroups. This species is generally dark and has coarsely sculptured mesopleuron and terga. Based on this characteristic, *Tachysphex opacus* resembles *Tachysphex nigripennis* (Spinola) and *Tachysphex pompiliformis* (Panzer). It differs from *Tachysphex pompiliformis* (Panzer) in having the gena inconspicuous and a sparsely punctate clypeal basomedian area. In the females, the metasoma has dark red terga I-II and a black sternum II, and the vertex is nearly as wide as long. In the males, the body is black, the mandibular inner margin is shallowly emarginated distally from inner tooth, with no furrow, the clypeal lip is nearly straight, the vertex is distinctly wider than long. In addition to the mentioned characters, the females of *Tachysphex opacus* Morawitz differ from *Tachysphex nigripennis* (Spinola) in having normal sized pygidium with microsculpture and lacking a bright shine. The males differ in having a forefemoral notch microsculptured, dull, and the dorsal process of the volsella narrow.

##### Geographic distribution.

China, Iran, Kazakhstan, Kyrgyzstan, Tajikistan, Turkey, and Uzbekistan. It is probably also distributed in Jordan, Lebanon and Syria.

##### Note.

Holotype came from China (Uighur region). Type material not examined. The species was redescribed by Pulawski (1971).

#### Tachysphex pompiliformis

Taxon classificationAnimaliaHymenopteraCrabronidae

(Panzer, 1804)

Figs 9 , 12 , 20 , 26 , 35 , 48 , 52 , 58 , 68 , 75 , 85 , 90 , 101

Larra pompiliformis Panzer, 1804 (Heft 89: pl. 13), ♀.

##### Type material.

Holotype: ♀, no specific locality at the label, but probably from Germany. Holotype by monotypy. “Coll. Sturm [printed] // Stimmt / Larra / pompili - / formis / Panz. [handwriting, card with black frame] // 56 [printed, red card] // Larra / pompiliformis Panz. / Typus sec. Richards 1935 [handwriting] // Tachysphex ♀ / pompiliformis Pz. / J. de Beaumont / det. 1955 [handwriting on pre-printed card with name of determinator and year] // Typus Nr. / Zoologische / Staatsamlung / München. [printed, red card]”. Holotype in ZSM, examined.

##### Additional material examined.

**Austria**: Seefeld NW, Niederösterreich, 1 ♂, 19. v. 2015, M. Halada lgt., OLML; **Czech Republic**: Boh. occ., Tušimice, ash coal deposit from power station, 3 ♂♂, 26. vi. 2010, 1 ♂, 31. vii. 2010, ZSM HYM24250, 3 ♀♀, 22. viii. 2010, ZSM HYM24580, R. Tropek lgt., JSPC,; Mor. mer., Boleradice 1 km NE, 1 ♂, 10. vii. 2014, M. Říha lgt., JSPC; Bratčice env., 1 ♂, 21. vi. 2012, M. Halada lgt., JSPC; Čejč, 1 ♂, viii. 1963, Z. Pádr lgt., OLML; Gotberg, Popice 1.5km NE, 1 ♂, 25. v. 2014, ZSM HYM24595, M. Říha lgt., JSPC; Havraníky env., NP Podyjí, 1 ♂, 18. v. 2015, M. Halada lgt., JSPC; Morkůvky 1km NE, 1 ♂, 8. vii. 2014, M. Říha lgt., JSPC; **Bulgaria**: Martisganitsa cotage W, 1 ♂, 24. viii. 2013, T. Ljubomirov lgt., IBER; Milanovo W, 1 ♂, 7. vii. 2015, T. Ljubomirov lgt., IBER; Razdol, Maleshevska Planina, 2 ♂♂, 27. vi. 2008, T. Ljubomirov lgt., IBER; Rezhantsi env., 1 ♀, 9. viii. 2011, N. Simov lgt., IBER; Slančev Brjag env., 1 ♀, 18. vi. 1977, Z. Pádr lgt., 1 ♂, 29. vii. 1968, M. Kocourek lgt., OLML; Staroplaninets cotage SW, Etropolska Planina, 1 ♀, 24. viii. 2006, 1 ♂, 1 ♀, 1. vii. 2010, T. Ljubomirov lgt., IBER; Staroseltsi env., Isskar valey, 2 ♂♂, 28. v. 2011, T. Ljubomirov lgt., IBER; Trubatch, Razgradski heights, 1 ♀, 22. viii. 1999, K. Ivanov lgt., JSPC; **France**: St. Thomé, Font Just, 12 km SW Montélimar, 1 ♀, 14. vi. 2001, J. v. d. Smissen lgt., USMB; **Greece**: Ag. Petros, Meg. Tourla, Oros Parnon, Hellas, 1 ♀, 14. vii. 2006, 2 ♂♂, 24. vi. 2013, W. Arens lgt., WAPC; Ano Trikala, Mt. Killini, Hellas, 8 ♂♂, 24. vi. 2008, W. Arens lgt., WAPC; Meg. Tourla, Oros Parnon, Hellas, 1 ♀, 13. vii. 2006, 5 ♂♂, 23. vi. 2013, W. Arens lgt., WAPC; Michas, Erymanthos, Achaea, 1 ♀, 23. vi. 1995, 1 ♀, 10. vii. 1996, W. Arens lgt., WAPC; Prof. Ilias, Oros Taygetos, Hellas, 2 ♂♂, 28. vi. 2013, ZSM HYM24579, W. Arens lgt., WAPC, JSPC; Stymphalia, Hellas, 1 ♀, 15. vi. 1995, W. Arens lgt., WAPC; Toriza-Prof. Ilias, Oros Taygetos, Hellas, 1 ♀, 11. vii. 2007, W. Arens lgt., WAPC; **Hungary**: Hegyshalom N, 2 ♂♂, 6. vi. 2015, M. Halada lgt., OLML; Kesthely env., lime stone, 1 ♂, 1 ♀, 23. v. 2014, A. Astapenková, J. Habermannová, J. Straka lgt., JSPC; Örkeny, puszta, 2 ♂♂, 27. vi. 2015, M. Halada lgt., JSC; Pákozd env., pasture stepe, 4 ♂♂, 10. vi. 2015, 2 ♂♂, 1 ♀, 25. vi. 2015, M. Halada lgt., JSPC; **Italy**: Coldrano, Vinschgau, Trentino-Alto Adige prov., 1 ♂, 8. vi. 2007, Ch. Schmid-Egger lgt., CSE; Lanzada, Sondrio 11 km N, Lombardia prov., 2 ♀♀, 9. vii. 2006, Ch. Schmid-Egger lgt., CSE; Lasa, Vinschgau 2 km N, Trentino-Alto Adige prov., 6 ♂♂, 3 ♀♀, 8. vi. 2007; Ch. Schmid-Egger lgt., CSE; Osis/Eyrs, Vinschgau, Trentino-Alto Adige prov., 4 ♂♂, 1 ♀, 8. vi. 2007, Ch. Schmid-Egger lgt., CSE; Pondel, Aosta 8 km SW, Valle d’Aosta prov., 3 ♂♂, 4. viii. 1996, P. Rosa lgt., CSE; Ponte in Valtellina, Sondrio 10 km E, Lombardia prov., 3 ♂♂, 9. vii. 2006, Ch. Schmid-Egger lgt., CSE; St. Pierre, Valle d’Aosta prov., 2 ♂♂, 1. viii. 1997, Ch. Schmid-Egger lgt., CSE; Tubre/Taufers, Vinschgau 1 km NE, Trentino-Alto Adige prov., 8 ♂♂, 2 ♀♀, 27. vii. 2007, Ch. Schmid-Egger lgt., CSE; Casali Santa Maria Maddalena N, 1 ♂, 22. vi. 2011, T. Ljubomirov lgt., IBER; Casali Santa Maria Maddalena NE, 6 ♂♂ 22. vi. 2011, T. Ljubomirov lgt., IBER, CCDB-12231-G09, CCDB-12230-A04, CCDB-12230-C01, CCDB-12232-B08, CCDB-12233-D06, CCDB-12233-F01; Ozein, 10km SW Aosta, I-aooz, 1 ♂, 19. viii. 1996, P. Rosa lgt., ZSM; Saint-Pierre, 16km W Aosta, Valle d’Aosta, I-aoB, 1 ♂, 8. vii. 1995, 1 ♀, 27. vi. 1996, Ch. Schmid-Egger lgt., CSE; Saint-Pierre, 6 km W Aosta, Valle d’Aosta, 2 ♂♂, 28. vi. 1999, J. v. d. Smissen lgt., USMB; Sarre, Aosta valey, 1 ♀, 1. viii. 2007, W. Schlaefle lgt., WSKC; Tartsch, Bolzano, 1 ♀, 28. vii. 1989, J. Tiefenthaler lgt., OLML; **Kyrgyzstan**: Ak-Buura ravine, Osh 45km S, Alai Mts., Osh prov., 1 ♂, 14. vii. 2014, Oistein Berg lgt., JSPC, ZSM HYM23639; Issik-Kul lake ost., Teplokljitchenka rg., 3 ♂♂, 3 ♀♀, viii. 2001, V. Gurko lgt., JSPC; Kashkasu env., Ala-Archa, 1 ♂, 9. vii. 2002, V. Gurko lgt., JSPC; Tchon-Aryk, Kirghizskyi Mts., 1 ♂, 11. vii. 2001, V. Gurko lgt., JSPC; **Portugal**: Armamar, Viseu distr., P-arn, 1 ♂, 1. viii. 2001, Ch. Schmid-Egger lgt., CSE; Gerês, Braga distr., P-ger, 2 ♂, 31. vii. 2001, Ch. Schmid-Egger lgt., CSE; **Slovakia**: Štúrovo, 1♂, 28. vii. 1959, 1 ♂, 18. vi. 1960, 1 ♀, 2. viii. 1960, 1 ♂, 15. viii. 1961, 1 ♀, 16. vii. 1962, 2 ♂♂, 1 ♀, 27. vii. 1962, Z. Pádr lgt., OLML, 1 ♀, 29. vii. 2002, J. Habarta lgt., JSPC; Somotor, 1 ♀, vii. 1958, M. Kocourek lgt., OLML; **Switzerland**: Leuk, Wallis prov., 1 ♀, 12. viii. 2011, W. Schlaefle lgt., WSKC, Mergoscia, Tessin prov., 1 ♂, 15. vii. 2011, W. Schlaefle lgt., WSKC; Monte Comino, Tessin prov., 1 ♀, 15. vii. 2007, 1♂, 11. vii. 2012, W. Schlaefle lgt., WSKC; Zeneggen, Wallis prov., 1 ♂, 15. vii. 2010, W. Schlaefle lgt., WSKC; **Turkey**: Dortiol, Amánus Mts., 4 ♂♂, 10. vi. 2008, M. Škorpík lgt., JSPC; Erzurum E, Erzurum prov., 2 ♂, 6. vii. 2000, M. Halada lgt., OLML; Esendere, Hakkari prov., 1 ♀, 21. vii. 1988, Ch. Schmid-Egger lgt., CSE; Ihara valey, Adana prov., 1 ♂, 13. vi. 2008, M. Kafka lgt., JSPC; Kasnak Mesesi, Isparta, 1 ♀, 28. vi. 2007, 1 ♀, 30. vi. 2007, 1 ♀, 8. ix. 2007, A. Bayindir lgt., IBER, 1 ♂, 6. viii. 2008, T. Ljubomirov, A. Bayindir, IBER; Kengerlidüz, Hatay, 1 ♀, 21. vii. 2007, M. F. Gürbüz lgt., IBER; Mitisin, Osmaniye prov., 1 ♂, 6. viii. 2008, T. Ljubomirov lgt., IBER; Muradiye, Van prov., 1 ♀, 3. vii. 2000, M. Halada lgt., OLML; Pazaryolu, Kumaşkaya, Erzurum prov., 1 ♂, 25. vi. 2014, T. Ljubomirov lgt., IBER; Pozantı, Adana prov., 1 ♂, 6. vii. 1984, Hladil lgt., OLML; Refahye 15km W, Erzincan prov., 1 ♀, 7. vii. 2000, M. Halada lgt., OLML; Salihli 35 km SEE, Manisa prov., 1 ♂, 30. vi. 2006, M. Halada lgt., JSPC; Samson University campus, Samson prov., 1 ♂, 4. vii. 2014, J. Barták, Kubík lgt., JSPC; Tekederesi env., Erzurum vil., 15 km SW, 2 ♂♂, 2. vii. 2001, M. Fikáček, J. Hájek, J. Straka lgt., JSPC; Yüksekova, Hakkari prov., 1 ♀, 22. vii. 1988, Ch. Schmid-Egger lgt., CSE.

##### Diagnosis.

*Tachysphex pompiliformis* (Panzer) is difficult to distinguish from *Tachysphex dimidiatus* (Panzer), *Tachysphex jokischianus* (Panzer) and *Tachysphex punctipleuris* sp. n. It is exceptionally variable in most characters and no single character alone can differentiate it from morphologically similar species. This species is generally darker and more coarsely punctate than similar species. The metasoma is densely micropunctate, including apical depressions. The mesopleuron is coarsely sculptured, rugose, or coarsely punctate, and the hypoepimeral area has more or less distinct longitudinal ridges. In males, sternum II is often dark red to partly black, but tergum II is red; however, specimens with red metasomal segments I-III also occur. The terga are darker than in other species in some females, but this occurs less often. The forefemoral notch of the male is dark in most specimens. The gena is robust. The following combination of additional characters is also characteristic of this species: ♀: The clypeus is slightly convex, and the most elevated point is not well defined but located at about the clypeal midlength. The vertex setae are short and uniformly semierect both medially and laterally. The dorsolateral cuticular projection of the propodeal spiracle is arcuate to semicircular and usually dark. The propodeal dorsum and side are longitudinally ridged; the dorsum is coarsely sculptured, but the ridges are indistinct in some specimens. The forebasitarsal rake is reddish in fresh individuals, with two, well separated apical spines and one additional preapical spine. The wings are often but not always dark. ♂: The clypeus is coarsely punctate, the interspaces between the punctures large, approximately one diameter apart. The forefemoral notch is markedly carinated on the anterior and posterior margin. The volsella is dark, the dorsal process narrow, about as wide as the volsellar corpus basally.

##### Description of male.

Body length: 5.3–7.8 mm.

Head. Mandibular inner margin with one rectangular tooth and distinct furrow next to tooth distally. Clypeus convex, slightly elevated and steeply declining ventrally, top in apical third or in midlength; basomedian area large, sparsely punctate, more sparsely punctate medially than laterally in some specimens; bevel convex, conspicuously decreasing toward clypeal lip, variable in size, shiny with several larger punctures; lip distinctly sinuate, conspicuously produced medially in most specimens, with distinct lateral corner, lip separated from bevel by variable groove; WML:LCL = 1.2–1.3, WCL:WML = 2.3–2.4. Supraclypeal area slightly concave, distinctly punctate, punctures ill-defined, interspaces microsculptured, dull. Supraantennal tubercle small, but distinctly elevated on inner side, shiny. Antenna relatively short, LA3:WA3 = 1.5–1.6, LA5:WA5 = 1.9–2.1. Frons uniformly punctate, punctures well defined, less than one diameter apart, interspaces variable in size; frontal median line distinct, narrow, finely impressed. Vertex punctate, punctures relatively large, well defined, less than half to two diameters apart, interspaces slightly microsculptured, slightly shiny to dull. Vertex setae short, semierect medially, but nearly erect close to inner eye margin, less than 1 × MOD, postocellar impression distinct, shallow, sparsely punctate, punctures relatively large, open, widely V to Y-shaped; vertex slightly to moderately wider than long; WV:LV = 1.1-1.5. Gena dorsally well developed.

Mesosoma. Scutum without distinct anterior impression; scutum and scutellum densely punctate, punctures well defined, most punctures less than half diameter apart, interspaces distinct, microsculptured, slightly shiny to dull, setae about 1.0 × MOD or less. Mesopleuron rugose to densely punctate, most punctures in ventral half well defined, interspaces coarsely microsculptured to rugose, slightly shiny to dull; hypoepimeral area coarsely rugose, without distinct punctures, with distinct longitudinal ridges in most specimens, dull; ventral part of mesopleuron with punctures large, well defined (ill defined in small specimens), interspaces finely microsculptured, slightly shiny. Propodeal dorsum coarsely sculptured, longitudinally ridged, irregularly ridged, or areolate; side longitudinally ridged, ridges well developed, interspaces microsculptured, dull; posteromedial margin of dorsum slightly elevated or not elevated, marginal ridges fine, variably sinuous, horizontal, with ventromedially directed medially. Legs densely punctate, punctures small; forefemoral notch small, but relatively deep, semicircular, shorter than distance that separates it from forefemoral base, proximal margin relatively sharp, elevated over distal margin, central part of notch slightly elevated, anterior and posterior margins lined by faint ledge, notch surface without distinct setae, coarsely microsculptured, dull. Wings moderately infumate, but relatively pale in some specimens, veins brown.

Metasoma. Silvery apical faciae of terga I-III faintly developed, often absent. Apical depressions of all terga shallow, densely micropunctate, nearly as densely as more anterior parts. Terga I-III densely and distinctly micropunctate, punctures ill defined, interspaces microsculptured, slightly shiny; sculpture of terga IV-VII coarser than on previous terga, also punctation denser. Sterna uniformly punctate nearly like terga.

Coloration. Apical part of mandible red; terga I and II dark red, base of tergum II dark red in some specimens; apex of sternum I and base of sternum II dark red, infrequently specimens with three red metasomal segments also occur; distal tarsomeres dark red on ventral side; forefemoral notch black in most specimens, but dark red in a few. Tegulae reddish or brown translucent. Apical parts of all terga slightly translucent. Remaining body parts all black.

##### Description of female (holotype).

Body length: 7.8 mm.

Head. Clypeus distinctly convex, top at clypeal midlength; basomedian area relatively large, more sparsely punctate and punctures larger than on lateral section, interspaces between punctures basolaterally small, but getting sparser in mesoventral direction, interspaces well developed, but punctures mostly less than one diameter apart; bevel large, distinctly convex, as long as basomedian area both laterally and medially, not triangular, with sparse large punctures, not reaching base of clypeus, shiny; lip slightly arcuate, with lateral incision, separate from bevel by punctate groove, WML:LCL = 1.6, WCL:WML = 1.8. Supraclypeal area flat, distinctly punctate, punctures ill-defined, interspaces between punctures shiny to slightly shiny. Supraantennal tubercle slightly elevated on inner side. Antenna relatively short. Frons uniformly punctate, punctures well defined, about half diameter apart or less, interspaces slightly variable in size; frontal median line distinct, narrow, finely impressed. Vertex punctate, punctures well defined, less than half to one diameter apart, laterally sparser than medially, interspaces microsculptured, dull. Vertex setae short semierect, slightly longer laterally than medially, about 0.5 × MOD; postocellar impression distinct, shallow, V-shaped; vertex slightly wider than long; WV:LV = 1.3. Gena dorsally well developed.

Mesosoma. Scutum without distinct anterior impression; scutum and scutellum densely punctate, punctures well defined, less than half to one diameter apart, microsculptured, slightly shiny, setae about 1.0 × MOD or less. Mesopleuron coarsely microsculptured to finely rugose anteriorly, with indistinct punctures anteriorly; hypoepimeral area irregularly microsculptured to finely rugose, without punctures; ventral part of mesopleuron with punctures ill defined, less than one diameter apart, interspaces small, slightly shiny. Propodeal dorsum finely irregularly longitudinally ridged, interspaces rugose; propodeal side irregularly, but distinctly longitudinally ridged, ridges inconspicuous basally, interspaces microsculptured, dull; posteromedial margin of dorsum slightly elevated, marginal ridges directed ventromedially toward groove on posterior side. Legs densely punctate, punctures small; forebasitarsal rake reddish, with two apical spines, one preapical, and two additional ones. Wings slightly infumate, veins brown.

Metasoma. Terga I-III with silvery apical fasciae inconspicuously developed. Apical depressions of all terga shallow, with distinct micropunctures, on terga I-II evanescent in microsculpture. Terga I-III densely, finely micropunctate, punctures ill defined but distinct, interspaces microsculptured, slightly shiny; sculpture of tergum III-V distinctly coarser than on previous terga, also punctures slightly denser. Pygidium sparsely punctate, punctures ill defined, interspaces microsculptured, dull. Central part of sternum II with several large punctures, interspaces microsculptured, shiny; lateral part slightly shiny, densely micropunctate; remaining sterna with uniform sculpture similar to that of sternum II, but more or less reduced laterally.

Coloration. Central part of mandible, three distal tarsomeres, terga and sterna I-III red. Tegulae brown translucent. Apical parts of terga I-III slightly translucent. Remaining body parts all black.

Variation of females: Body length: 6.7–9.3 mm. WV:LV = 1.3–1.4. Antenna, LA3:WA3 = 2.2–2.4, LA5:WA5 = 2.6–2.7. Very variable in coloration and sculpture. The holotype female is among the specimens with the finest sculpture. The mesopleuron is coarsely punctate to coarsely rugose; the hypoepimeral area is longitudinaly ridged, but also coarsely and uniformly rugose in some specimens. The propodeal dorsum is longitudinaly ridged, but also coarsely and uniformly rugose in some specimens (usually the hypoepimeral area and the propodeal dorsum with a comparable sculpture). The abdominal segments I-III equally red or dark red, but also two, or just one tergite red, and then sterna darker than terga.

##### Geographic distribution.

Austria, Czech Republic, Bulgaria, Germany, France, Greece, Hungary, Italy, Kyrgyzstan, Portugal, Slovakia, Switzerland and Turkey.

#### Tachysphex punctipleuris sp. n.

Taxon classificationAnimaliaHymenopteraCrabronidae

http://zoobank.org/83E83A71-268C-4693-9238-EB074A4231B3

Figs 8 , 21 , 27 , 49 , 69 , 76 , 102

##### Type material.

Holotype: ♂, Italy: “Italy Lombardia Valtellina, / Grosio 600 m NN 46,29 N / 10,26 E leg Schmid- / Egger 09.07.2006 I-valH // ♂ Tachysphex / pompiliformis (Pz.) / C form – punctate / Jakub Straka det.2008”. Holotype in ZSM. Paratypes: **Austria**: Innsbruck, 1 ♀, 20. vi. 1920, E. Clément lgt., ZSM; **Bulgaria**: Dolni Pasarel N, Lozenska Planina, 1 ♂, 10. viii. 2010, T. Ljubomirov lgt., IBER; Etropole W, Etropolska Planina, 1 ♂, 1. vii. 2010, T. Ljubomirov lgt., IBER, CCDB-05716-A12; **Germany**: Badberg, Kaiserstuhl, Baden-Württemberg, bad, 1 ♀, 16. vii. 2011, Ch. Schmid-Egger lgt., ZSM HYM 14828; Grissheim, Baden-Württemberg, gr, 1 ♂, 16. vii. 2011, ZSM HYM 14830; 1 ♂, 18. vii. 2012, Ch. Schmid-Egger lgt., ZSM HYM 17629; **Hungary**: Apaj env., 1 ♂, 20.-22. vii. 2015, D. Benda lgt. et coll., ZSM HYM 24760; Kesthely, 1 ♀, 23. v. 2014, A. Astapenková, J. Habermannová, J. Straka lgt., JSPC; Kunszentmiklos env., 2 ♀♀, 16. vii. 2013, J. Habermannová, J. Straka lgt., JSPC; Pákozd env., pasture steppe, 2 ♂♂, 10. vi. 2015, 2 ♂♂, 25. vi. 2015, M. Halada lgt., ZSM HYM 24756, 1 ♂, 3.-6. vi. 2015, D. Benda, J. Straka lgt., ZSM HYM 24758, JSPC, OLML; Velencei-tó env., 1 ♀, 4.-9. vi. 2013, D. Benda, P. Bogusch, J. Straka lgt., 1 ♂, 23. v. 2014, A. Astapenková, J. Habermannová, J. Straka lgt., 1 ♂, 3.-6. vi. 2015, D. Benda, J. Straka lgt., ZSM HYM 24759, JSPC; **Italy**: Grosio, Valtellina, Lombardia prov., 3 ♂♂, 1 ♀, 9. vii. 2006, Ch. Schmid-Egger lgt., CSE; Monte/Castel, Veneto prov., I-venA, 1 ♂, 17. vii. 2011, Ch. Schmid-Egger lgt., ZSM HYM 14831; Osis/Eyrs, Vinschgau, Trentino-Alto Adige prov., 1 ♂, 8. vi. 2007, Ch. Schmid-Egger lgt., CSE; Rivera, Lignano, 1 ♂, 3. vii. 2015, M. Kafka lgt., JSPC; **Slovenia**: Narin, 1 ♂, 6. vii. 2015, M. Kafka lgt., JSPC; **Turkey**: between Güvern and Çerkes, 1 ♂, 18. vi. 2006, E. Scheuchl lgt., CSE.

##### Additional material examined.

**Bulgaria**: Krushare E, 1 ♀, 6. vii. 2012, T. Ljubomirov lgt., IBER; Milanovo W, 1 ♂, 7. vii. 2015, T. Ljubomirov lgt., IBER; Dätzberg, Gellmersbach, Heilbronn, Baden-Württemberg, G2, 2 ♀♀, 20. vi. 1995, Ch. Schmid-Egger lgt., CSE; Keiserstuhl, Baden-Württemberg, 1 ♂, 19. viii. 1984, Ch. Schmid-Egger lgt., CSE; **Italy**: Eita 2 km N, Bormio 16 km SW, Valtellina, Lombardia prov., 1 ♂, 9. vii. 2006, Ch. Schmid-Egger lgt., CSE; Grosio, Valtellina, Lombardia prov., 1 ♂, 9. vii. 2006, Ch. Schmid-Egger lgt., CSE; Lago Rovine, Piemonte 26 km SW Cuneo, I-mari1, 1 ♂, 17. vi. 2009, Ch. Schmid-Egger lgt., CSE; San Benedetto Belbo, Province of Cuneo, 1 ♂, 15. vi. 2003, 1 ♂, 22. vii. 2008, 1 ♀, 25. viii. 2008, G. Pagliano lgt., ZSM; Sirino Mt., Basilicata prov., 1 ♂, 26. vi. 1998, M. Generani, P. L. Scaramozzino lgt., WSKC; Tartsch, Bolzano, 1 ♂, 28. vii. 1989, J. Tiefenthaler lgt., OLML; Terenten, Bolzano, 1 ♂, 1 ♀, 14.-28. ix. 1982, F. Parré lgt., OLML.

##### Diagnosis.

*Tachysphex punctipleuris* sp. n. is difficult to distinguish from *Tachysphex dimidiatus* (Panzer), *Tachysphex jokischianus* (Panzer) and *Tachysphex pompiliformis* (Panzer). It is exceptionally variable in most characters, and no single character alone can distinguish this species from the similar species. It possesses the following combination of characters: ♀: Clypeus slightly convex, the most elevated point not well defined, located approximately at clypeal midlength. Clypeal bevel variable, reaching the clypeal base in some specimens but well separated from it in others. Clypeal lip in fresh specimens sinuate, with ill-defined emargination medially. Vertex setae short, uniformly semierect medially as well as laterally. Postocellar impression well developed, deep, an open widely Y-shaped, or the posterior margin of the impression sinuous. Head nearly round, with uniformly, densely punctate frons. Gena robust. Punctures of mesopleuron variable but distinct and well defined in some specimens. Dorsolateral cuticular projection of propodeal spiracle arcuate to semicircular and usually all dark. Propodeal side uniformly longitudinally ridged, except in small specimens. Punctures of central part of tergum IV and V ill defined, interspaces between the punctures large, distinct, slightly shiny to shiny. Pygidium wide and shiny in most specimens, similar to *Tachysphex jokischianus* (Panzer). ♂: Clypeus slightly convex. Clypeal bevel large, densely punctate, interspaces between punctures small, punctures less than one diameter apart. Vertex approximately as wide as long. Gena robust. Supraclypeal area flat, supraantennal tubercle slightly elevated ventromedially. Mesopleuron laterally and ventrally distinctly punctate (punctures distinct also on hypoepimeral area in some specimens), punctures well defined, interspaces between the punctures distinct, slightly shiny to shiny on most parts. Forefemoral notch small, inconspicuously carinated, anterior carina distinct, black to dark red in coloration. Terga sparsely micropunctate, with variable interspaces between punctures. Volsella light brown, dorsal process basally wider than volsellar corpus.

##### Description of female.

Body length: 6.4–9.2 mm.

Head. Clypeus distinctly convex, top at clypeal midlength; basomedian area relatively large, about two fifths of bevel in space, as densely, uniformly punctate as lateral section; bevel large, slightly convex, obtusely triangular, with sparse large punctures, reaching base of clypeus in some specimens, but distantly separated from it in others, shiny; lip slightly sinuate with small median emargination and with small, but well developed lateral incision with rectangular corner, lip separate from bevel by punctate groove, WML:LCL = 1.5–1.7, WCL:WML = 1.8. Supraclypeal area flat, densely and distinctly punctate, punctures ill-defined, interspaces between punctures shiny to slightly shiny. Supraantennal tubercle small, slightly elevated on inner side. Antenna relatively short, LA3:WA3 = 2.2–2.4, LA5:WA5 = 2.6–2.7. Frons uniformly punctate, punctures well defined, less than one diameter apart, interspaces variable in size; frontal median line distinct, narrow, well impressed. Vertex relatively uniformly punctate, punctures well defined, less than one diameter apart, interspaces between punctures relatively uniform in size, unsculptured, shiny. Vertex setae short, semierect everywhere, distinctly shorter than 1 × MOD; postocellar impression well developed, relatively deep, open widely Y-shaped to sinuous; vertex slightly to moderately wider than long; WV:LV = 1.2–1.6. Gena dorsally well developed.

Mesosoma. Scutum without distinct anterior impression; scutum and scutellum densely punctate, punctures well defined, half to one diameter apart, interspaces slightly microsculptured, shiny, setae about 1.0 × MOD. Mesopleuron variable, distinctly, densely punctate, punctures well developed, interspaces microsculptured, slightly shiny to dull; in some specimens mesopleuron with ill-defined punctures, or with indistinct punctures in posterior and dorsal part; hypoepimeral area dull, with distinct punctures, or coarsely microsculptured, or finely longitudinally ridged, dull; ventral part of mesopleuron with punctures well developed in most specimens, less than one diameter apart, interspaces shiny. Propodeal dorsum relatively finely, irregularly rugose, but with distinct longitudinal ridges in some specimens; propodeal side irregularly, incompletely longitudinally ridged, ridges well developed in large specimens, completely absent in some small specimens, dull; posteromedial margin of propodeal dorsum slightly elevated, slightly produced between marginal ridges, marginal ridges directed ventromedially toward groove on posterior side. Legs densely punctate, punctures small; forebasitarsal rake pale reddish to yellowish, with three apical spines, one preapical spine, and two additional spines. Wings slightly yellowish, veins brown.

Metasoma. Terga I-III with silvery apical fasciae. Apical depressions of terga shallow, with micropunctures evanescent in microsculpture. Terga I-III sparsely, finely micropunctate, punctures ill defined, interspaces microsculptured, shiny to slightly shiny; sculpture of tergum IV-V slightly coarser than on previous terga, punctures slightly denser. Pygidium sparsely punctate, punctures ill defined, interspaces microsculptured, slightly shiny. Central part of sternum II with several large punctures, interspaces microsculptured, shiny; lateral part slightly shiny, densely micropunctate; remaining sterna with uniform sculpture similar to that of sternum II, but more or less reduced laterally.

Coloration. Central part of mandibles, three distal tarsomeres, terga and sterna I-III red. Tegulae brown translucent. Apical parts of terga I-III slightly translucent. Remaining body parts all black.

##### Description of male (holotype).

Body length: 6.7 mm.

Head. Clypeus slightly convex, uniformly curved, top at clypeal midlength; basomedian area large, densely punctate; bevel small, slightly convex, shiny, with several large punctures; lip conspicuously arcuate, well developed medially, with small lateral corner, lip separated from bevel by distinct groove with large punctures; WML:LCL = 1.1, WCL:WML = 2.4. Supraclypeal area flat, distinctly punctate, interspaces between punctures shiny. Supraantennal tubercle small slightly elevated on inner side. Frons uniformly, finely punctate, punctures well defined, one to less than half diameter apart, interspaces variable in size; frontal median line distinct, narrow and finely impressed. Vertex punctate, punctures well defined, less than half to one diameter apart, interspaces unsculptured, shiny. Vertex setae short, semierect, less than 1 × MOD; postocellar impression well developed, relatively deep, open, widely Y-shaped; vertex moderately wider than long; WV:LV = 1.5. Gena dorsally well developed.

Mesosoma. Scutum without distinct anterior impression; scutum and scutellum densely, relatively finely punctate, punctures well defined, most punctures less than one diameter apart, interspaces distinct, unsculptured, shiny, setae about 1.0 × MOD or less. Mesopleuron distinctly punctate throughout, most punctures well defined, interspaces finely microsculptured, slightly shiny; also hypoepimeral area densely punctate, interspaces microsculptured; ventral part of mesopleuron with punctures well defined, interspaces shiny. Propodeal dorsum coarsely sculptured, ridged and areolate; propodeal side longitudinally ridged, ridges well developed, microsculptured, dull; posteromedial margin of propodeal dorsum inconspicuously elevated, marginal ridges directed ventromedially toward groove on posterior side. Legs densely punctate, punctures small; forefemoral notch relatively shallow, small, shorter than distance that separates it from forefemoral base, central part of notch slightly elevated, anterior margin lined by faint ledge, posterior margin lined by shiny, nearly glabrous area, notch surface without distinct setae, microsculptured, dull. Wings slightly infumate, veins brown.

Metasoma. Terga I-III with silvery apical faciae. Apical depressions of terga shallow, micropunctate. Terga I-III sparsely, distinctly micropunctate, punctures ill defined, interspaces microsculptured, shiny; sculpture of tergum IV-VII coarser than on previous terga, punctures slightly denser. Sterna uniformly punctate nearly like terga.

Coloration. Apical part of mandibles, tegulae, terga and sterna I-III red; distal tarsomeres and forefemoral notch in center dark reddish. Apical parts of terga and tegula translucent. Remaining body parts all black.

Variation of males: Mandibular inner margin with one distinct rectangular tooth and distinct furrow next to the tooth distally. Antenna relatively short, LA3:WA3 = 1.5–1.6, LA5:WA5 = 1.9–2.1. Volsella light brown, ventral setae pointing in various directions; dorsal process slightly wider than corpus in most specimens. Sculpture of mesopleuron very variable, densely to sparsely punctate, punctures mostly well defined, but indistinct in some specimens. Propodeum coarsely to finely sculptured, with or without longitudinal ridges. Forefemoral notch often black.

##### Geographic distribution.

Austria, Bulgaria, Germany, Hungary, Italy, Slovenia and Turkey.

##### Name derivation.

The species is named after its punctate mesopleuron in most males.

### Key to species of *Tachysphex austriacus* and *Tachysphex pompiliformis* subgroups of Europe and Turkey

♀♀

**Table:** 

1a	Gena dorsally inconspicuous, converging behind compound eyes (Figs 28, 32–34)	**2**
1b	Gena dorsally more robust (Figs 29–31, 35)	**5**
2a	Scutum and scutellum sparsely punctate, with large interspaces, punctures well developed, usually up to 1.5 diameter apart (more so in some specimens) (Fig. 38). Ventral part of mesopleuron with relatively large punctures, punctures and interspaces well developed (Fig. 50)	***Tachysphex austriacus* Kohl**
2b	Scutum and scutellum densely punctate, with small but distinct interspaces, punctures usually less than one diameter apart (only few interspaces larger in some specimens) (Figs cf. 39, 40). Ventral part of mesopleuron densely punctate, punctures small, ill defined (Fig. cf. 51)	**3**
3a	Wing membrane light yellowish (Fig. cf. 105). Clypeus only slightly convex, bevel small, basomedian area densely punctate, as long as 2/3 of clypeal length (Fig. 19)	***Tachysphex nobilis* sp. n.**
3b	Wing membrane infumate (Fig. 103). Clypeus distinctly convex, basomedian area about as long as bevel or shorter (Fig. 18)	**4**
3c	Character combination different	**5**
4a	Vertex distinctly wider than long (Fig. 32). Pygidium narrow (Fig. 89), interspaces between punctures unsculptured, bright shiny	***Tachysphex nigripennis* (Spinola)**
4b	Vertex about as long as wide or slightly wider than long (Fig. 34). Pygidium wider (Fig. cf. 90), densely microsculptured, dull	***Tachysphex opacus* F. Morawitz**
5a	Pygidium, mid- and hindtibiae light reddish (Fig. 105)	***Tachysphex ferrugineus* Pulawski**
5b	Pygidium and all tibiae dark	**6**
6a	Clypeus conspicuously elevated, transition between basomedian area and bevel relatively sharp, appearing angulated (Figs 13–14). Clypeal bevel flat, mirror-like shiny. Apical depression of tergum V impunctate medially (Figs 78, 81), sparsely, finely punctate laterally (Figs 79, 82), distinctly more shiny than basal part of tergum. Forebasitarsal rake pale, with three apical and one preapical spine	**7**
6b	Clypeus less elevated, basomedian area sharply separated from bevel in sculpture, but gradually changing into one anoher in convexity (Figs 15–18, 20–21). Clypeal bevel convex, at least basally, shiny. Apical depression of tergum V punctate or impunctate laterally, hardly distinguishable from basal part in sculpture, equally dull (Fig. cf. 85). Forebasitarsal rake dark or pale, with three or two apical and one preapical spine	**9**
7a	Several punctures on scutum and trochanters about two diameters apart	***Tachysphex prismaticus* Straka**
7b	Punctures on scutum and trochanters less than one diameter apart	**8**
8a	Mesopleuron finely punctate, punctures less distinct, evanescent in microsculpture (Fig. 42). Vertex finely punctate, with punctures contiguous medially, laterally more than one diameter apart (Fig. 36)	***Tachysphex hungaricus* sp. n.**
8b	Mesopleuron coarsely punctate, punctures relatively large and well developed ventrally (Fig. 43). Vertex coarsely punctate, punctures medially separated by distinct interspaces, interspaces slightly larger laterally than medially (Fig. 37)	***Tachysphex smissenae* sp. n.**
9a	Clypeal bevel distinctly triangular, reaching clypeal base at middle and separating basomedian area in two parts; when clypeal base punctate, punctures and interspaces larger than on lateral side of basomedian area (Figs 16, cf. 18, 21)	**10**
9b	Clypeal bevel distinctly separated from clypeal base by basomedian area, which is continuous from side to side, uniformly densely punctate, punctures much finer than on bevel (Figs 15, 17, 20)	**14**
10a	Clypeus markedly convex, most elevated point located between clypeal midlength and basal third of clypeus (Fig. 22). Vertex setae semierect medially, nearly erect, slightly shorter than 1 × MOD laterally. Dorsolateral cuticular projection of propodeal spiracle slightly arcuate to nearly straight, with apex reddish translucent	***Tachysphex dimidiatus* (Panzer)**
10b	Clypeus less convex, most elevated point not well defined, but appearing located near clypeal midlength (Fig. 23). Vertex setae shorter, about 0.5 × MOD, uniformly semierect medially and laterally. Dorsolateral cuticular projection of propodeal spiracle arcuate to semicircular, usually all dark	**11**
11a	Punctures of central part of terga IVand V small, ill defined; interspaces between punctures large, distinct, slightly shiny to shiny (Figs cf. 80, 83)	**12**
11b	Punctures of central part of terga IVand V larger, well defined, approximately 0.5–1.5 diameter apart, slightly shiny to dull (Figs 84, 85)	**13**
12	Similar and variable species, difficult to distinguish:
12a	Free margin of clypeus in fresh specimens sinuate, with small emargination medially (Fig. 21). Head appearing round in front view, frons uniformly, densely punctate. Vertex impression usually deeper. Dorsolateral cuticular projection of propodeal spiracle arcuate to semicircular, with dark apex. Propodeal side uniformly longitudinally ridged, except on small specimens. Punctures of mesopleuron variable, but more distinct and well developed in some specimens. Pygidium wider, slightly convex to partly flat and shinier in most specimens	***Tachysphex punctipleuris* sp. n.**
12b	Free margin of clypeus in fresh specimens arcuate or straight, without emargination medially (Fig. 16). Head appearing transversally oval in front view, frons irregularly punctate. Vertex impression usually shallower. Dorsolateral cuticular projection of propodeal spiracle slightly arcuate to nearly straight, with apex reddish and transparent. Propodeal side longitudinally ridged, ridges anteriorly inconspicuous or absent. Punctures of mesopleuron variable, but usually smaller and less distinct. Pygidium narrower, convex and largely microsculptured in most specimens	***Tachysphex dimidiatus* (Panzer)**
13a	All mesopleuron coarsely sculptured (Figs cf. 44, cf. 46, cf. 48). Hypoepimeral area of mesopleuron, and propodeal dorsum longitudinally ridged (propodeal dorsum sometimes coarsely sculptured, with indistinct ridges) (Figs cf. 46, 58. Tergum V, including apical depression, densely, distinctly punctate (Fig. 85). Pygidium narrower, with microsculptured integument (Fig. 90)	***Tachysphex pompiliformis* (Panzer)**
13b	Mesopleuron, including hypoepimeral area, finely sculptured (Fig. 45). Propodeal dorsum at most with basal ridges (Figs 57, 61). Apex of tergum V impunctate or finely, sparsely punctate (Fig. 84). Pygidium often wide, with convex and shiny surface (Fig. 88)	***Tachysphex jokischianus* (Panzer)**
14a	All mesopleuron coarsely sculptured (Figs cf. 44, cf. 46, cf. 48). Hypoepimeral area and propodeal dorsum and side longitudinally ridged (propodeal dorsum sometimes coarsely sculptured, but ridges indistinct) (Figs cf. 46, 58. Tergum V, including apical depression, densely and distinctly punctate (Fig. 85). Forebasitarsal rake reddish in fresh individuals, with two, well separated apical spines and one preapical spine	***Tachysphex pompiliformis* (Panzer)**
14b	Mesopleuron, including hypoepimeral area, punctate or finely sculptured (Fig. 45). Propodeal dorsum at most with basal ridges (Figs 56–57, 59–61). Apex of tergum V more sparsely and more finely punctate than in the central part (Figs 80, 83–84)	**15**
15a	Supraantennal tubercle rounded, distinctly punctate on top, in between and nearly at all sides; small impunctate area occurs only laterally (Fig. 106)	***Tachysphex bohemicus* sp. n.**
15b	Supraantennal tubercle prominent with relatively sharply delimited, shiny, nearly impunctate area laterally and at top (Figs 107, cf. 108)	**16**
16a	Apex of terga IV and V finely microsculptured, sparsely punctate to impunctate, contrasting with coarsely punctate central part of tergum IV, punctures of terga IV and V well developed (Fig. 84). Pygidium often wide, with distinctly convex, shiny surface (Fig. 88). Dorsolateral cuticular projection of propodeal spiracle arcuate to semicircular, with dark apex. Supraclypeal area with distinctly microsculptured interspaces between punctures, usually appearing dull (Fig. cf. 108). Propodeal dorsum without longitudinal ridges, ridged only basally, or with a few irregular ridges (Figs 57, 61)	***Tachysphex jokischianus* (Panzer)**
16b	Apex of tergum IV coarsely microsculptured, sparsely punctate (Fig. 83). Punctures of terga IV and V ill defined (Fig. 83). Pygidium of usual size, slightly convex, largely microsculptured, shiny at most in apical third (Figs cf. 87, 90). Dorsolateral cuticular projection of propodeal spiracle variable. Supraclypeal area with microsculptured interspaces between punctures but usually appearing shiny (Fig. 107). Propodeal dorsum variable but often with longitudinal ridges (Figs 56, 60)	**17**
17	Similar and variable species, difficult to distinguish:
17a	Clypeal free margin in fresh specimens sinuate, with small anterior emargination medially (Fig. 21). Head appearing round in front view, frons uniformly and densely punctate. Vertex impression usually conspicuous. Dorsolateral cuticular projection of propodeal spiracle arcuate to semicircular, with dark apex. Propodeal side uniformly longitudinally ridged, except in small specimens. Punctures of mesopleuron variable but more distinct and well defined in some specimens. Pygidium wider and shinier in most specimens	***Tachysphex punctipleuris* sp. n.**
17b	Clypeal free margin in fresh specimens arcuate or straight, without anterior emargination medially (Fig. 16). Head appearing transversally oval in front view, frons irregularly punctate. Vertex impression usually less conspicuous. Dorsolateral cuticular projection of propodeal spiracle slightly arcuate to nearly straight, with apex reddish transparent. Propodeal side longitudinally ridged, ridges anteriorly inconspicuous or absent. Punctures of mesopleuron variable but usually smaller and ill defined. Pygidium narrow and largely microsculptured in most specimens	***Tachysphex dimidiatus* (Panzer)**

♂♂

**Table:** 

1a	Surface of forefemoral notch slightly shiny to shiny, without elevated plate and ledge on margins and with distinct small setae (Fig. 62). Setae on ventral part of volsella uniformly directed ventrally or nearly so (Figs 91–92). (*Tachysphex austriacus* species subgroup)	**2**
1b	Surface of forefemoral notch microsculptured (finely so in some species), slightly elevated, with distinct ledge on inner margin (also on outer margin in most species), without distinct setae (Figs 63–65, 68). Setae on ventral part of volsella directed randomly outward and inward (Figs 94–102). (*Tachysphex pompiliformis* species subgroup)	**4**
2a	Central part of scutum sparsely punctate, punctures more than one diameter apart (Fig. cf. 38)	***Tachysphex austriacus* Kohl**
2b	Scutum densely punctate, puncture less than one diameter apart (Fig. cf. 39)	**3**
3a	Gena narrow (Figs cf. 25, cf. 28). Trochanteral venters with large, glabrous spaces between punctures. Ventral and lower part of mesopleuron coarsely punctate, punctures well defined. Vertex, propodeum and metasoma finely sculptured	***Tachysphex prismaticus* Straka**
3b	Gena robust (Fig. cf. 27). Trochanters densely punctate. Ventral and lower part of mesopleuron coarsely punctate (Fig. cf. 43), punctures well defined. Vertex (Fig. cf. 37), propodeum (Fig. cf. 55) and metasoma coarsely sculptured	***Tachysphex smissenae* sp. n.**
3c	Gena robust (Fig. cf. 27). Trochanters densely punctate. Ventral and lower part of mesopleuron finely and densely punctate, punctures ill defined (Fig. cf. 42). Vertex (Fig. 36), propodeum (Fig. 54) and metasoma finely sculptured	Tentative position of unknown male of ***Tachysphex hungaricus* sp. n.**
4a	Gena dorsally short, conspicuously converging behind compound eyes (Figs 25, cf. 32–34). Vertex distinctly wider than long	**5**
4b	Gena behind compound eyes more robust (Figs 24, 26–27). Vertex only slightly wider than long	**8**
5a	All tibia and tergum VII reddish (Fig. cf. 105)	***Tachysphex ferrugineus* Pulawski**
5b	All tibia and tergum VII dark	**6**
6a	All clypeus densely punctate, clypeal bevel indistinct (Fig. 7). Two or three basal metasomal terga and sterna light red. Forefemoral notch large, light reddish (Fig. 73, cf. 65, cf. 72)	***Tachysphex nobilis* sp. n.**
6b	Clypeal bevel distinct, sparsely punctate (Figs 1–6, 8–10). Metasoma black, or two basal terga dark red, sternum II almost always black. Forefemoral notch smaller, dark red, or black (Figs 66–67, 74)	**7**
7a	Forefemoral notch indistinctly microsculptured, shiny (Fig. 66). Clypeal lip wider than one third of clypeal width, projecting medially, its apical margin sinuate (Fig. 6). Mandibular inner margin with distinct small furrow distally of inner tooth (Fig. cf. 12)	***Tachysphex nigripennis* (Spinola)**
7b	Forefemoral notch densely microsculptured, dull (Fig. 67). Clypeal lip narrower, about as wide as one third of clypeal width, uniformly arcuate to nearly straight (Fig. 10). Mandibular inner margin shallowly emarginated distally of inner tooth, without furrow (Fig. 11)	***Tachysphex opacus* F. Morawitz**
8a	Supraantennal tubercle rounded, distinctly punctate on top, in-between and nearly at all sides; small impunctate area only laterally (Fig. 106)	***Tachysphex bohemicus* sp. n.**
8b	Supraantennal tubercle prominent, relatively sharply delimited, shiny, nearly impunctate area dorsally and laterally (Figs 107–108)	**9**
9a	Mesopleuron coarsely irregularly ridged (Figs 44, 48) or coarsely punctate (Fig. 49). Forefemoral notch black or dark red (Fig. 68)	**10**
9b	Mesopleuron finely sculptured (Figs cf. 45, cf. 47). Forefemoral notch light red (Figs 64–65, 69)	**12**
10a	Body black, silvery apical fasciae present on terga I-IV (Fig. 104)	***Tachysphex cretensis* sp. n.**
10b	At least basal metasomal terga red, silvery apical fasciae present on terga I-III	**11**
11a	Clypeus coarsely punctate, interspaces between punctures large, punctures about one diameter apart (Fig. 9). Clypel lip slightly arcuate (Fig. 9). Dorsal part of mesopleuron, especially hypoepimeral area more or less conspicuously longitudinally ridged (Figs cf. 44, 48). Forefemoral notch markedly carinated on both anterior and posterior margin (Fig. 68). Volsella dark, dorsal process narrow, about as wide as volsellar corpus (Fig. 101)	***Tachysphex pompiliformis* (Panzer)**
11b	Clypeus finely and densely punctate, interspaces between punctures small, punctures less than one diameter apart (Fig. 8). Clypeal lip conspicuously prominent (Fig. 8). Dorsal part of mesopleuron distinctly punctate, punctures well developed, hypoepimeral area also with punctures in some specimens (Fig. 49). Forefemoral notch small, inconspicuously carinated, carina well developed only on anterior margin (Fig. 69). Volsella light brown, dorsal process basally wider than volsellar corpus (Fig. 102)	***Tachysphex punctipleuris* sp. n.**
12a	Mesopleuron laterally and ventrally with well-defined punctures (punctures distinct also on hypoepimeral area in some specimens), interspaces distinct, light shiny to shiny on most parts (Fig. 49). Forefemoral notch small and relatively shallow, indistinctly carinated, carina well developed only on anterior margin (Figs 69, 76). Clypeal lip conspicuously prominent (Fig. 8)	***Tachysphex punctipleuris* sp. n.**
12b	Mesopleuron laterally and ventrally with ill-defined punctures, when interspaces distinct, then microsculptured, slightly shiny to dull. Forefemoral notch larger and deeper, distinctly carinated on anterior and posterior margin of notch (Figs cf. 63, 64–65). Clypeal lip of usual form, slightly arcuate (Fig. cf. 9)	**13**
13a	Supraclypeal area forming groove, supraantennal tubercle distinctly elevated ventromedially (Fig. cf. 108). Forefemoral notch light red, large, its diameter usually longer than distance that separates it from forefemoral base (Fig. 65). Propodeal side densely ridged basally in most specimens (Fig. cf. 45). Posteromedial margin of propodeal dorsum slightly elevated, with nearly parallel ridges that separate dorsum from groove on posterior surface (Fig. 61). Terga densely micropunctate	***Tachysphex jokischianus* (Panzer)**
13b	Supraclypeal area flat, supraantennal tubercle slightly elevated ventromedially (Fig. cf. 107). Forefemoral notch variable, usually smaller, light red to red (Fig. 64). Propodeal side variable but with ridges disappearing anteriorly in most specimens. Posteromedial margin of propodeal dorsum elevated and produced between ridges, ridges directed ventromedially toward groove on posterior surface (Fig. 60). Terga sparsely micropunctate with variable interspaces	***Tachysphex dimidiatus* (Panzer)**

## Discussion

Morphological variation in *Tachysphex pompiliformis* sensu Pulawski (1971) was found to be extensive (Pulawski 1971). Some species of this group have already been separated by Pulawski himself (Pulawski 1971) or later recognized as valid (Straka 2004, 2005). Present recognition of fourteen species and two species subgroups may appear surprising, but this variation is correlated with ecological and biogeographic data. Not all species are ubiquitous. Only two reach Scandinavia (*Tachysphex jokischianus* and *Tachysphex dimidiatus*). Some species occur predominantly on sandy or loess habitats (*Tachysphex jokischianus*, *Tachysphex nigripennis*, and *Tachysphex nobilis*). This uneven distribution have suggested the presence of different species within a group for a long time, validated in the present study. These species delimitations are supported by morphological characters.

## Figures

**Figures 1–12.**
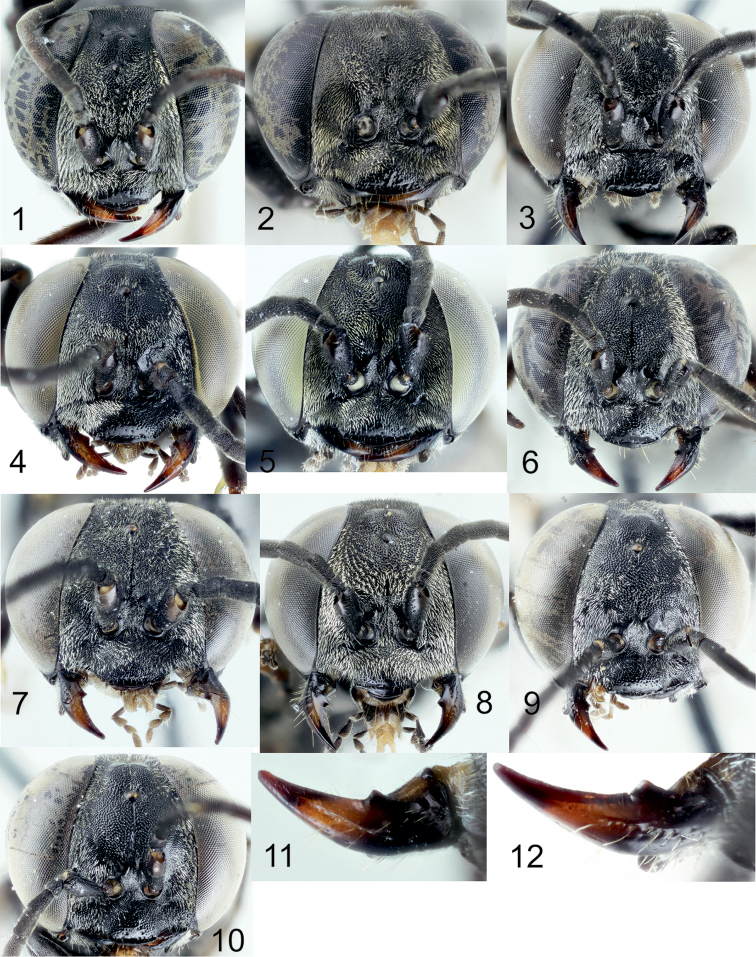
Head of male, front view, and mandibles. **1** *Tachysphex smissenae* sp. n. **2** *Tachysphex bohemicus* sp. n. **3** *Tachysphex cretensis* sp. n. **4** *Tachysphex dimidiatus* **5** *Tachysphex jokischianus* **6** *Tachysphex nigripennis* **7** *Tachysphex nobilis* sp. n. **8** *Tachysphex punctipleuris* sp. n. **9** *Tachysphex pompiliformis* **10** *Tachysphex opacus* **11** *Tachysphex opacus*, mandible, frontal view **12** *Tachysphex pompiliformis*, mandible, frontal view.

**Figures 22–23.**
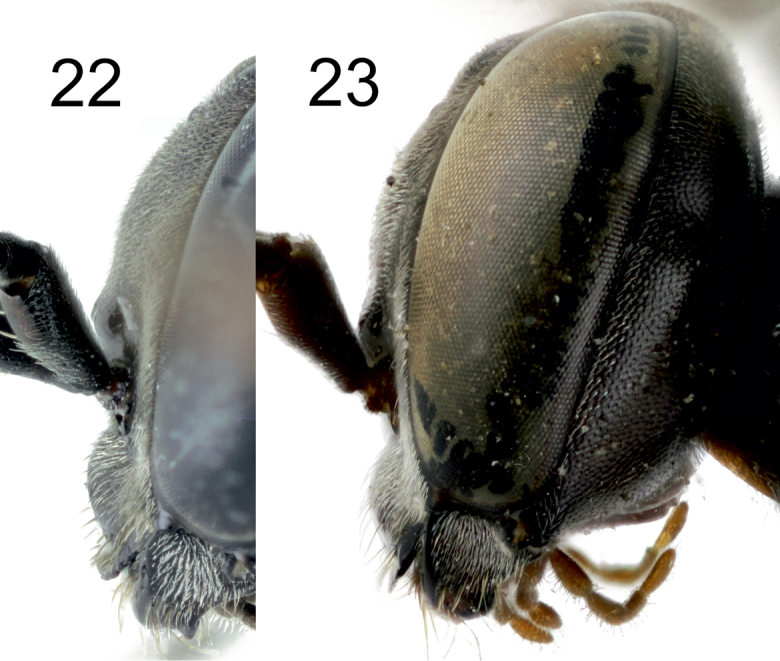
Head of female, lateral view of clypeus. **22** *Tachysphex dimidiatus* **23** *Tachysphex jokischianus*.

**Figures 24–27.**
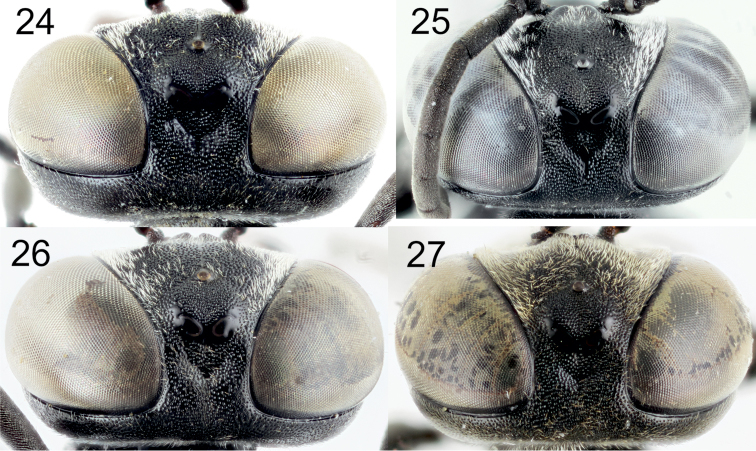
Gena of male. **24** *Tachysphex dimidiatus* **25** *Tachysphex opacus* **26** *Tachysphex pompiliformis* **27** *Tachysphex punctipleuris*.

**Figures 28–35.**
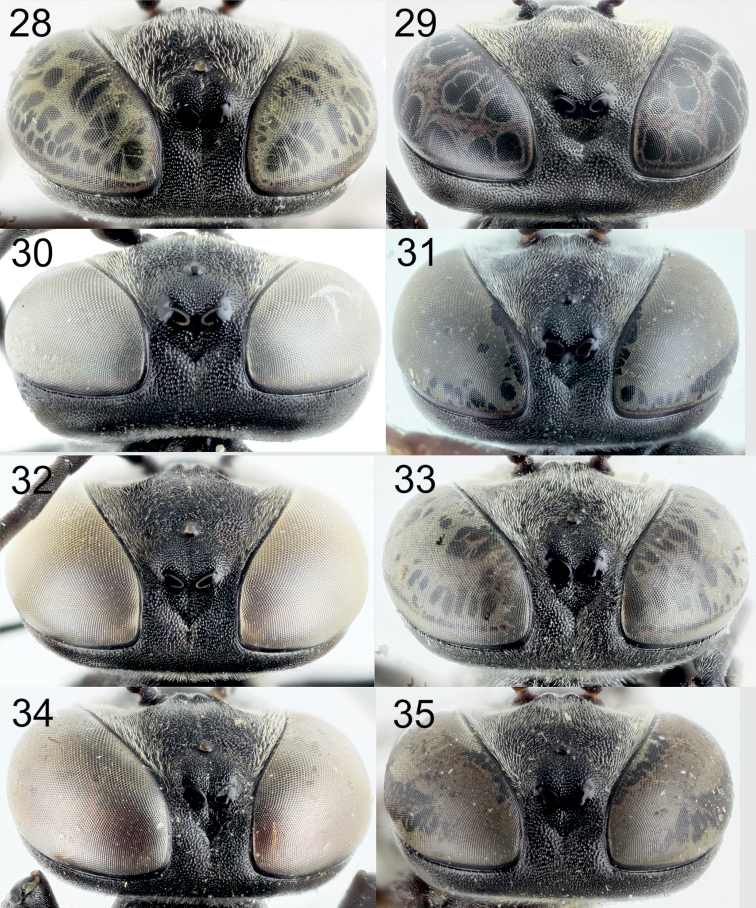
Gena of female. **28** *Tachysphex austriacus* **29** *Tachysphex bohemicus* sp. n. **30** *Tachysphex dimidiatus* **31** *Tachysphex jokischianus* **32** *Tachysphex nigripennis* **33** *Tachysphex nobilis* sp. n. **34** *Tachysphex opacus* **35** *Tachysphex pompiliformis*.

**Figures 38–40.**
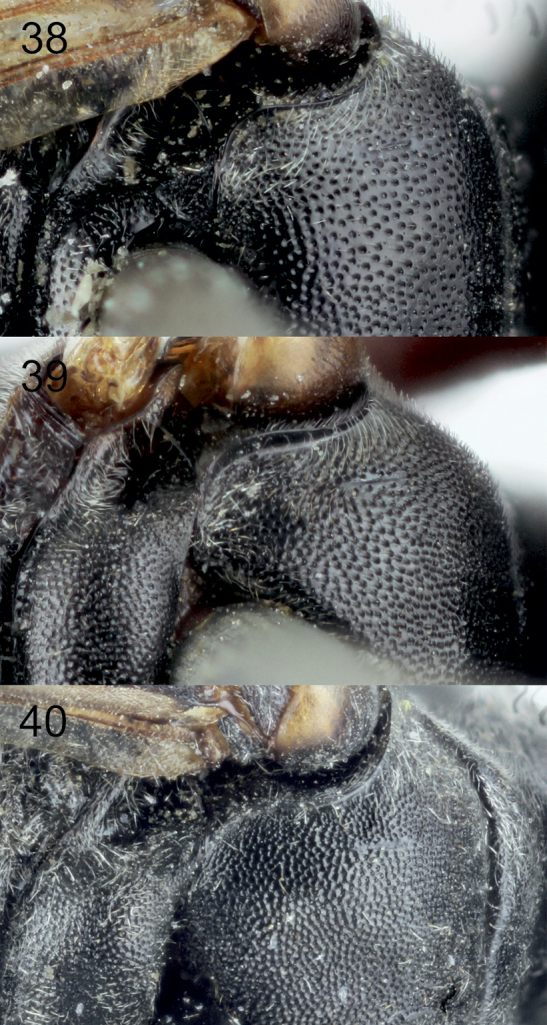
Scutum of female, dorsal view. **38** *Tachysphex austriacus* **39** *Tachysphex jokischianus* **40** *Tachysphex nobilis* sp. n.

**Figures 41–49.**
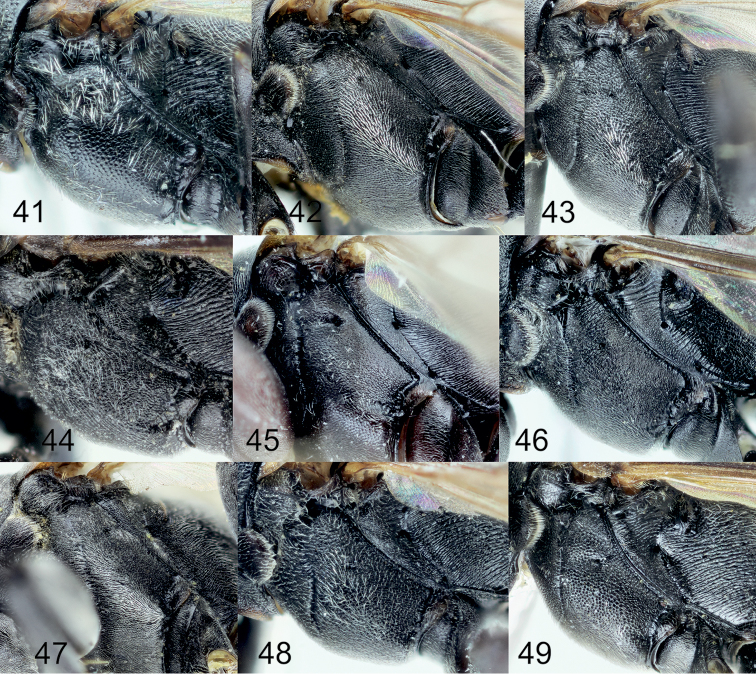
Mesopleuron, lateral view, sculptures. **41** *Tachysphex austriacus*, male **42** *Tachysphex hungaricus* sp. n., female **43** *Tachysphex smissenae* sp. n., female **44** *Tachysphex cretensis* sp. n., male **45** *Tachysphex jokischianus*, female **46** *Tachysphex nigripennis*, female **47** *Tachysphex nobilis* sp. n., female **48** *Tachysphex pompiliformis*, male **49** *Tachysphex punctipleuris* sp. n., male.

**Figures 50–52.**
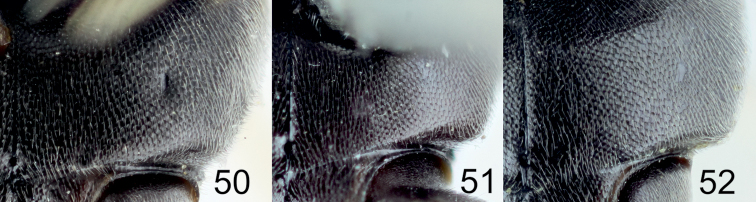
Mesopleuron, ventral view, sculptures, female. **50** *Tachysphex austriacus* **51** *Tachysphex jokischianus* **52** *Tachysphex pompiliformis*.

**Figures 53–61.**
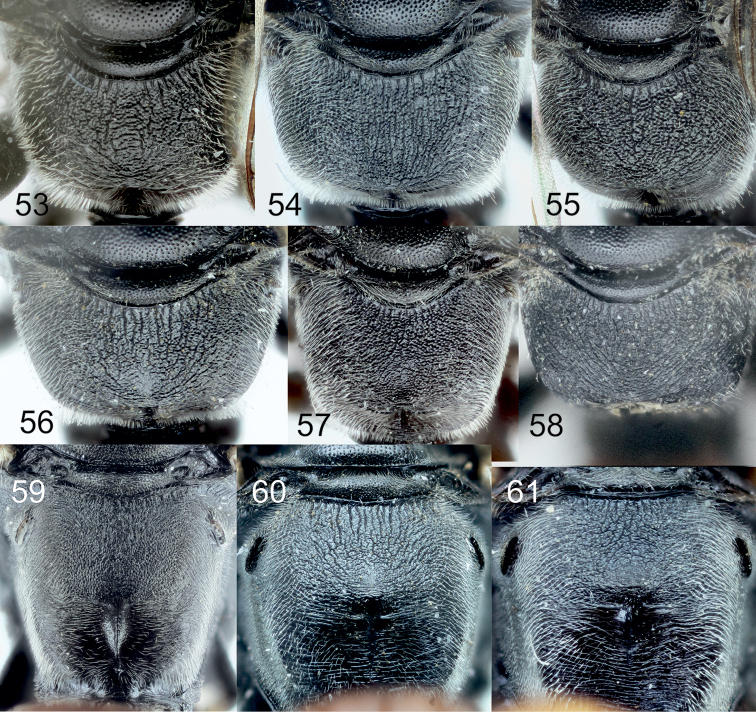
Propodeum, dorsal and posterior view, female. **53** *Tachysphex austriacus*, dorsal view **54** *Tachysphex hungaricus* sp. n., dorsal view **55** *Tachysphex smissenae* sp. n., dorsal view **56** *Tachysphex dimidiatus*, dorsal view **57** *Tachysphex jokischianus*, dorsal view **58** *Tachysphex pompiliformis*, dorsal view **59** *Tachysphex bohemicus* sp. n., posterior view **60** *Tachysphex dimidiatus*, posterior view **61** *Tachysphex jokischianus*, posterior view.

**Figures 62–76.**
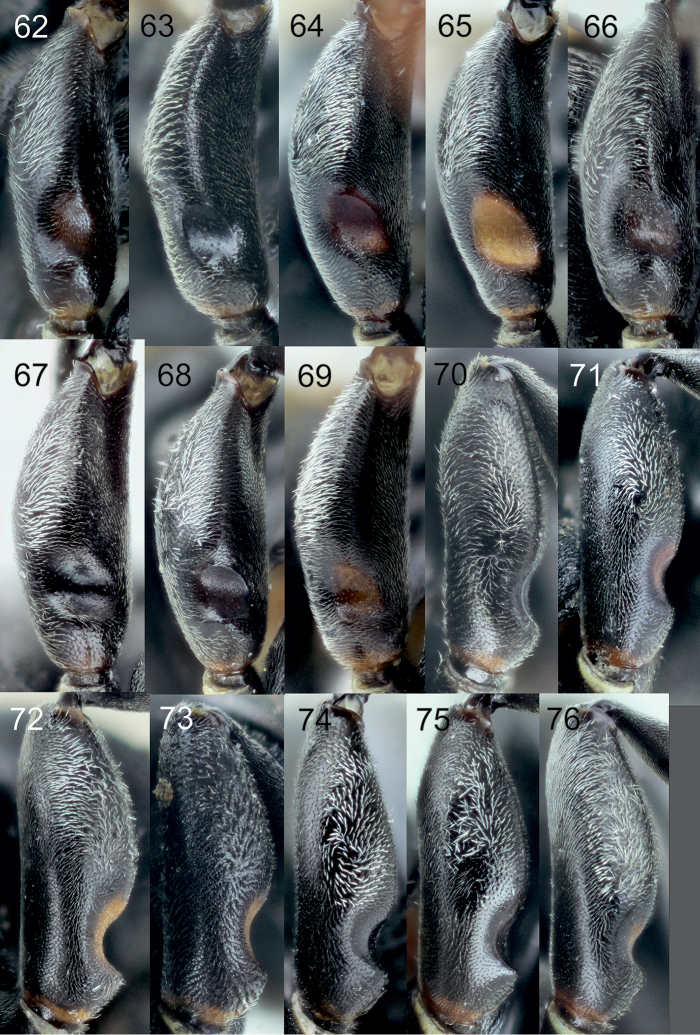
Forefemoral notch of male, ventral and lateral view. **62** *Tachysphex austriacus*, ventral view **63** *Tachysphex bohemicus* sp. n., ventral view **64** *Tachysphex dimidiatus*, ventral view **65** *Tachysphex jokischianus*, ventral view **66** *Tachysphex nigripennis*, ventral view **67** *Tachysphex opacus*, ventral view **68** *Tachysphex pompiliformis*, ventral view **69** *Tachysphex punctipleuris* sp. n., ventral view **70** *Tachysphex bohemicus* sp. n., lateral view **71** *Tachysphex dimidiatus*, lateral view **72** *Tachysphex jokischianus*, lateral view **73** *Tachysphex nobilis* sp. n., lateral view **74** *Tachysphex opacus*, lateral view **75** *Tachysphex pompiliformis*, lateral view **76** *Tachysphex punctipleuris* sp. n., lateral view.

**Figures 77–85.**
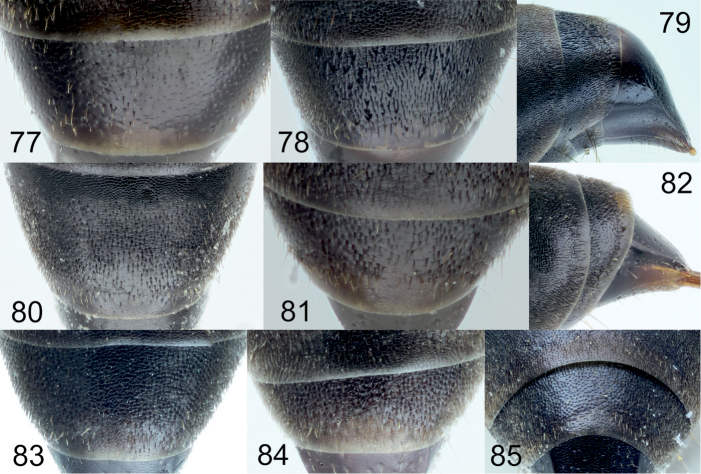
Tergum V, sculpture, female. **77** *Tachysphex austriacus*, dorsal view **78** *Tachysphex hungaricus* sp. n., dorsal view **79** *Tachysphex hungaricus* sp. n., lateral view **80** *Tachysphex bohemicus* sp. n., dorsal view **81** *Tachysphex smissenae* sp. n., dorsal view **82** *Tachysphex smissenae* sp. n., lateral view **83** *Tachysphex dimidiatus*, dorsal view **84** *Tachysphex jokischianus*, dorsal view **85** *Tachysphex pompiliformis*, posterior view.

**Figures 86–90.**
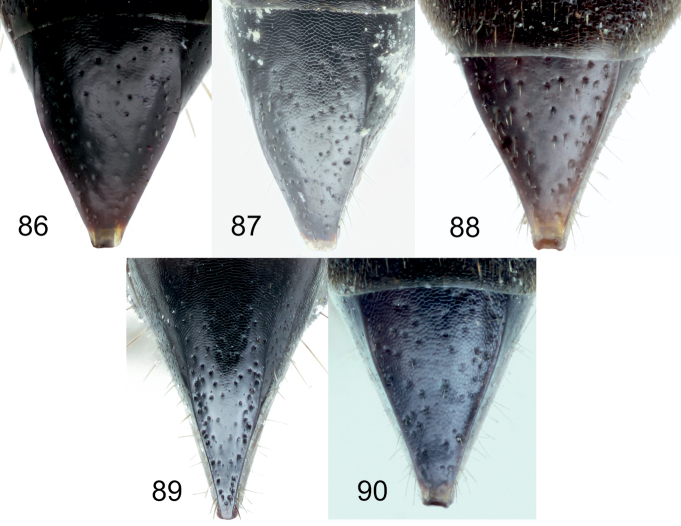
Pygidium, female. **86** *Tachysphex austriacus* **87** *Tachysphex bohemicus* sp. n. **88** *Tachysphex jokischianus* **89** *Tachysphex nigripennis* **90** *Tachysphex pompiliformis*.

**Figures 91–102.**
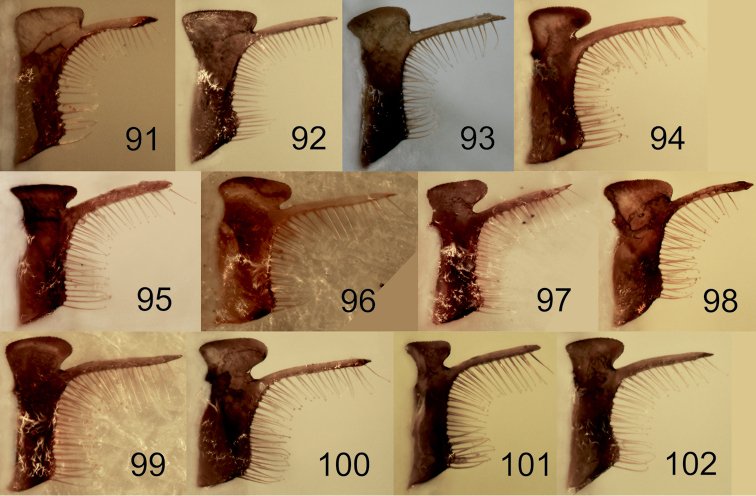
Volsella, lateral view. **91** *Tachysphex austriacus* **92** *Tachysphex smissenae* sp. n. **93** *Tachysphex bohemicus* **94** *Tachysphex cretensis* sp. n. **95** *Tachysphex dimidiatus* **96** *Tachysphex ferrugineus* **97** *Tachysphex jokischianus* **98** *Tachysphex nigripennis* **99** *Tachysphex nobilis* sp. n. **100** *Tachysphex opacus* **101** *Tachysphex pompiliformis* **102** *Tachysphex punctipleuris* sp. n.

**Figures 103–105.**
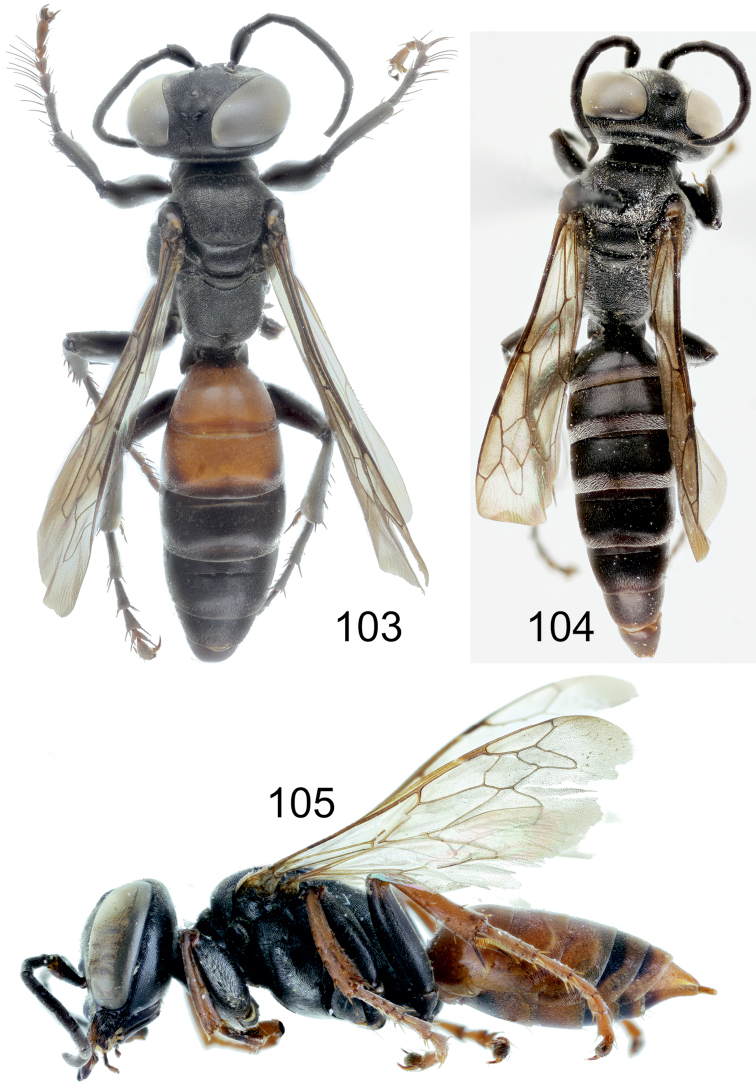
Habitus, dorsal and lateral view. **103** *Tachysphex nigripennis*, female, dorsal view **104** *Tachysphex cretensis* sp. n., male, dorsal view **105** *Tachysphex ferrugineus*, female, lateral view.

**Figures 106–108.**
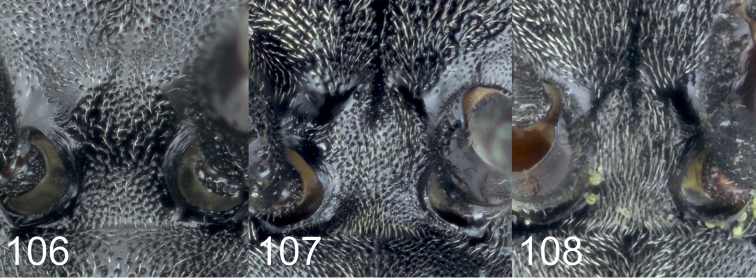
Supraantennal tubercles, frontal view. **106** *Tachysphex bohemicus* sp. n. **107** *Tachysphex dimidiatus* **108** *Tachysphex nobilis* sp. n.

## Supplementary Material

XML Treatment for Tachysphex austriacus

XML Treatment for Tachysphex hungaricus

XML Treatment for Tachysphex prismaticus

XML Treatment for Tachysphex smissenae

XML Treatment for Tachysphex bohemicus

XML Treatment for Tachysphex cretensis

XML Treatment for Tachysphex dimidiatus

XML Treatment for Tachysphex ferrugineus

XML Treatment for Tachysphex jokischianus

XML Treatment for Tachysphex nigripennis

XML Treatment for Tachysphex nobilis

XML Treatment for Tachysphex opacus

XML Treatment for Tachysphex pompiliformis

XML Treatment for Tachysphex punctipleuris
